# The Developmental Delay of Seedlings With Cotyledons Only Confers Stress Tolerance to *Suaeda aralocaspica* (Chenopodiaceae) by Unique Performance on Morphology, Physiology, and Gene Expression

**DOI:** 10.3389/fpls.2022.844430

**Published:** 2022-06-06

**Authors:** Jing Cao, Xiaorong Li, Ling Chen, Meixiang He, Haiyan Lan

**Affiliations:** Xinjiang Key Laboratory of Biological Resources and Genetic Engineering, College of Life Science and Technology, Xinjiang University, Urumqi, China

**Keywords:** cotyledon, developmental delay, seedling growth, stress tolerance, *Suaeda aralocaspica*

## Abstract

Cotyledons play an important role in seedling establishment, although they may just exist for a short time and become senescent upon the emergence of euphylla. So far, the detailed function of cotyledons has not been well understood. *Suaeda aralocaspica* is an annual halophyte distributed in cold deserts; its cotyledons could exist for a longer time, even last until maturity, and they must exert a unique function in seedling development. Therefore, in this study, we conducted a series of experiments to investigate the morphological and physiological performances of cotyledons under salt stress at different developmental stages. The results showed that the cotyledons kept growing slowly to maintain the normal physiological activities of seedlings by balancing phytohormone levels, accumulating osmoprotectants and antioxidants, and scavenging reactive oxygen species (ROS). Salt stress activated the expression of osmoprotectant-related genes and enhanced the accumulation of related primary metabolites. Furthermore, differentially expressed transcriptional profiles of the cotyledons were also analyzed by cDNA-AFLP to gain an understanding of cotyledons in response to development and salt stress, and the results revealed a progressive increase in the expression level of development-related genes, which accounted for a majority of the total tested TDFs. Meanwhile, key photosynthetic and important salt stress-related genes also actively responded. All these performances suggest that “big cotyledons” are experiencing a delayed but active developmental process, by which *S. aralocaspica* may survive the harsh condition of the seedling stage.

## Introduction

Seed germination and seedling development are the two most critical stages in the life cycle of a plant, can determine the establishment, persistence, and succession of plant population, and can even be significant in the maintenance of biodiversity (Kitajima and Fenner, 2000; Grime, 2001; Hanley et al., 2004). The plant seedling stage is defined as the beginning of time when a plant is able to perform photosynthesis and live individually with radicle growth and cotyledon development after seed germination (Fenner, 1987; Kitajima and Fenner, 2000), but the end of the seedling stage has not been conclusive yet. Cotyledons, as the most significant organ in the seedling stage, play a critical role in the establishment of a stronger plant (Hanley, 1998). Cotyledons provide various nutrients (fatty acids, carbohydrates, mineral nutrients, etc.) for seed germination and early seedling development (Lovell and Moore, 1971; Milberg and Lamont, 1997; Lamont and Groom, 2002; Shi et al., 2021). In a short period after seedling emergence, cotyledons can carry out photosynthesis immediately to provide energy for seedling growth (Xu et al., 2021), which is the principal source of nutrients before the euphylla appears. Since true leaves appear, with continuous exhaustion of minerals and reserves, as well as decline in photosynthetic capacity, most cotyledons gradually stay in wilting and become senescent (Keighley, 1984; Marek and Stewart, 1992). Generally, the cotyledons of most dicots can exist for 15–30 days (Hanley et al., 2004). However, although cotyledons remain healthy, most of the energy for seedling growth comes from the photosynthesis of true leaves (Ampofo et al., 1976a,b). So far, very limited reports have focused on cotyledon function and the effect of its damage or excision on seedling growth (Meyer, 1998; Hanley and May, 2006; Zhang et al., 2011; Zheng et al., 2011), and documentation on desert plant species is even fewer. Ruffino et al. (2010) preliminarily demonstrated that cotyledons of *Chenopodium quinoa* seedlings significantly contribute to the absorption of excess ions and accumulation of osmotic protectants under salt stress, which may enhance the adaptability of seedlings to heterogeneous habitats.

*Suaeda aralocaspica* (Bunge) Freitag and Schütze (Amaranthaceae) is an annual halophyte with a single-cell (SC) C_4_ photosynthesis pathway (Voznesenskaya et al., 2001) and distributed in the inland cold desert of Junggar Basin in China (Commissione Redactorum Florae Xinjiangensis, 1994). It can produce heteromorphic seeds, which have different dormancy properties and present different responses to salt stress (Wang et al., 2008), and has evolved strong adaptability in arid, saline-alkali, and barren environments (Cao et al., 2015). After many years of field investigation, we found that seedlings of *S. aralocaspica* developed very slowly in early spring, i.e., they grew for nearly 2 months with only two cotyledons in an extremely saline habitat, and the cotyledons could remain on the seedlings and become much larger without visible true leaves and even preserved on an adult plant for the rest of its life. However, the detailed function of a “big” cotyledon in normal growth and response to adverse stress remains largely unknown. Apparently, this unique developmental mode of *S. aralocaspica* in early stages must have its own internal mechanisms; further understanding of which should be important for us to elucidate how early seedlings adapt to the heterogeneous habitats in the desert.

Transcriptional profiling is a useful technique to determine key genes related to development and stress responses (Parmar et al., 2017; Fatemi et al., 2019). cDNA-amplified fragment length polymorphism (cDNA-AFLP) is one of the convenient and effective strategies for transcriptome analysis, which costs much lower with observable gel polymorphism in analyzing differential gene expression, even without any prerequisite knowledge of sequences at the molecular level (Bachem et al., 1996; Zhou et al., 2013; Xue et al., 2015). It is also efficient in increasing the resolution of detection of expression levels using a smaller amount of mRNA (Reijans et al., 2003; Amini et al., 2017), carrying out comprehensive and systematic analyses of transcriptome data (Gupta et al., 2012; Xue et al., 2014), construction of transcriptional profile (Cnudde et al., 2003; Sarosh and Meijer, 2007), and separation of differentially expressed genes (Wu et al., 2013; Lang et al., 2014) with high reproducibility, accuracy, reliability, and efficiency (Jayaraman et al., 2008; Li et al., 2015). The sensitivity and specificity of this method allows for detecting even the poorly expressed genes and distinguishing between homologous sequences (Breyne et al., 2003). Recently, an increasing number of reports on molecular mechanisms in response to stress was illustrated based on the cDNA-AFLP technique, and large amounts of stress-inducible genes have been identified (Urbanavičiute et al., 2021), e.g., genes encoding proteins in plant cell protection such as *LEA* (late embryogenesis abundant protein) (Jin et al., 2019), *SOS1* (Na^+^/H^+^ antiporter protein) (Foster and Miklavcic, 2019), *P5CS* (pyrroline-5-carboxylate synthetase) (Székely et al., 2008), and *Cu/Zn-SOD* (Cu/Zn-superoxide dismutase) (Dvorák et al., 2021), etc.; genes encoding regulatory proteins in response to stress such as *bHLH* (basic helix-loop-helix transcription factor) (Wang et al., 2017), *CDPK* (calcium-dependent protein kinase) (Li et al., 2020), *PP2C* (protein phosphatase) (Shazadee et al., 2019), *CaM* (calmodulin) (Aldon et al., 2018), and *NCED* (9-*cis*-epoxycarotenoid dioxygenase) (Wang et al., 2021b), etc., which largely promote the understanding of the molecular biology of plants in development and response to stress. Nevertheless, the detailed knowledge of temporal alteration in the gene expression of halophyte seedlings is limited.

In this study, to clarify the function of cotyledons in seedling growth and response to stress in *S. aralocaspica*, we investigated morphological, physiological, and phytohormonal changes, and further analyzed transcriptome information (by cDNA-AFLP) and gene expression patterns during seedling (cotyledon) development, with which we expect to demonstrate the internal mechanism of the delayed development of seedlings of *S. aralocaspica* in adaptation to heterogeneous habitats.

## Materials and Methods

### Seedling Growth and Treatments

Mature seeds of *S. aralocaspica* were harvested from natural plants growing at Gurbantunggut Desert in Wujiaqu 103 regiment (44°29′N, 87°31′E; 430 mH) in October, in Xinjiang Uygur Autonomous Region, China. The seeds were air-dried and cleaned, and then stored at 4°C in sealed brown paper bags. Dimorphic (brown and black) seeds were separately sown in pots containing perlite: vermiculite (1:3, v/v) in a growth chamber and under conditions of a temperature regime of 20–25°C, 15–30% relative humidity, illumination of 150–700 μmol·m^−2^·s^−1^, and 14- to 16-h light/8- to 10-h dark photoperiod. The seedlings were cultivated with salt solutions containing 100, 300, or 500 mM NaCl [prepared with half-strength Hoagland solution (Arnon and Hoagland, 1940)] and applied at a 1-week interval using half-strength Hoagland solution only as the control. Cotyledons were harvested on days 3, 18, and 33 after emergence. Four biological replicates (in absence of salt stress) or three biological replicates (after salt treatment) of samples for physiological and gene expression analyses were collected from seedlings at different developmental stages, only 3- and 10-day-old seedlings were sampled under salt treatment for no enough seedlings left at 0 and 100 mM NaCl treatment thereafter. All the samples were immediately frozen in liquid nitrogen upon harvest and then stored at −80°C until use.

### Observation of Micro-Structure of Apical Growth Point in Development

Paraffin sections were prepared following Li et al. (2021) to visualize the development of the apical growth point of seedlings in *S. aralocaspica*. For fixation, an FAA solution was used to fix the tissue of the joint part between two cotyledons and the hypocotyl. For dehydration, tissues were treated in 50% ethanol (30 min, two changes), followed by 1% Safranin O solution (prepared in 70% ethanol) staining for 8–10 h at room temperature, dehydrated in a series of ethanol from 80% to absolute ethanol, and then cleared at different concentrations of xylene and absolute ethanol. First-grade paraffin powder and a small amount of xylene were added to the tissue container in an oven at 38°C overnight. For paraffin inclusion and embedding, the above tissues were incubated at 56°C until the wax was completely melted, transferred into melted second-grade paraffin for 1 h, and then submerged in third-grade paraffin for 3 h. Finally, the melted paraffin containing tissues was poured into a paper tank. For sectioning, paraffin blocks were sliced into 6- to 12-μm sections with a Leica RM2126 microtome (Leica Biosystems, Germany). For deparaffinization and staining, glass slides with sections were successively incubated in xylene, xylene + ethanol, and ethanol, followed by 1% Safranin O staining overnight. Then, the slides were quickly and gently dipped into 1% Fast Green solution for about 10 s and immediately transferred into a series of ethanol, ethanol + xylene, and xylene. After mounting, the sections were inspected under a Leica DM3000 light microscope (Leica Microsystems, Germany) and photographed using the LAS V4.0 program.

### Determination of Phytohormones

Seedlings were subjected to different concentrations of NaCl (0, 100, and 500 mM) treatment, and cotyledons were sampled at 0 (dry seeds), 7, 14, 21, 28, and 35 days after emergence. Four replicates of each treatment were homogenized in liquid nitrogen and transferred into 10 ml deionized distilled H_2_O (ddH_2_O) at 30°C for 3–4 h to extract phytohormones. High performance liquid chromatography (HPLC) (SCL-10AVP, Shimadzu, Japan) was conducted to determine four endogenous hormones: zeatin (ZT), gibberellin acid 3 (GA_3_), indole-3-acetic acid (IAA), and abscisic acid (ABA) (Pan et al., 2010). The standard curves, regression equation, and relationship coefficient were generated from standard solutions of ZT, GA_3_, IAA, ABA, and a series of dilutions (Supplementary Table 1). Conditions for chromatography were: (A) mobile phase: methanol: glacial acetic acid (0.75% in H_2_O) = 45:55; (B) flow rate: 0.7 ml·min^−1^; (C) column temperature: 35°C; (D) detection wavelength: 245 nm.

### Measurement of Physiological Parameters

#### Determination of Tissue Water Content, and Na^+^ and K^+^ Concentrations

Fresh seedlings were weighed immediately to record the fresh weight (FW) and deactivated at 150°C for 20 min; then, they were transferred to an oven at 60°C until a constant dry weight (DW) was achieved. Tissue water content (TWC) was calculated as follows: TWC (%) = (FW–DW) × 100/FW (Tang et al., 2019). Fresh seedlings (1.5 g) were treated as above until DW was obtained. A dry plant material (0.3 g) was dissolved in HNO_3_, and a total volume of 25 ml was prepared by the addition of ddH_2_O. Na^+^ and K^+^ concentrations were measured by inductively coupled plasma atomic emission spectrometry (Agilent AA240, United States). HNO_3_ was used as the control (Al-Ashkar et al., 2021).

#### Determination of Concentration of Osmotic Protectants

For proline, seedlings (0.15 g) were homogenized in aqueous sulfosalicylic acid [5 ml, 3% (w/v)] and boiled for 10 min. The extract (200 μl), combined with a mixture containing acidic ninhydrin [300 μl, 2.5% (w/v)] and glacial acetic acid (200 μl), was incubated in boiling water bath for 40 min and then cooled on ice. After adding toluene (500 μl), the solution was shaken vigorously and the absorbance of the supernatant (200 μl) was measured at 520 nm. Toluene was used as the blank. L-Proline (0–20 μg·ml^−1^) was used to prepare the standard curve (Khan et al., 2017). For soluble sugar (SS), seedlings (0.12 g) were sheared into pieces and boiled in distilled H_2_O (dH_2_O, 10 ml) for 30 min. The extract (50 μl) was added to dH_2_O (150 μl), reacted with anthrone ethyl acetate [50 μl, 2% (w/v)] and concentrated sulfuric acid (500 μl), and then quickly boiled for 1 min. The absorbance at 630 nm was detected, and a standard curve (0–100 μg·ml^−1^ of glucose) was prepared to calculate SS content (Cui et al., 2018). For glycine betaine (GB), seedlings (0.15 g) were homogenized in liquid nitrogen and transferred into dH_2_O (1 ml) in a microtube, and then centrifuged at 10,000 g for 15 min; the supernatant (300 μl) mixed with saturated Reinecke's salt (500 μl) was incubated on ice for 1 h, followed by centrifuging at 10,000 g for 15 min. Finally, the precipitate was dissolved in acetone (70%). A calibration curve (0–200 μg·ml^−1^ of GB) was used to determine the concentration, and the absorbance was read at 525 nm (Zhao et al., 2016).

#### Measurement of ${\text{O}}_{2}^{-}$ Production, and H_2_O_2_ and Malondialdehyde Concentrations

For ${\text{O}}_{2}^{-}$ production, seedlings (0.15 g) were homogenized with phosphate-buffered saline (PBS, 1.3 ml, 50 mM, pH 7.8), and the slurry was centrifuged at 10,000 g and 4°C for 20 min. The mixture containing the supernatant (1 ml) and hydroxylamine (1 ml, 1 mM) was incubated at 25°C for 1 h, and then was added α-naphthalenamine (1 ml, 7 mM) and 4-aminobenzenesulfonic acid (1 ml, 17 mM). The reaction mixture (500 μl) was incubated at 25°C, and then was eliminated pigments with ether. A standard curve (0–50 μM of NaNO_2_) was prepared, and the absorbance was recorded at 530 nm (Imran et al., 2021). For H_2_O_2_ concentration, seedlings (0.15 g) were homogenized with acetone (2 ml) and then centrifuged at 3,000 g and 4°C for 10 min. The supernatant (1 ml) was added into a mixture of titanium sulfate [100 μl, 5% (w/v)] and ammonia (200 μl), and the precipitate that formed was washed to remove pigments and then dissolved in sulfuric acid (5 ml, 2 M). The absorbance was measured at 415 nm, and a calibration curve (0–100 μM of H_2_O_2_) was prepared to determine the concentration (Wang et al., 2021a). For malondialdehyde (MDA) concentration, seedlings (0.12 g) were ground into homogenate with trichloroacetic acid [TCA, 1 ml, 10% (w/v)] and centrifuged at 4,000 g for 10 min. The supernatant (300 μl) was mixed with thiobarbituric acid [TBA, 0.5% (w/v)] and then centrifuged. The absorbance of the supernatant was monitored separately at 450, 532, and 600 nm. The concentration of MDA was calculated using the equation (*C*) = 6.45 × (D_532_ – D_600_) – 0.56 × D_450_, where D_450_, D_532_, and D_600_ stand for absorbance values at 450, 532, and 600 nm, respectively (Motmainna et al., 2021).

#### Determination of the Content of Non-Enzymatic Antioxidants

Seedlings (0.15 g) were homogenized with TCA [1 ml, 5% (w/v)] and then centrifuged. For AsA content, the supernatant was mixed with TCA (200 μl, 612 mM), NaH_2_PO_4_ (100 μl, 150 mM), FeCl_3_ (100 μl, 184.9 mM), H_3_PO_4_ (200 μl, 4.49 M), and 2,2-biphenyl (200 μl, 214.8 mM) and then incubated. The absorbance value was recorded at 525 nm, and a standard curve (0–70 mM AsA) was plotted to calculate the AsA content (Rabelo et al., 2020). For GSH content, the supernatant was mixed with 5,5-dithio-bis (2-nitrobenzoic acid) (DTNB, 90 μl) and NaH_2_PO_4_ (130 μl, 150 mM), and then incubated. The absorbance value was determined at 412 nm, and the GSH content was calculated from a standard curve of GSH (0–0.12 mM) (Castro et al., 2021).

#### Measurement of the Activity of Antioxidant Enzymes

Seedlings (0.2 g) were homogenized and added into a PBS buffer (1.3 ml, 50 mM, pH 7.8). After centrifugation, the supernatant was used as crude enzyme for the analysis of different enzymatic activities immediately. For superoxide dismutase (SOD), a reaction mixture (3 ml) consisting of crude enzyme extract (50 μl), PBS (50 mM, pH 7.8), EDTA-Na_2_ (100 μM), methionine (130 mM), nitroblue tetrazolium (NBT, 750 μM), and riboflavin (2 μM) was incubated at 25°C for 25 min under a light source of 50 μmol·m^−2^·s^−1^, and then was measured the absorbance at 560 nm. One unit (U) of SOD activity was defined as the amount of enzyme for 50% inhibition of NBT photoreduction rate (Chrysargyris et al., 2018). For peroxidase (POD), a reaction mixture (3 ml) containing the crude enzyme extract (50 μl), PBS (1 ml, pH 7.0), H_2_O_2_ [1 ml, 0.3% (v/v)], and guaiacol [0.95 ml, 0.2% (v/v)] was prepared, the absorbance was recorded at 470 nm, and the activity was determined from the oxidation of guaiacol (Abid et al., 2018). For catalase (CAT), the reaction mixture (3 ml) contained the crude enzyme extract (100 μl), H_2_O_2_ [1 ml, 0.3% (v/v)], and H_2_O (1.9 ml). The activity was determined by measuring the decrease in H_2_O_2_ absorbance at 240 nm (Gao et al., 2019). The absorbance values of POD and CAT mixtures were recorded with a spectrophotometer (UV-3010; Shimadzu, Japan) within 1 min against a blank without the crude enzyme. The concentration of total proteins was assessed at 595 nm with the method of Bradford (1976). All the enzyme activities were expressed as unit per mg total protein.

#### Determination of Chlorophyll and Carotenoid Concentrations

Seedlings (0.15 g) were treated with 96% ethanol, and the resulting homogenate was centrifuged at 4,000 g for 10 min. The absorbance of the supernatant (300 μl) was measured at 470, 649, and 665 nm. The concentrations of chlorophylls (Chl_a_ and Chl_b_) and carotenoid (Car) were calculated as Chl_a_ = 13.95 D_665_ − 6.88D_649_, Chl_b_ = 24.96 D_649_ − 7.32 D_665_, and Car = (1,000 D_470_ − 2.05 Chl_a_ − 114.8Chl_b_)/245, respectively. Total chlorophyll concentration was calculated by the addition of Chl_a_ and Chl_b_ (Gao et al., 2021).

#### Estimation of the Activity of Key Photosynthetic Enzymes

Seedlings (0.4 g) were homogenized in liquid nitrogen with an extraction buffer (1.5 ml) containing Tris-H_2_SO_4_ (100 mM, pH 8.2), EDTA (1 mM), β-mercaptoethanol (7 mM), and glycerol [5%(v/v)], and centrifuged at 4,500 g and 4°C for 20 min. The supernatant was used as crude enzyme for assay of the activity of phosphoenolpyruvate carboxylase (PEPC) immediately. A reaction mixture (1 ml) containing NaHCO_3_ (143 μl, 70 mM), MgSO_4_ (143 μl, 70 mM), phosphoenolpyruvate (PEP) (286 μl, 14 mM), and NADH (429 μl, 5 mM) was incubated at 30°C for 30 min. After the addition of malate dehydrogenase (4 μl) and the crude enzyme (40 μl) to initiate the reaction, the mixture was recorded with an absorbance value for 3 min at 340 nm. One unit (U) of PEPC activity was defined as 1 nmol of NADH oxidation per minute per mg protein (You et al., 2020).

### RNA Extraction

Total RNA was isolated from 0.1 g cotyledons of *S. aralocaspica* according to the manufacturer's instructions in the RNAprep Pure Plant Kit (DP432; Tiangen, Beijing, China). Cotyledons were sampled from seedlings derived from dimorphic seeds (brown and black seeds) at different developmental stages (3, 18, and 33 days after emergence) or at different NaCl concentrations (0, 100, 300, and 500 mM). The quantity and quality of total RNA were determined with a Nanodrop ND-1000 UV spectrophotometer (Thermo Fisher Scientific, Waltham, MA, United States).

### Analysis of cDNA-AFLP

About 1 μg of mRNA [Poly(A) mRNA Purification Kit, SK8810; Sangon, Shanghai, China] was reversely transcribed into double-stranded cDNA using a PrimeScript™ Double Strand cDNA Synthesis kit (6111A; TaKaRa, Dalian, China). The cDNA (500 ng) was digested with *Eco*RI and *Mse*I (P0525S and R0101V; NEB, Ipswich, MA, United States), and was then precipitated, resuspended, and ready for ligation with adaptors using a T4 DNA ligase (NEB, Ipswich, MA, United States).

Non-selective PCR pre-amplification was performed by mixing the above ligated products and non-selective primers *Eco*RI or *Mse*I (Supplementary Table 2) under conditions of 94°C and 4 min for denaturation, then 30 cycles at 94°C for 30 s, 56°C for 30 s, and 72°C for 1 min, with a final 7 min extension at 72°C. PCR products were used as templates for subsequent selective amplification (SA) under the same conditions. The SA involved touchdown PCR, with the annealing temperature starting from 65 to 56°C, followed by 25 cycles of annealing temperature at 56°C. The AFLP protocol was the same as described by Banikamali et al. (2020), and a total of 25 selective primer combinations (i.e., 5 primers with *Eco*RI site and 5 primers with *Mse*I site) were used for transcript profiling (Supplementary Table 2).

SA products (i.e., transcript-derived fragments, TDFs) were mixed with 0.5 times the volume of a denaturing buffer as described by Creste et al. (2001), and were separated on 6% polyacrylamide sequencing-type gel running at 850 V for 3–4 h after a 30-min pre-run. Gels were visualized by silver staining (with AgNO_3_) (Amini et al., 2017) and dried for scanning of images.

### Isolation and Sequencing of TDFs

Differentially displayed polymorphic TDFs in silver-stained gels were identified and eluted according to the procedure suggested by Baisakh et al. (2006), and then re-amplified with appropriately selected primer combinations. Specific bands were sequenced after being recovered from the gels and purified using TIANgen Midi Gel Purification Kit (DP209; Tiangen, Beijing, China). Sequences of TDFs were analyzed for their homology against the publicly available genomic database of *S. aralocaspica* (Wang et al., 2019a) and non-redundant genes/ESTs/transcripts in the NCBI database (http://www.ncbi.nlm.nih.gov/BLAST) using the BLASTN and BLASTX algorithms. Functional classification of selected genes was performed using the MIPS database (http://mips.gsf.de/proj/funcatDB/search_main_frame.html).

### Quantitative Real-Time PCR

qRT-PCR was performed with target TDFs and some primary metabolite-related genes to investigate the expression pattern in seedlings of *S. aralocaspica* at different developmental stages or under NaCl treatment. Each reversely transcriptional reaction was performed with total RNA (1 μg) in a final volume of 20 μl using the M-MLV RTase cDNA Synthesis Kit (D6130l TaKaRa, Dalian, China). Gene-specific primers of target TDFs and primary metabolite-related genes were designed using the Primer-Blast tool (Supplementary Table 3), and the β*-actin* of *S. aralocaspica* was used as internal reference (Cao et al., 2016). The relative expression level of target genes was quantified according to the mathematical model *R*=2^−Δ*ΔCT*^ (Shi and Chiang, 2005), where ΔΔCT = ΔCT_target sample_ − ΔCT_control sample_, and ΔCT_sample_= CT_test gene_ − CT_reference gene_. The final value of the relative quantification was described as a fold change of gene expression in the test sample compared to the control. Data are presented as mean ± standard error of four biological replicates and two technical replicates (*n* = 8).

### Statistical Analysis

All data were plotted with the software of GraphPad Prism Version 7.0 for Windows (GraphPad Software, San Diego, United States) and analyzed using SPSS Version 26.0 for Windows (SPSS Inc., Chicago, United States). One way ANOVA and two-way ANOVA were conducted to test the significance of different treatments, and Tukey's HSD test was performed for multiple comparisons to determine significant differences between samples at 0.05, 0.01, 0.001, and 0.0001 significance levels. When the homogeneity of variance assumption was not met, differences were analyzed by Welch's ANOVA and Games Howell *post-hoc* test (McDonald, 2014).

## Results

### The Unique Morphology of the Seedlings in Development

**Figure 1:**
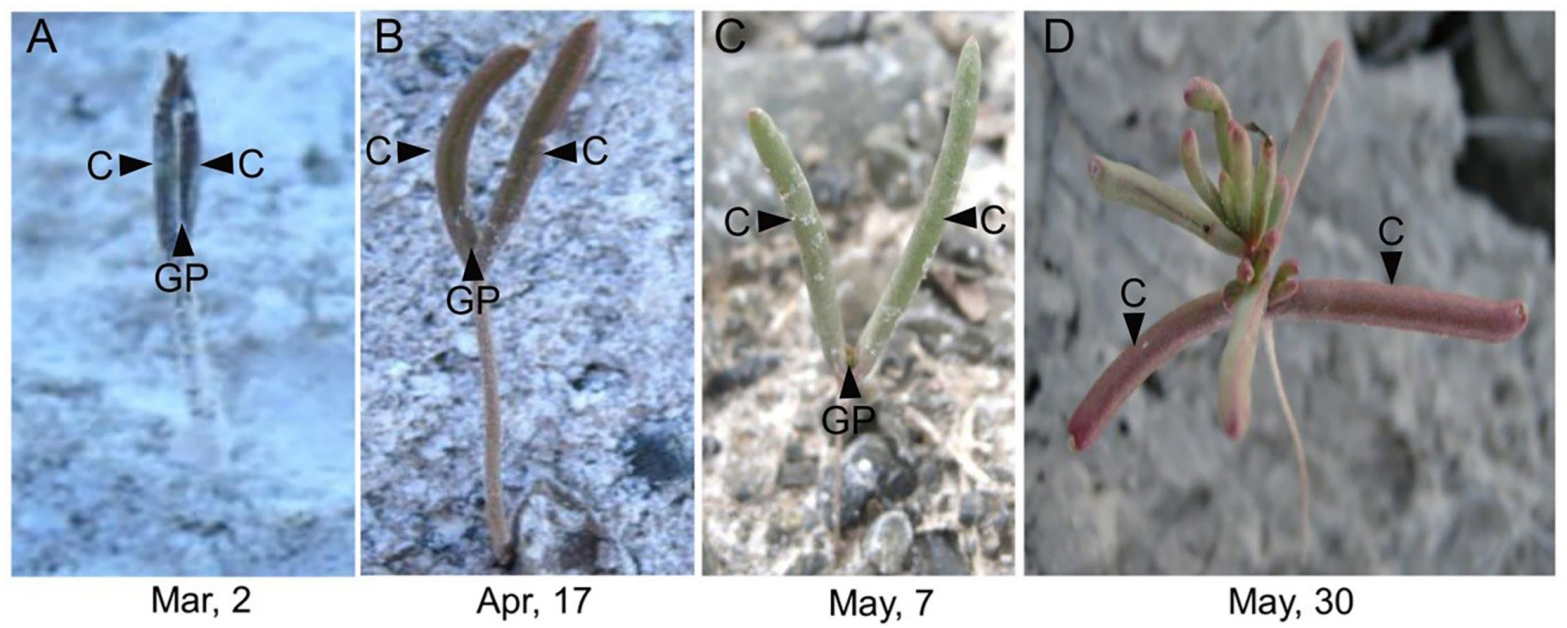
Unique phenotype of “big cotyledons” of *Suaeda aralocaspica* in the natural habitat. C, cotyledon; GP, growth point; Mar, March; Apr, April. **(A)** Early-stage germinating seedling, **(B)** one-month-old seedling, **(C)** 2-month-old seedling, and **(D)** 3-month-old seedling.

**Figure 2:**
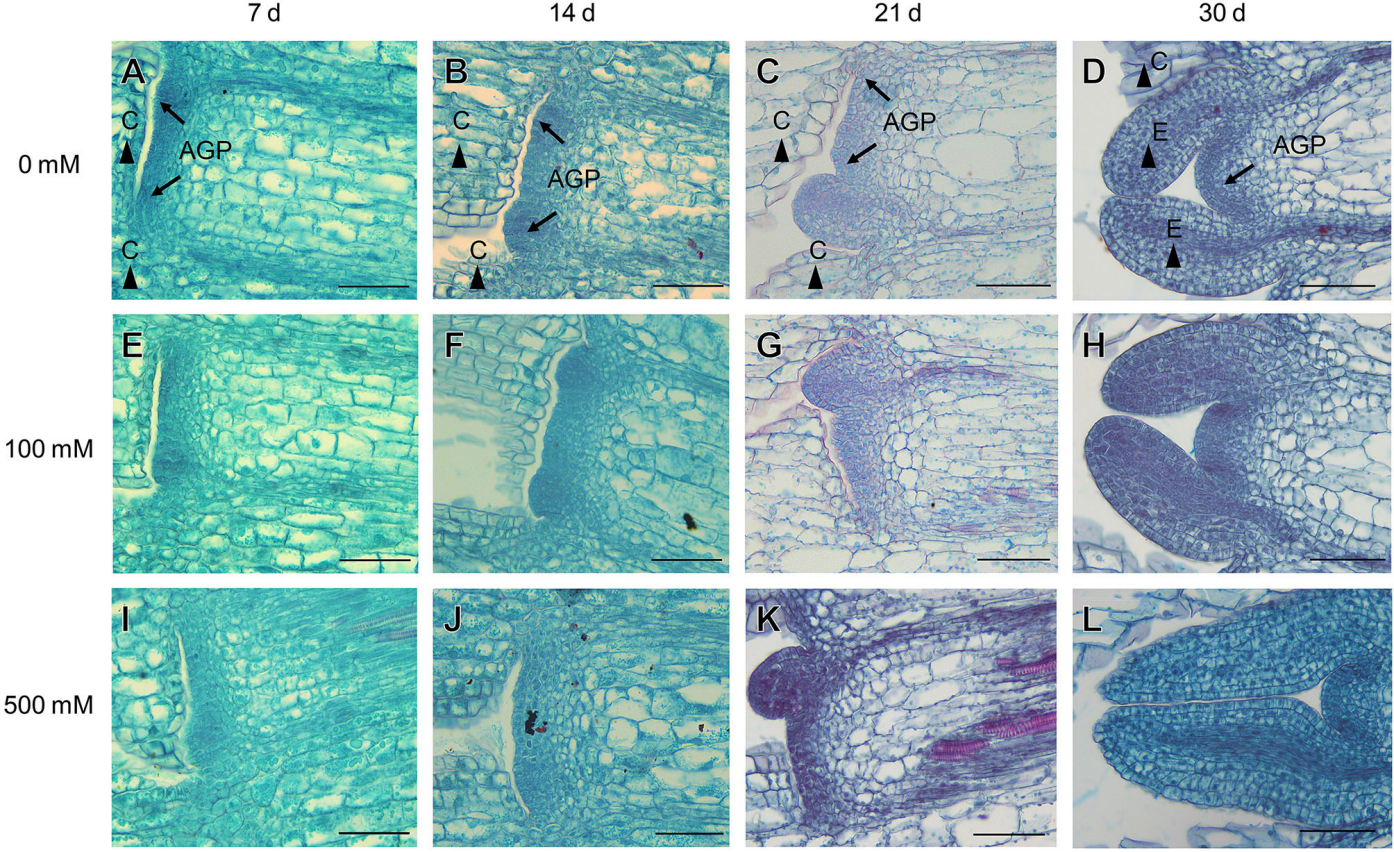
Anatomic structures of the apical growth point (AGP), cotyledon (C), and euphylla (E) of *S. aralocaspica* after seedling emergence under different salt stress conditions. **(A–D)** without NaCl treatment; **(E–H)** 100 mM NaCl; **(I–L)** 500 mM NaCl. 7 d, 14 d, 21 d, 30 d: represent different developmental days. Scale bar is 50 μm.

In the natural habitat, *S. aralocaspica* seeds germinate at the beginning of March, and seedlings are gradually established from March to May. For extremely saline conditions, the seedlings may stay with only two cotyledons for nearly 2 months, presenting significant developmental retardation (Figure 1). Before true leaves start to visibly emerge, the two cotyledons were able to enlarge gradually to a much larger size compared with that of the seedling at emergence. Observation of the morphological change in seedling apical growth point (AGP) under a microscope revealed that the first leaf bud was visible on the 21st day, and appeared to grow faster at a higher salt concentration, i.e., 500 mM NaCl treatment promoted the generation of larger leaf primordium compared with the control (Figure 2). Such a phenomenon suggests that “large” cotyledons may be an adaptation strategy for early seedlings to get through harsh conditions.

### Physiological Responses of the Seedlings at Different Developmental Stages

#### Changes in Phytohormones

**Figure 3:**
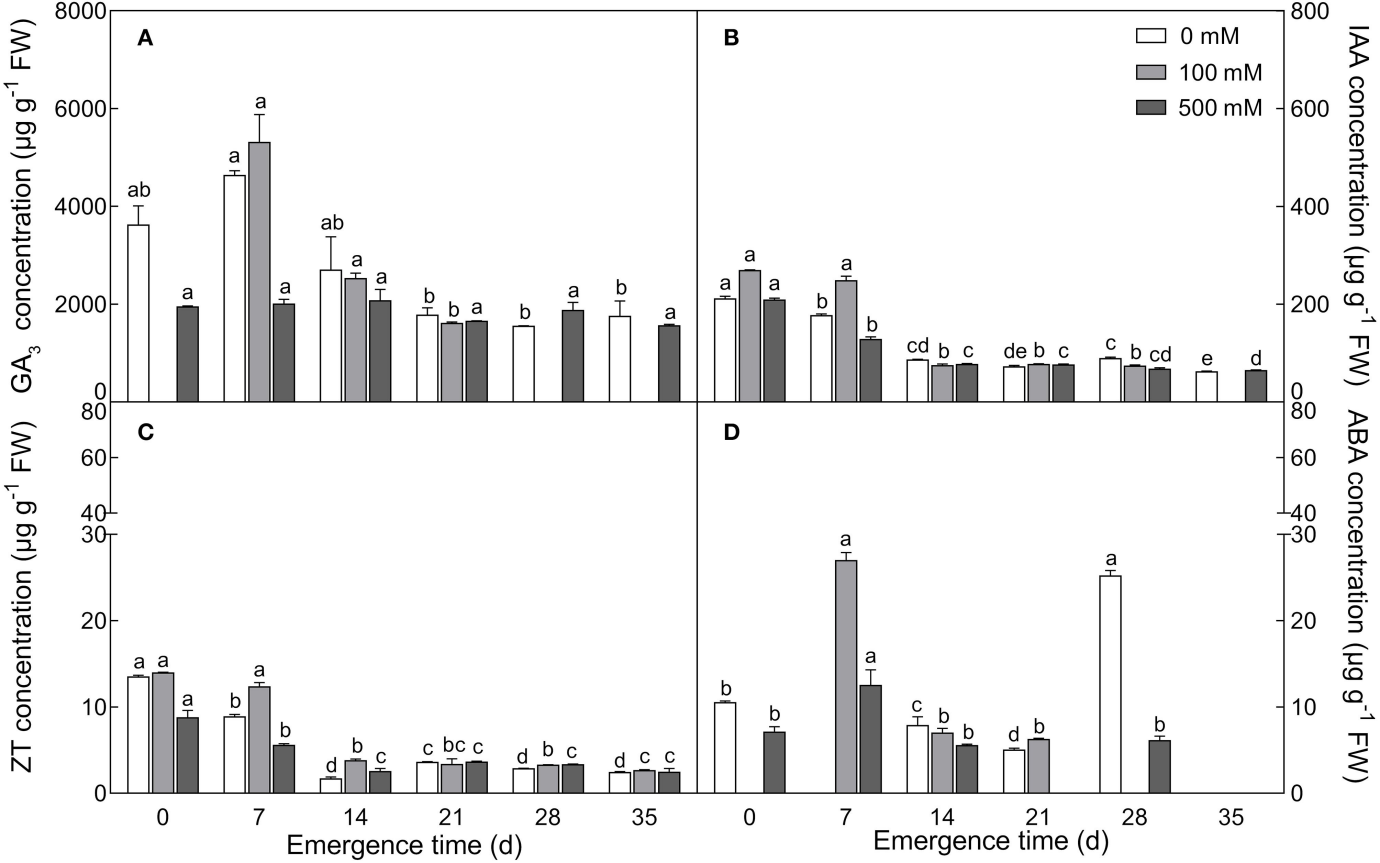
Changes in the content of four endogenous phytohormones during seedling development under salt treatment. **(A)** GA_3_; **(B)** IAA; **(C)** ZT; **(D)** ABA. Different letters in lowercase indicate a significant difference (*P* < 0.05) of the same salt concentration at different developmental stages. Values are means ± SE of three replicates.

Four endogenous phytohormones, GA_3_, IAA, ZT and ABA, showed a general trend in developmental seedlings (0–35 days), in which relatively higher levels of the four hormones were observed in 7-day-old seedlings, followed by significant decreases in 14-day-old seedlings, and then were maintained relatively stable thereafter from 14 to 35 days, except for ABA on the 28th day (Figure 3). Among them, GA_3_ presented the highest level, IAA was moderate, ZT and ABA were relatively lower, and ABA content fluctuated slightly. Under salt treatment, lower concentration (100 mM) of NaCl stimulated the increase, while higher concentration (500 mM) resulted in decrease in the content of the four hormones, especially in seedlings younger than 14 days. Our results suggest that there are no significant fluctuations in GA_3_, IAA, and ZT contents during the later stage (14–35 days) of seedling development, at least in our experiment.

#### Changes in Inorganic and Organic Osmolytes

**Figure 4:**
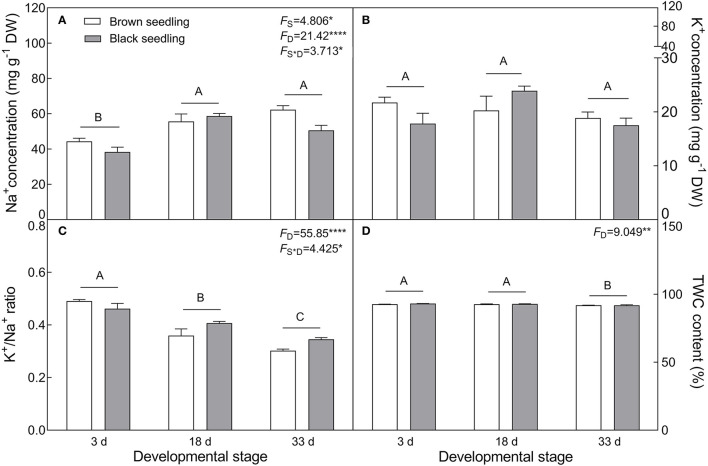
Changes in Na^+^, K^+^ and tissue water content (TWC) during seedling development. **(A)** Na^+^; **(B)** K^+^; **(C)** K^+^/Na^+^ ratio; **(D)** TWC. 3 d, 18 d, and 33 d: represent 3, 18, 33 days after seedling emergence, respectively. *F*-values are given when significance levels are reached (the subscript “D” represents “development”; “S”, “seed type”; *P < 0.05, **P < 0.01; ****P < 0.0001). Bars with different uppercase letters indicate significant differences (*P* < 0.05) according to Tukey's test. Values are means ± SE of four replicates. The Y-axis scale of TWC was adjusted to 150% to make visible of the difference comparison.

**Figure 5:**
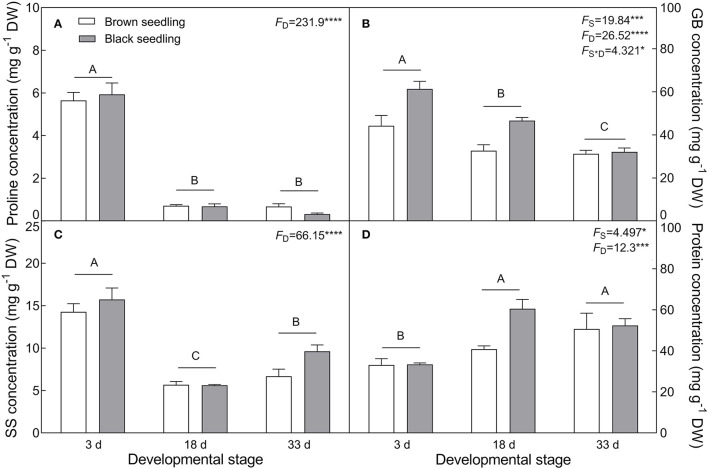
Changes in the concentration of osmotic protectants during seedling development. **(A)** Proline, **(B)** glycinbetaine (GB), **(C)** soluble sugar (SS), and **(D)** total soluble protein. 3 d, 18 d, and 33 d: represent 3, 18, and 33 days after seedling emergence, respectively. *F*-values are given when significance levels are reached (the subscript “D” represents “development”; “S”, “seed type”; *P < 0.05, ***P < 0.001; ****P < 0.0001). Bars with different uppercase letters indicate significant differences (*P* < 0.05) according to Tukey's test. Values are means ± SE of four replicates.

The seedlings showed a significant accumulation of Na^+^ in cotyledons with the development progression (*F*_2, 18_ = 21.42, *P* < 0.0001), which differed between seedlings derived from two seed morphs (*F*_1, 18_ = 4.806, *P* = 0.0417) (Figure 4A); the level of K^+^ decreased in the brown seedlings from days 3 to 33 after emergence but increased in the black seedlings on day 18, and no significant change was present between days 3 and 33 in the black seedlings (Figure 4B). As a consequence, K^+^/Na^+^ ratios in both types of seedlings were significantly reduced (*F*_2, 18_ = 55.85, *P* < 0.0001) (Figure 4C). The TWC was also decreased by the developmental stage extension (*F*_2, 18_ = 9.049, *P* = 0.0019) (Figure 4D). A significant decrease in proline (*F*_2, 18_ = 231.9, *P* < 0.0001), GB (*F*_2, 18_ = 26.52, *P* < 0.0001), and SS (*F*_2, 18_ = 66.15, *P* < 0.0001) contents was observed in the two types of seedlings, especially proline content, which was reduced by about 9 folds in the brown and 18 folds in the black seedlings on day 33 (Figures 5A–C); whereas the total soluble protein was increased significantly from days 3 to 33 in both types of seedlings (*F*_2, 18_ = 12.3, *P* < 0.001) (Figure 5D). Between the different seedlings, the contents of GB (*F*_1, 18_ = 19.84, *P* < 0.001) and total soluble protein (*F*_1, 18_ = 4.497, *P* = 0.0481) were significantly higher in the black seedlings than that of the brown seedlings.

#### Levels of the Reactive Oxygen Species and Membrane Lipid Peroxidation

**Figure 6:**
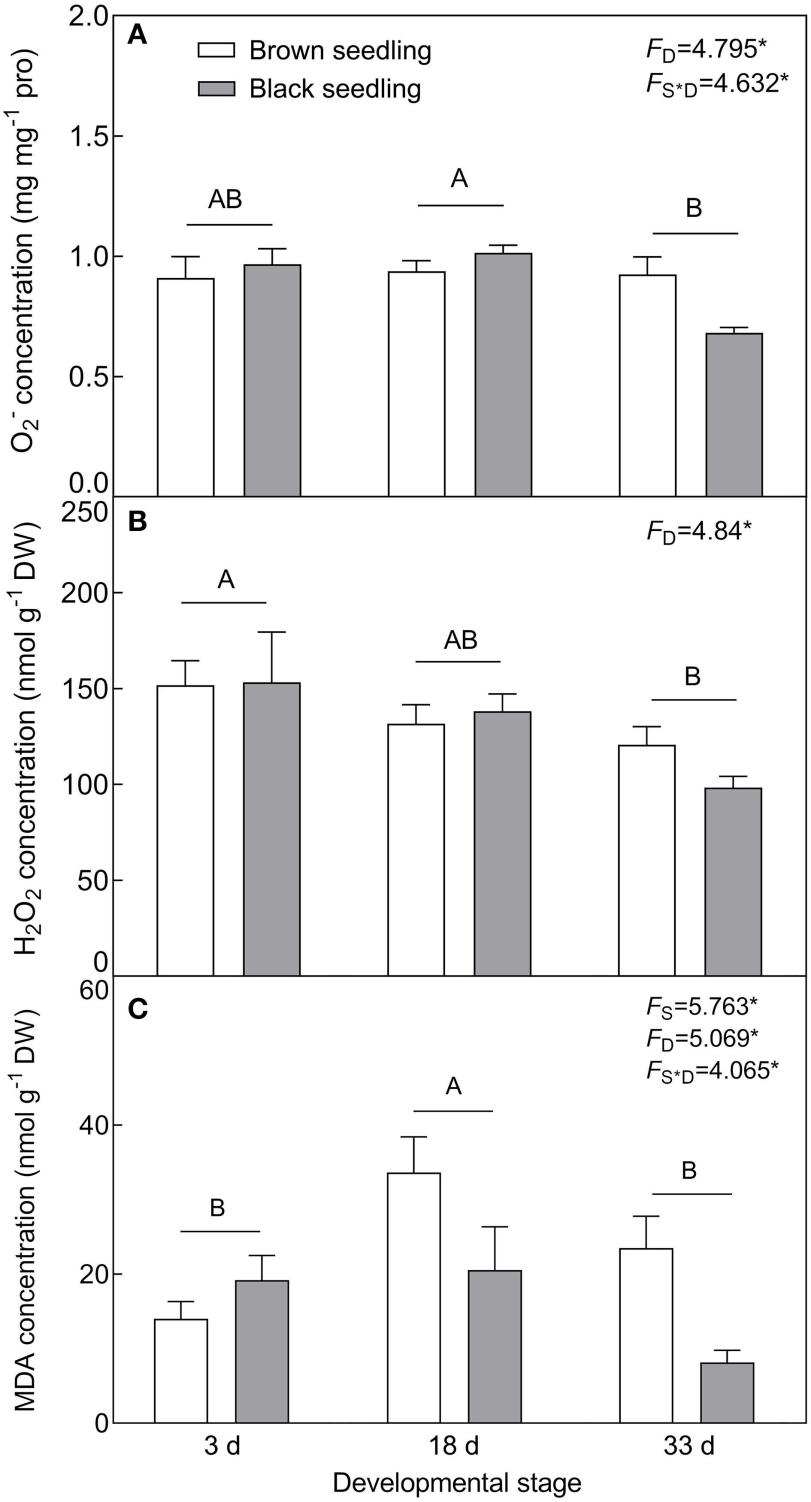
Changes in the ROS level and MDA concentration during seedling development. **(A)** ${\text{O}}_{2}^{-}$, **(B)** H_2_O_2_, and **(C)** MDA. 3 d, 18 d, and 33 d: represent 3, 18, and 33 days after seedling emergence, respectively. *F*-values are given when significance levels are reached (the subscript “D” represents “development”; “S”, “seed type”; *P < 0.05). Bars with different uppercase letters indicate significant differences (*P* < 0.05) according to Tukey's test. Values are means ± SE of four replicates.

With the development progression of cotyledons, the production of ${\text{O}}_{2}^{-}$ (*F*_2, 18_ = 4.795, *P* = 0.0214) and H_2_O_2_ (*F*_2, 18_ = 4.84, *P* = 0.0208) was gradually decreased (Figures 6A,B). MDA concentration was distinctly accumulated on day 18 (*F*_2, 18_ = 5.069, *P* = 0.0179) and significantly higher in the brown seedlings than that of the black seedlings (*F*_1, 18_ = 5.763, *P* = 0.0274) (Figure 6C). H_2_O_2_ accumulation had no significant difference between the two types of seedlings (*F*_1, 18_ = 0.1768, *P* = 0.6791); whereas ${\text{O}}_{2}^{-}$ level (*F*_2, 18_ = 4.632, *P* = 0.0238) and MDA concentration (*F*_2, 18_ = 4.065, *P* = 0.0349) were significantly affected by the interaction of seedling type and developmental stage. Our results suggest that no stress significantly disturbs the normal growth of the two types of seedlings.

#### Responses of Non-Enzymatic Antioxidants and Antioxidant Enzymes

**Figure 7:**
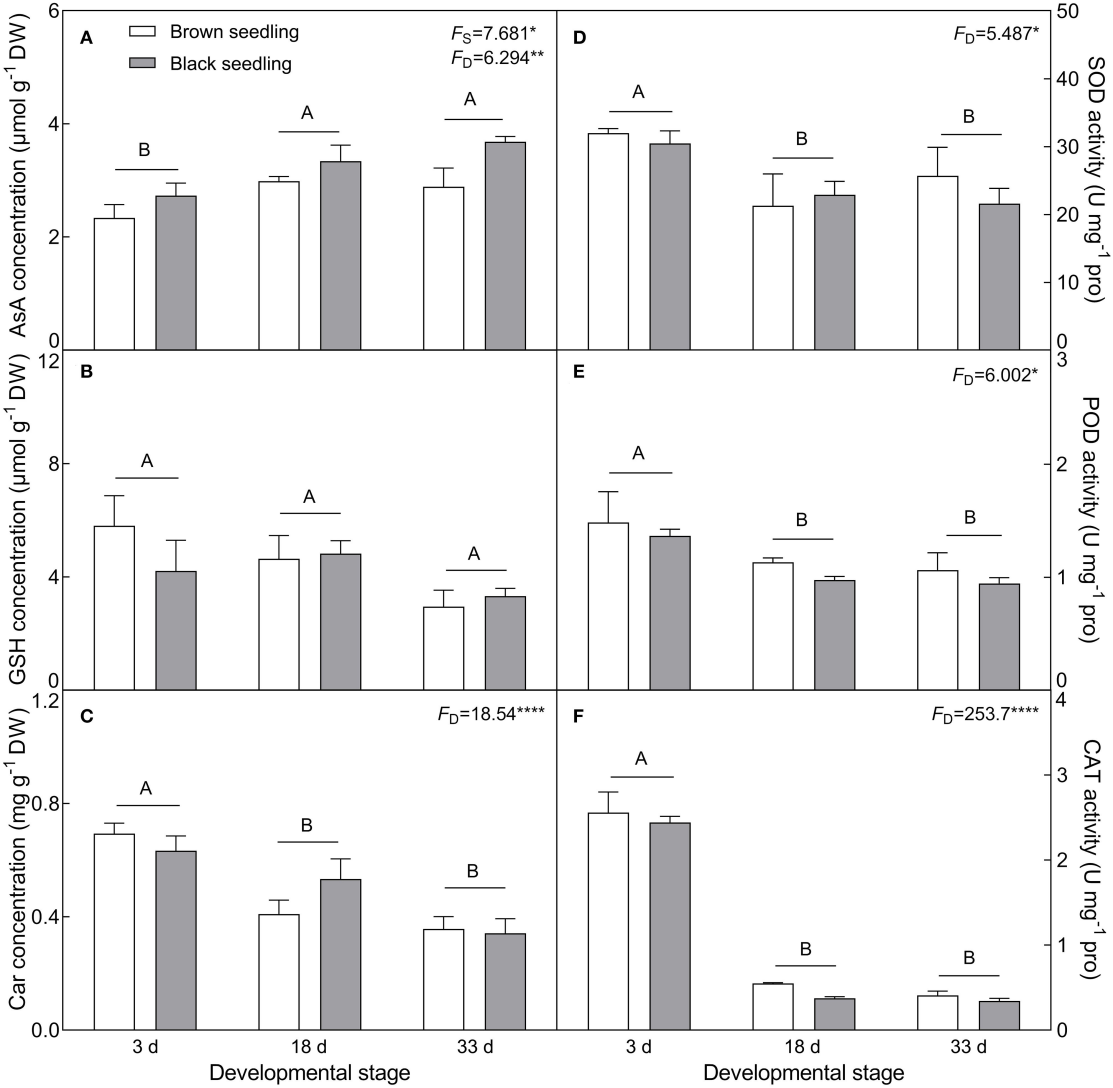
Changes in the concentration of non-enzymatic antioxidants and activity of antioxidant enzymes during seedling development. **(A)** AsA, **(B)** GSH, **(C)** Car, **(D)** SOD, **(E)** POD, and **(F)** CAT. 3 d, 18 d, and 33 d represent 3, 18, and 33 days after seedling emergence, respectively. *F*-values are given when significance levels are reached (the subscript “D” represents “development”; “S”, “seed type”; *P < 0.05; **P < 0.01; ****P < 0.0001). Bars with different uppercase letters indicate significant differences (*P* < 0.05) according to Tukey's test. Values are means ± SE of four replicates.

The concentration of ASA (*F*_2, 18_ = 6.294, *P* = 0.0085) showed an increasing tendency with the development progression, and was significantly higher in the black seedlings than that of the brown seedlings (*F*_1, 18_ = 7.681, *P* = 0.0126) (Figure 7A). On the contrary, the contents of GSH and Car (*F*_2, 18_ = 18.54, *P* < 0.001) presented a decreasing trend (Figures 7B,C) and showed no significant difference between the two types of seedlings (*F*_1, 18_ = 0.3018, *P* = 0.5895 for GSH; *F*_1, 18_ = 0.1415, *P* = 0.7112 for Car) from days 3 to 33. The activities of SOD (*F*_2, 18_ = 5.487, *P* = 0.0138), POD (*F*_2, 18_ = 6.002, *P* = 0.0101) and CAT (*F*_2, 18_ = 253.7, *P* < 0.0001) were significantly reduced with the seedling development from days 3 to 33, especially that of CAT, but no significant difference was detected between the two types of seedlings (*F*_1, 18_ = 0.3135, *P* = 0.5825 for SOD; *F*_1, 18_ = 1.468, *P* = 0.2413 for POD; *F*_1, 18_ = 1.878, *P* = 0.1874 for CAT) (Figures 7D–F). Our data suggest that normal physiological activities are proceeding in seedling (cotyledon) development from days 3 to 33.

#### Changes in Chl Content and PEPC Activity

**Figure 8:**
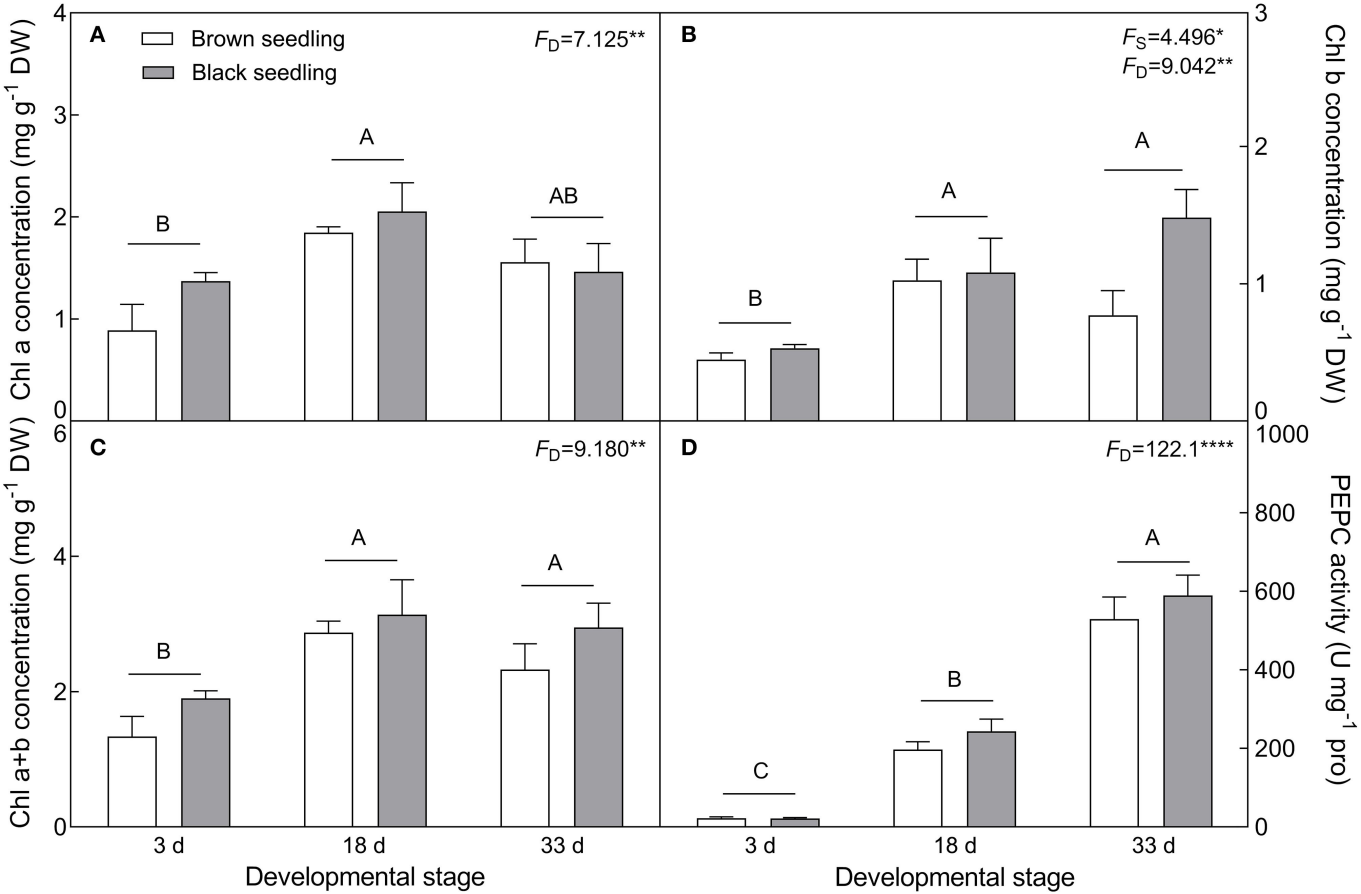
Changes in the concentration of chlorophyll and activity of PEPC during seedling development. **(A)** Chl a, **(B)** Chl b, **(C)** total Chl, and **(D)** PEPC. 3 d, 18 d, 33 d represent 3, 18, and 33 days after seedling emergence, respectively. *F*-values are given when significance levels are reached (the subscript “D” represents “development”; “S”, “seed type”; *P < 0.05; **P < 0.01; ****P < 0.0001). Bars with different uppercase letters indicate significant differences (*P* < 0.05) according to Tukey's test. Values are means ± SE of four replicates.

With the development progression from days 3 to 33, the contents of Chl a (*F*_2, 18_ = 7.125, *P* = 0.0053), Chl b (*F*_2, 18_ = 9.042, *P* = 0.0019), and total Chl (*F*_2, 18_ = 9.18, *P* = 0.0018) of both types of seedlings were significantly increased (Figures 8A–C). Corresponding to the increase in chlorophyll content, the activity of PEPC (*F*_2, 18_ = 122.1, *P* < 0.0001) was also significantly enhanced by seedling growth and achieved the highest value (about 24- and 27-fold higher than that of the control for the brown and black seedlings, respectively) on day 33 (Figure 8D). These results suggest that the photosynthetic function of the cotyledons is strengthened during seedling development.

### Responses of the Seedlings Under Salt Stress

#### Changes in the Primary Metabolites

**Figure 9:**
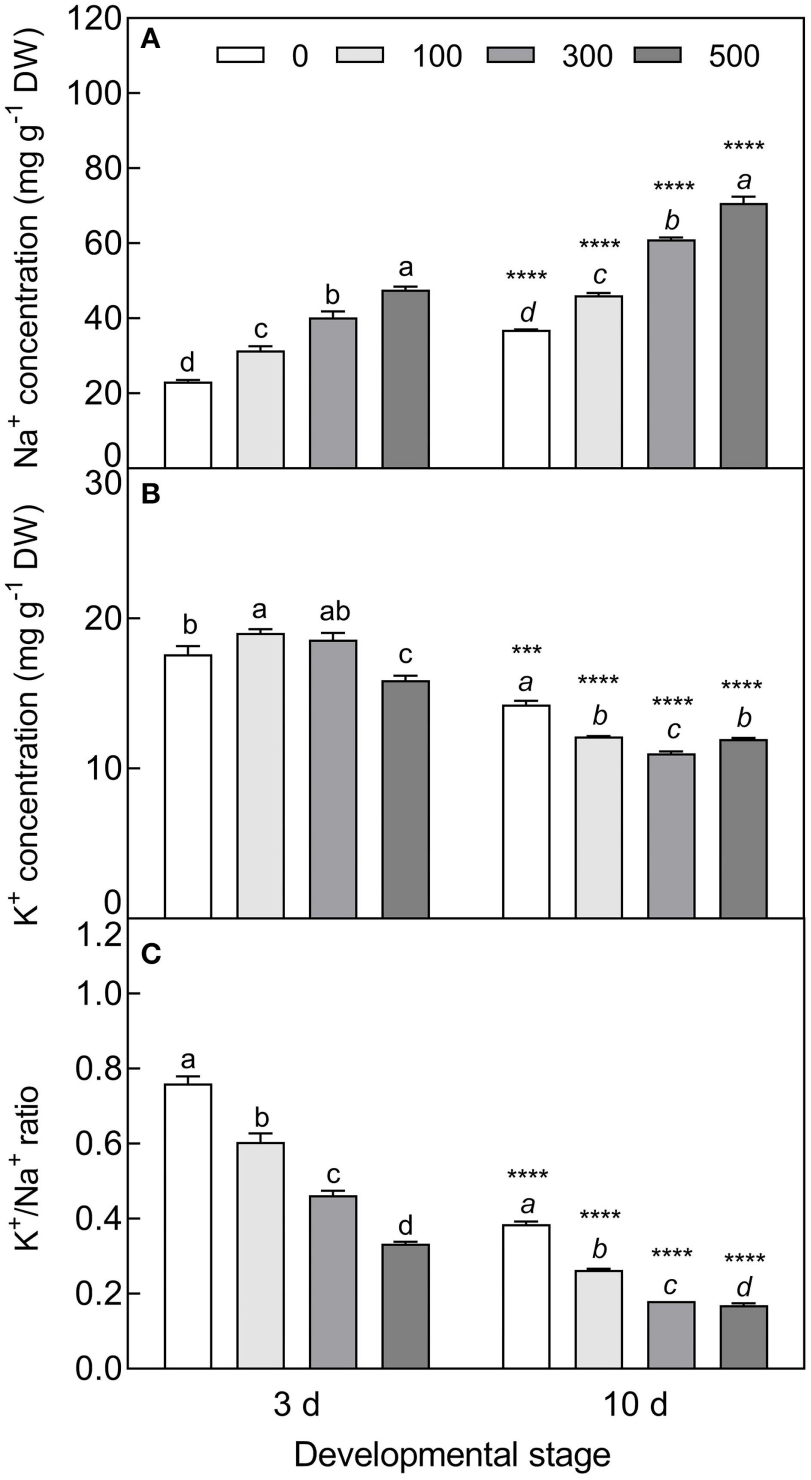
Changes in Na^+^ and K^+^ during seedling development under salt stress. **(A)** Na^+^, **(B)** K^+^, and **(C)** K^+^/Na^+^ ratio. 3 d and 10 d: represent 3 and 10 days after seedling emergence, respectively. 0, 100, 300, and 500: different NaCl concentrations (mM). Bars with different lowercase letters of the same developmental stage indicate significant differences (*P* < 0.05) at different concentrations; *** and **** indicate a significant difference between 3 d and 10 d at the same salt concentration at 0.001 and 0.0001 levels, respectively. Values are means ± SE of three replicates.

**Figure 10:**
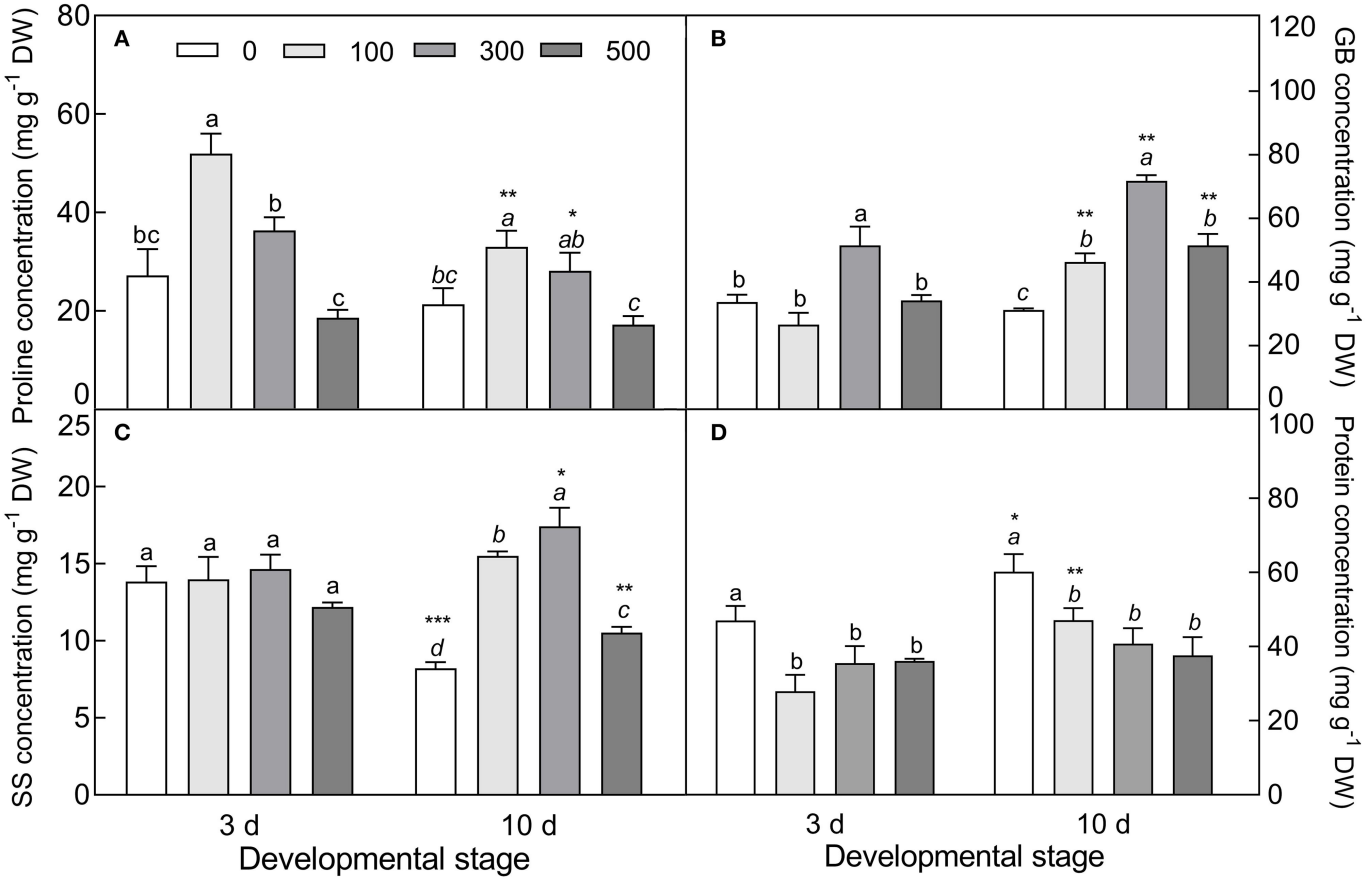
Changes in the concentration of osmotic protectants during seedling development under salt stress. **(A)** Proline, **(B)** glycinbetaine (GB), **(C)** soluble sugar (SS), and **(D)** total soluble protein. 3 d and 10 d represent 3 and 10 days after seedling emergence, respectively. 0, 100, 300, and 500: different NaCl concentrations (mM). Bars with different lowercase letters of the same developmental stage indicate significant differences (*P* < 0.05) at different salt concentrations; *, **, and *** indicate a significant difference between 3 d and 10 d at the same salt concentration at the 0.05, 0.01, and 0.001 levels, respectively. Values are means ± SE of three replicates.

The accumulation of Na^+^ was significantly increased in cotyledons with elevated NaCl concentration (*F*_3, 8_ = 299.1, *P* < 0.0001 on day 3; *F*_3, 8_ = 808.3, *P* < 0.0001 on day 10), and was approximately 1.6 times higher in the 10-day-old seedlings than that of the 3-day-old seedlings (Figure 9A). On the contrary, salt stress induced a significant decrease in K^+^ concentration, especially in the 10-day-old seedlings under higher salinity (*t*_4_ = 27.67, *P* < 0.0001 at 300 mM) (Figure 9B), and K^+^/Na^+^ ratios were significantly reduced with the development progression and NaCl increasing (*F*_3, 8_ = 390.1, *P* < 0.0001 on day 3; *F*_3, 8_ = 1475.7, *P* < 0.0001 on day 10) (Figure 9C). The contents of proline (*F*_3, 8_ = 44.68, *P* < 0.0001 on day 3; *F*_3, 8_ = 15.52, *P* = 0.0011 on day 10), GB (*F*_3, 8_ = 24.08, *P* < 0.0001 on day 3; *F*_3, 8_ = 141.2, *P* < 0.0001 on day 10), and SS (*F*_3, 8_ = 118.39, *P* < 0.0001 on 10 d) were significantly enhanced by salt stress; whereas they were reduced by development extension (for proline, *t*_4_ = 6.239, *P* = 0.0034 at 100 mM; *t*_4_ = 3.143, *P* = 0.0348 at 300 mM; for SS, *t*_4_ = 9.041, *P* = 0.0008 at 0 mM; *t*_4_ = 6.197, *P* = 0.0034 at 500 mM). Total soluble protein was decreased with increasing salt concentration (*F*_3, 8_ = 13.18, *P* = 0.0018 on day 3; *F*_3, 8_ = 16.16, *P* = 0.0009 on day 10), while it was increased with the development progression at lower NaCl concentration (*t*_4_ = 5.998, *P* = 0.0039 at 100 mM) (Figure 10).

#### Expression Patterns of Primary Metabolite-Related Genes

**Figure 11:**
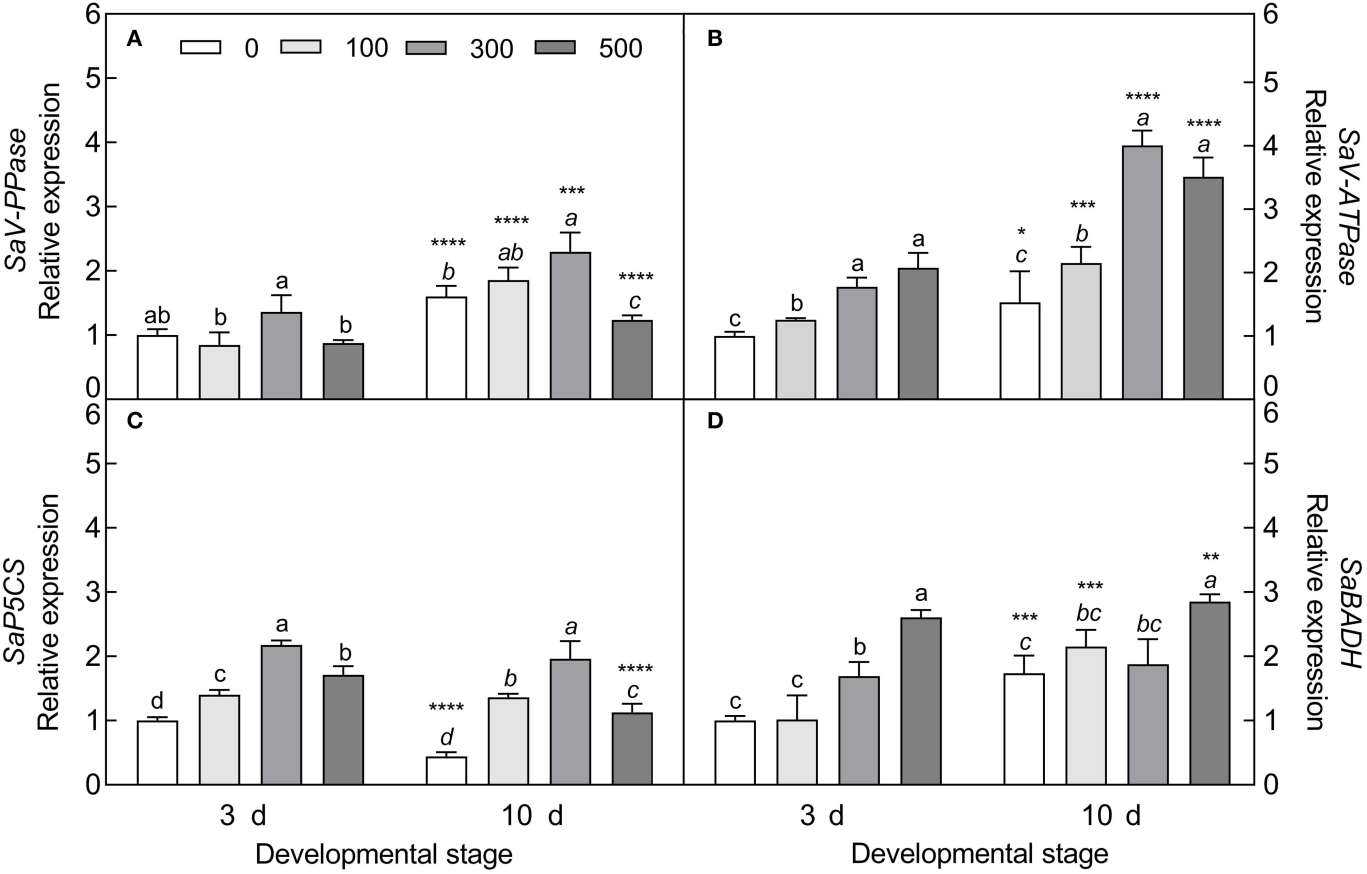
Expression patterns of primary metabolite-related genes during seedling development under salt stress. **(A)** Vacuolar pyrophosphatase gene (*V-PPase*), **(B)** vacuolar ATP synthase gene (*V-ATPase*), **(C)** pyrroline-5-carboxylate synthase gene (*P5CS*), and **(D)** betaine aldehyde dehydrogenase gene (*BADH*). 3 d and 10 d represent 3 and 10 days after seedling emergence, respectively. 0, 100, 300, and 500: different NaCl concentrations (mM). Bars with different lowercase letters of the same developmental stage indicate significant differences (*P* < 0.05) at different salt concentrations; *, **, ***, and **** indicate a significant difference between 3 d and 10 d at the same salt concentration at the 0.05, 0.01, 0.001, and 0.0001 levels, respectively. Values are means ± SE of six replicates.

The expression of four tested genes was all upregulated with increasing NaCl concentration (except for *V-PPase* at 500 mM) (Figure 11), i.e., the vacuolar pyrophosphatase gene (*V-PPase*) (Welch's *F*_3, 9.673_ = 7.911, *P* = 0.0058 on day 3; Welch's *F*_3, 9.62_ = 38.386, *P* < 0.0001 on day 10), vacuolar ATP synthase gene (*V-ATPase*) (Welch's *F*_3, 9.164_ = 66.13, *P* < 0.0001 on day 3; *F*_3, 20_ = 7.98, *P* < 0.0001 on day 10), pyrroline-5-carboxylate synthase gene (*P5CS*) (Welch's *F*_3, 20_ = 191.4, *P* < 0.0001 on day 3; Welch's *F*_3, 10.294_ = 252.3, *P* < 0.0001 on day 10), and betaine aldehyde dehydrogenase gene (*BADH*) (*F*_3, 20_ = 66.08, *P* < 0.0001 on day 3; *F*_3, 20_ = 19.16, *P* < 0.0001 on day 10); especially with 300 mM NaCl (except for *BADH*), the transcript abundance of *V-PPase* (*t*_10_ = 5.771, *P* = 0.0002) and *V-ATPase* (*t*_10_ = 19.49, *P* < 0.0001) was significantly increased in the 10-day-old seedlings, and was 1.69- and 2.26-fold higher than that in the 3-day-old seedlings, respectively. Meanwhile, a significant reduction in *P5CS* expression level in the 10-day-old seedlings was observed under 0 mM (*t*_10_ = 17.15, *P* < 0.0001) and 500 mM NaCl treatments (*t*_10_ = 7.611, *P* < 0.0001) compared with that of the 3-day-old seedlings. The expression pattern of the above genes agreed with the accumulation tendency of primary metabolites involved.

### Identification and Analysis of Differentially Expressed Transcripts During Seedling Development

Differentially expressed TDFs in the two types of seedlings were analyzed with the cDNA-AFLP technique under salt treatment. A representative silver-staining PAGE gel of cDNA expression profiles is shown in Supplementary Figure S1. With 25 pairs of selective primer combinations, a total of 432 TDFs with an average of 18 distinct bands per primer pair were generated, of which more than 90% TDFs were identified between different developmental stages, and only 2% TDFs were differentially expressed between seedlings derived from the two seed morphs.

**Figure 12:**
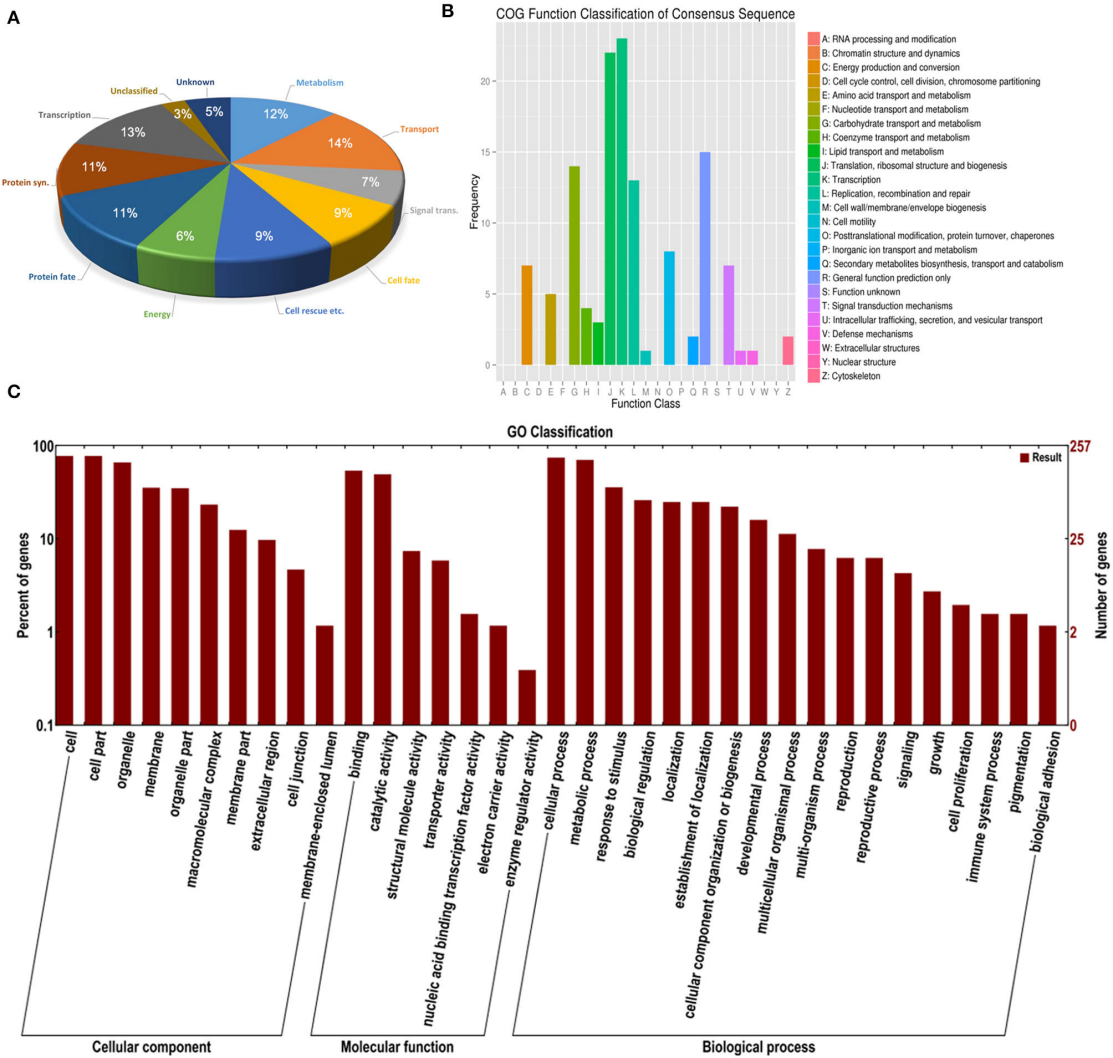
Distribution of differentially expressed TDFs in the cDNA-AFLP analysis during seedling development under salt stress in *S. aralocaspica*. **(A)** BLAST functional classification, **(B)** KEGG functional cluster, and **(C)** GO functional classification. “Signal trans.” represents signal transduction, “Cell rescue etc. “represents cell rescue, defense and virulence; “Protein syn.” represents protein synthesis.

Among the 432 TDFs, 337 produced reliable sequences with a length ranging from 50 to 700 bp. Each sequence was identified by similarity search using the BLAST program against the *S. aralocaspica* genomic database and the GenBank non-redundant public sequence database. Sequence comparison of 328 reliable TDFs (9 had no significant matches) revealed that the majority was unique and shared homology with genes of known function in the database; out of these TDFs, 8% belonged to neither classified nor known function proteins. Based on putative function, the rest of the TDFs could be grouped into nine functional categories, transport (14%), transcription (13%), metabolism (12%), protein fate (11%), protein synthesis (11%), cell rescue, defense and virulence (9%), cell fate (9%), signal transduction (7%), and energy (6%) (Figure 12A; Supplementary Table S4), suggesting that substance transportation, gene transcription, protein synthesis, metabolism, etc. are actively in progress in seedling (cotyledon) development. A functional enrichment analysis based on databases of the GO (Gene Ontology) and KEGG (Kyoto Encyclopedia of Genes and Genomes) indicated that the TDFs are highly associated with activities proceeding during development, e.g., replication, recombination and repair, transcription, translation, carbohydrate transport, etc. (Figures 12B,C).

### Validation of Expression Patterns of TDFs During Seedling Development

Based on putative function, we selected 32 differentially expressed TDFs to validate the expression pattern by qRT-PCR analysis. Among them, 15 TDFs encode genes involved in seedling development, 12 encode genes of osmolytes, transporters, or kinases involved in balancing or regulating stress tolerance, and 5 encode photosynthetic enzyme genes including *Rubisco* (key in C_3_ cycle) and *PEPC* (key in C_4_ cycle).

**Figure 13:**
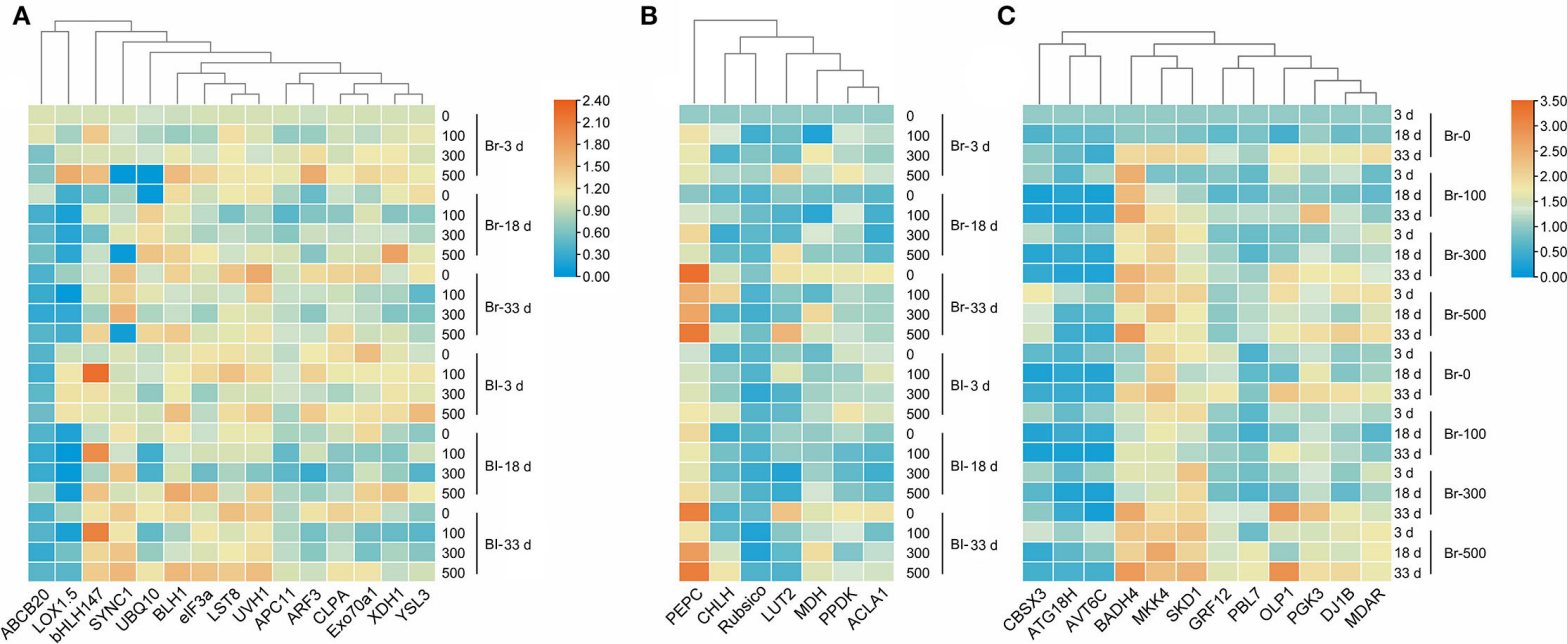
Heatmap of fold expression of 34 TDFs expressed in seedlings of *S. aralocaspica* at different developmental stages under salt stress. **(A)** Development-related genes, **(B)** photosynthesis-related genes, and **(C)** salt stress response genes. 3 d, 18 d, and 33 d represent 3, 18, and 33 days after seedling emergence, respectively. 0, 100, 300, 500: different NaCl concentrations (mM). Br, brown seedling; Bl, black seedling.

**Figure 14:**
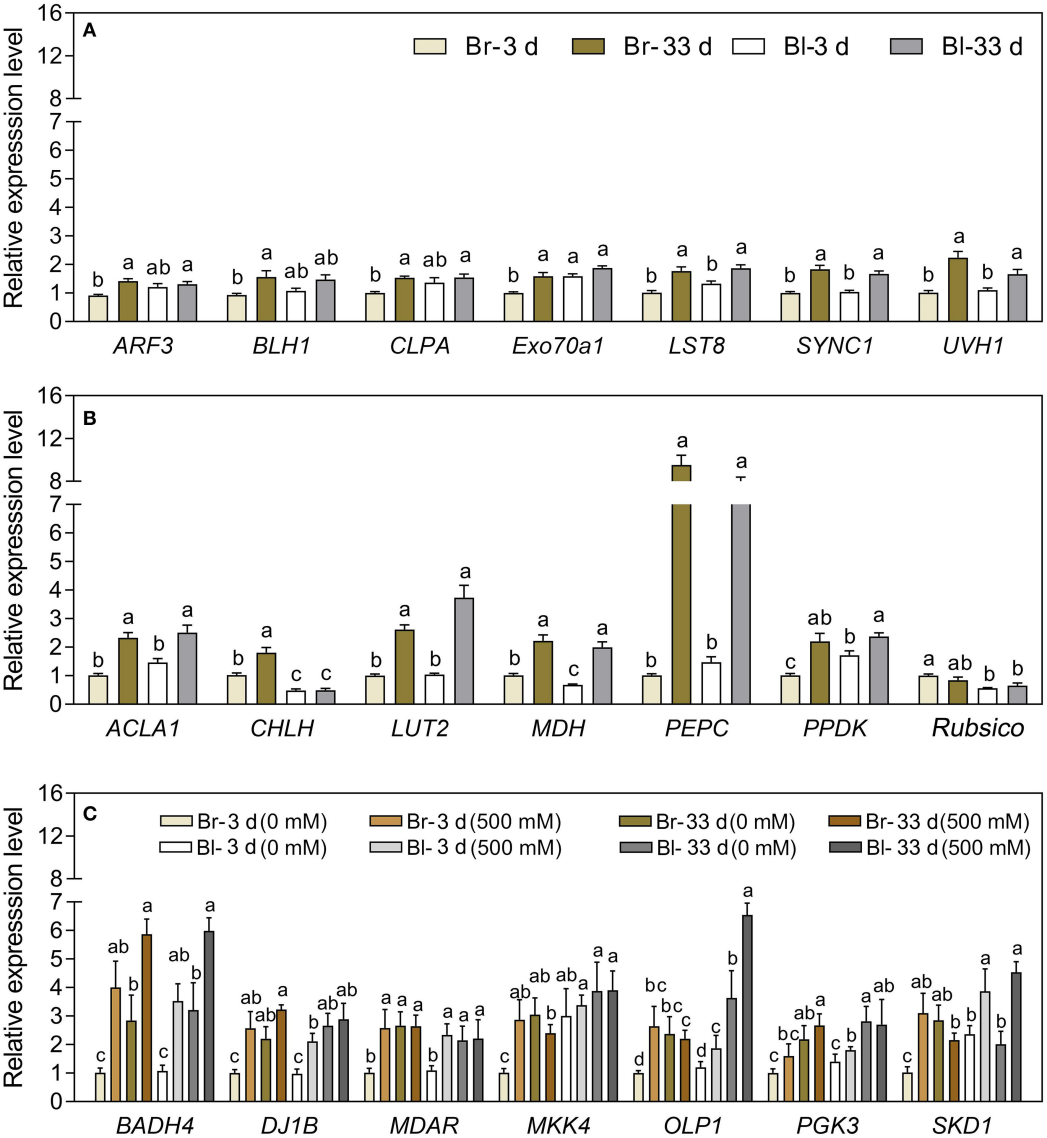
Relative expression of 21 interested TDFs in seedlings of *S. aralocaspica* at different developmental stages or under different salt stress. **(A)** Development-related genes, **(B)** photosynthesis-related genes, and **(C)** salt stress response genes. 3 d and 33 d represent 3 and 33 days after seedling emergence. 0, 500 mM: different NaCl concentrations. Br, brown seedling; Bl, black seedling. Bars with different lowercase letters indicate a significant differences (*P* < 0.05) of the same gene among different treatments according to Tukey's test. Values are means ± SE of eight replicates.

Our results verified that most development-related genes were upregulated with seedling growth (Figure 13A), especially *UVH1* (Welch's *F*_3, 14.62_ = 12.17, *P* = 0.0003), *SYNC1* (Welch's *F*_3, 14.64_ = 22.32, *P* < 0.0001), and *LST8* (Welch's *F*_3, 14.98_ = 15.14, *P* < 0.0001), the transcript abundance of which was significantly increased in cotyledons of the brown seedlings on day 33, and was 1.2-, 0.82- and 0.74-fold higher than that on day 3, respectively (Figure 14A). The photosynthetic genes had similar expression patterns with significantly higher expression levels observed in mature cotyledons (Figure 13B), especially the *PEPC* (Welch's *F*_3, 12.36_ = 98, *P* < 0.0001), *LUT2* (Welch's *F*_3, 14.22_ = 39.27, *P* < 0.0001), and *ACLA1* (Welch's *F*_3, 13.84_ = 21.98, *P* < 0.0001) with increases of 8.42-, 1.59- and 1.29-fold, respectively, on day 33 compared with day 3 in the brown seedlings (Figure 14B). The majority of salt response genes were up-regulated under salt stress in the two types of seedlings with the development progression (Figure 13C). Under 500 mM NaCl treatment, the transcripts of *BADH4* (Welch's *t*_7.448_ = 9.114, *P* < 0.0001), *SKD1* (Welch's *t*_8.177_ = 8.119, *P* = 0.0008), and *MKK4* (Welch's *t*_7.645_ = 7.393, *P* < 0.0001) were increased by ~1.84–2.96-folds compared with the control, especially in cotyledons of the brown seedlings on day 3 (Figure 14C). In general, genes involved in development, photosynthesis, and salt tolerance actively participated in seedling (cotyledon) development from days 3 to 33; among them, photosynthetic or salt-stress response genes presented much greater involvement than development-related genes.

## Discussion

*Suaeda aralocaspica* has developed various strategies to adapt to heterogeneous conditions at different developmental stages, e.g., seed heteromorphism in the germination stage, SC C_4_ assimilation pathway in the adult stage, growth enhancement by salt in the whole life cycle, etc. (Voznesenskaya et al., 2001; Wang et al., 2008; Cao et al., 2015, 2020). However, the specific mechanism for early seedling development in an extreme environment has not been well understood so far. It is known that the seedling stage is usually the most fragile period, and that well-established seedlings should facilitate the survival and successful propagation of the population. Therefore, it is necessary to understand the strategy for early seedling establishment. In the present study, we reported that seedlings of *S. aralocaspica* could tolerate the harsh habitat of early spring through a developmental retardation phenomenon. Its two cotyledons grew slowly but continuously for about 2 months, eventually forming a much bigger size without any visible euphylla. These two large cotyledons could even be existing on mature plants without wilting. To reveal the living situation of such big cotyledons, we investigated the cotyledons in terms of response to different developmental stages and salt stress based on morphological and physiological performances and gene expression. The results indicate that the cotyledons are able to maintain normal growth, physiological and biochemical activities, and active gene expression for a much longer time instead of senescence, which may be a unique strategy for the *S. aralocaspica* seedlings to survive in the harsh habitat during early developmental stages.

According to meteorological data from 2008 to 2012 of the natural habitat of *S. aralocaspica*, the average annual precipitation is about 165 mm but is usually much less during the first 4 months (Cao et al., 2020). The higher moisture of soil in spring along with a much higher evaporation rate promotes the seasonal accumulation of large amounts of salinity on soil surface, which creates a hostile environment for seedling growth (Ma et al., 2018). In the natural habitat, we found that the *S. aralocaspica* seedlings stayed with only two cotyledons without any visible true leaf for about 2 months, and that the cotyledons continuously grew to have a much larger size instead of becoming withered or senescent (Figure 1). Generally, cotyledons of most plants would gradually become exhausted with minerals and storage substances soon after the emergence of true leaves, accompanying with a sharp decrease in the biomass and photosynthetic capacity from the 6th day to the lowest in 20 days, and eventually enter the withered and senescent state (Ampofo et al., 1976a; Keighley, 1984; Jucknischke and Kutschera, 1998; Hanley et al., 2004). In this study, we found that the first leaf bud at the apical growth point (AGP) experienced much slower development within the first 2–3 weeks after seedling emergence, i.e., two cotyledons grew vertically without opening and extending, in which the lower end of the cotyledons (at the junction of two cotyledons and the hypocotyl) was tightly pressed on the top surface of the AGP, which may largely restrain leaf growth from the shoot tip (Figure 2). Moreover, the appearance of the flat AGP within the first 3 weeks also indicated inactive cell division nearby, because the shoot tip usually presents as a spherical structure and the active apical meristem layer leaves a growing shoot behind, pushing the former forward (Lyndon, 1998). On the contrary, in the cotyledons, levels of endogenous hormones GA_3_, IAA, and ZT had no significant reduction and ABA was not consistently increased from the 14th day after emergence in this study, corresponding to the fact that normal physiological activities were still performed in the cotyledons. The existing literature indicates that the energy reserved in the cotyledons may largely act as a potential nutrition pool and play an important role in supporting seedling development (Yi et al., 2015). Taken together, our results suggest that slow but sustainable growth of two cotyledons is in progress, and that an inhibitory effect of the cotyledons on the differentiation of AGP is probably applied; such a unique strategy may keep the seedlings alive in the harsh habitat of early spring while waiting for the favorable coming season (Ma et al., 2018).

The physiological and biochemical activities of plants seem to be associated with environmental factors that vary in different developmental stages (Liu et al., 2010; Kong et al., 2017). Primary metabolites such as organic solutes (e.g., proline, GB, and SS) are not only essential for plant growth and development, but also responsible for stress mitigation during abiotic stress (Chaudhary et al., 2020). To maintain the osmotic potential under saline conditions, halophytes typically accumulate organic solutes and inorganic ions (e.g., Na^+^, K^+^, and Cl^−^) in leaves (Guo et al., 2019). These osmoprotectants may provide protection for cells by raising the osmotic potential, stabilizing proteins and membranes, and maintaining the relatively higher water content under stressful conditions (Sadak and Bakry, 2020). In this study, cotyledons of non-salt-grown *S. aralocaspica* seedlings were able to maintain Na^+^ and K^+^ homeostasis for at least 4 weeks after emergence, whereas the level of organic solutes was significantly reduced in the cotyledons with the development progression, implying that seedlings (cotyledons) grow normally after emergence without requiring excess osmolytes in various biological processes. However, when treated with higher salinity, the seedlings of *S. aralocaspica* significantly accumulated inorganic (Na^+^) and organic (proline, GB, SS, and soluble proteins) osmolytes with the development progression, along with the upregulation of related genes (*V-H*^+^*-ATPase, V-H*^+^*-PPase, P5CS*, and *BADH*), suggesting that cotyledons can positively respond to environmental stimuli by gene activation and primary metabolite production; these results were in agreement with those found in 60-day-old adult plants of *S. aralocaspica* and other halophytes (Cao et al., 2015). Interestingly, the proline content in seedlings developed in spring (Figure 5A) was about 5 times lower than that in the winter seedlings (Figure 10A), which may be the effect of seasonal temperature fluctuation on the seedlings, and we also detected such a change in other experiments (data not shown). In *Brassica oleracea*, low temperature can also cause increase in the proline content of sprout (Šamec et al., 2022). Upon stress, plant cellular homeostasis is disrupted, accompanying with the generation of ROS (e.g., H_2_O_2_ and ${\text{O}}_{2}^{-}$), which may cause peroxidation of membrane lipids and increase MDA concentration (Liu et al., 2021). Excess ROS can be scavenged by plant defense systems involving non-enzymatic antioxidants (e.g., carotenoids, AsA, and GSH) and antioxidant enzymes (e.g., SOD, POD, and CAT) (ElSayed et al., 2020). In this study, the concentrations of ${\text{O}}_{2}^{-}$, H_2_O_2_, and MDA were higher in early stages and decreased gradually with seedling development, which is consistent with the decreasing trend of non-enzymatic antioxidant contents (except for AsA) and antioxidant enzyme activities. These results suggest that it normal for developing cotyledons to carry out various biological activities without needing to cope with the excess ROS produced in the aging process. A similar strategy has been found in *Suaeda physophora*, in which cotyledons play an important role in seedling establishment mainly by generating oxygen and compartmentalizing Na^+^ under salt stress (Zhou et al., 2014). Cotyledon greening is critical for the establishment of photosynthetic activity in the post-germination growth of seedlings (Ojeda et al., 2017). The natural senescent process of leaves has been characterized by destruction of chlorophyll (Lohman et al., 1994) and down-regulation of photosynthetic genes (Brown and Hudson, 2015). In this study, the content of total chlorophyll and activity of PEPC in cotyledons were significantly increased with seedling growth, indicating that the photosynthetic process is active in cotyledons with delayed development. Taken together, our results suggest that developmental retardation may assist seedlings in balancing between internal physiological homeostasis and external stressful pressure.

As a unique SC C_4_ plant species, *S. aralocaspica* carries out C_4_ and C_3_ cycles in an elongated and polarized chlorenchyma cell and has apparently higher photosynthetic efficiency than Kranz C_4_ species in the same family (Edwards et al., 2004; Smith et al., 2009; Liu et al., 2020). Moreover, *S. aralocaspica* operates the same SC C_4_ pathway in either cotyledon or euphylla (Voznesenskaya et al., 2004), which may confer an advantage on early seedlings to tolerate harsh environmental conditions in persistence with two “big cotyledons”. It has been known that C_4_ structure and biochemistry can be enhanced toward a full C_4_ photosynthetic system with the development progression (Voznesenskaya et al., 2003; Koteyeva et al., 2014); *S. aralocaspica* also experiences a gradual developmental transition of the C_4_ structure and photosynthetic enzymes from a young leaf to a mature leaf, or from the bottom to the top in a single leaf (Koteyeva et al., 2016; Liu et al., 2020). Cotyledons are a key photosynthetic organ in early seedlings of *S. aralocaspica*, in which photosynthesis enhancement may play an important role in assisting with development and response to stress. Our data on physiological and biochemical parameters, and gene expression profiling, could support this assumption, e.g., the transcriptional level and enzymatic activity of key photosynthetic genes (e.g., *SaPEPC*) were increased continuously and significantly during cotyledon development from days 3 to 33 (Figures 8, 11). Moreover, suppression of photorespiration is one of the most important events in C_4_ photosynthesis (Uzilday et al., 2012), which can effectively reduce the oxidative load of a cell (Uzilday et al., 2014). In this study, the accumulation of ${\text{O}}_{2}^{-}$ and H_2_O_2_ was reduced with the progression of development (Figures 6, 7); correspondingly, the activity of the antioxidant enzymes (SOD, POD, and CAT) was decreased in the cotyledons during seedling development, which is matched with the process of establishment for a complete SCC_4_ photosynthetic system. This is rational when considering that the photorespiration in C_4_ plants is nearly blocked, thus also preventing the production of photorespiratory H_2_O_2_ (Uzilday et al., 2018).

Plants can regulate signal cascades and molecular networks, and eventually change gene expression profiles to respond to stress (Macovei and Tuteja, 2012). Large numbers of TDFs were differentially expressed in different developmental stages or diverse conditions, which were involved in various biological processes (Gupta et al., 2012). In this study, 328 TDFs were identified by differential expression in the early seedling stage under salt stress, and the GO enrichment and KEGG analysis showed that most of the TDFs were predominantly involved in transport, transcription, translation, carbohydrate transport, metabolism, protein synthesis, and protein fate (Figure 12), which are consistent with those found in *Oncidium milliongolds* and *Lavandula angustifolia* (Qian et al., 2014; Banikamali et al., 2020). It has been reported that genes involved in metabolism and energy are activated early in seedlings and persist in functioning during growth (Guo et al., 2009). Our results suggest that these TDF-related genes should be necessary for the regulation of various biological processes and synthesis of numerous substances in sustaining cotyledon growth and response to stress (Street et al., 2008; Liu et al., 2017; Wang et al., 2019b). Corresponding to the unique SC C_4_ system and high photosynthetic efficiency in *S. aralocaspica*, in this study, ~5% of the 328 TDFs were photosynthesis-related in the cotyledon, and half of which were associated with the C_4_ photosynthetic pathway. Our data indicate that active molecular biological events occur in cotyledons of early seedlings as requirements for development and photosynthesis.

To validate the expression profiles, we selected some TDFs from different functional groups as representatives for qRT-PCR analysis (Yang et al., 2012). Three sets of genes involved in development, photosynthesis, and salt stress response were analyzed, and the results revealed that most of the development-related (DR) genes were upregulated in seedling growth compared with those related to response to stress, especially the *SYNC1* (asparaginyl-tRNA synthetase) and *LST8* (lethal with SEC13 protein 8) genes, which play important roles in regulating amino acid accumulation and nutrition synthesis (Moreau et al., 2012; Arifin et al., 2019), whereas another DR gene, *XDH1* (xanthine dehydrogenase) was downregulated in cotyledon development, which is generally upregulated in leaf senescence (Nakagawa et al., 2007), suggesting that cotyledons of *S. aralocaspica* seedlings are not aging. Genes associated with salt tolerance may limit the uptake and transport of ions and adjust osmotic and ionic balance (Qiu et al., 2017; Riccio-Rengifo et al., 2021). In this study, most of the salt response genes, e.g., *BADH4* (betaine aldehyde dehydrogenase 4) and *SKD1* (suppressor of K^+^ transport growth defect 1, encoding for salt-induced AAA-Type ATPase), were upregulated with increasing NaCl concentration. *BADH* is multifunctional and can increase salt tolerance by protecting the photosynthetic apparatus (Niazian et al., 2021). Salinity stress activates the *SKD1* expression of halophytes in the regulation of ion transporters to avoid ion toxicity; whereas reduced *SKD1* expression renders *Arabidopsis* more salt-sensitive (Jou et al., 2004, 2013). Photosynthesis efficiency is an important index for seedling development and measuring physiological sensitivity to abiotic stress (Wang et al., 2019b). In this study, most of the photosynthetic genes were upregulated in seedling growth, e.g., PEPC (phosphoenolpyruvate carboxylase), PPDK (pyruvate orthophosphate dikinase), and MDH (malate dehydrogenase), which are all essentially rate-limiting enzymes in the C_4_ photosynthetic pathway (Doubnerová et al., 2014; Cheng et al., 2016). Among them, *PEPC* was remarkably active (~9-fold more on day 33 than on day 3) in the cotyledons, suggesting that the C_4_ photosynthetic pathway is working with high efficiency in early seedling development even without true leaf growth. Taken together, our data revealed that the majority of tested genes could positively respond to development or salt stress, and that the seedlings of *S. aralocaspica* could normally grow and develop with only two cotyledons in their natural habitat.

## Conclusions

The seedling stage is the most vulnerable time in the life cycle of a plant, especially under abiotic stress. In this study, we comprehensively investigated the strategy of early seedling (actually with two cotyledons only) survival under harsh conditions in *S. aralocaspica* from the perspective of morphological and physiological performances and gene expression. It was found that the seedlings of *S. aralocaspica* could survive for about 2 months with only two cotyledons, which grew slowly but continuously with a bigger size rather than becoming senesced in the early developmental stage. Meanwhile, the seedlings could positively respond to stress by accumulation of primary metabolites and activation of related gene expression. Such a delayed but persistently developmental morphology was in accordance with the anatomic structure (i.e., flat AGP for a longer time), physiological performance (e.g., homeostasis of phytohormone change, ion absorption and osmolyte accumulation, and active function of PEPC), and the upregulated genes in response to development, photosynthesis, and stress tolerance. This unique developmental pattern may be a smart strategy for *S. aralocaspica* seedlings to maintain the balance between development and energy consumption, allowing the early seedling to survive the harsh environment with higher probability. Our findings should contribute to further understanding of the mechanism for early seedlings to adapt to the heterogeneous habitat in desert plant species.

## Data Availability Statement

All datasets generated for this study are included in the article/Supplementary Material, further inquiries can be directed to the corresponding author.

## Author Contributions

HL, JC, and XL designed the experiments and methodology. JC and HL wrote the manuscript. JC, XL, LC, and MH conducted the experiments and collected the data. JC analyzed the data. All authors contributed critically to the manuscript and gave final approval for publication.

## Funding

This study was supported by National Natural Science Foundation of China (32160057 and 31260037).

## Conflict of Interest

The authors declare that the research was conducted in the absence of any commercial or financial relationships that could be construed as a potential conflict of interest.

## Publisher's Note

All claims expressed in this article are solely those of the authors and do not necessarily represent those of their affiliated organizations, or those of the publisher, the editors and the reviewers. Any product that may be evaluated in this article, or claim that may be made by its manufacturer, is not guaranteed or endorsed by the publisher.

## Supplementary Material

Supplementary Figure S1 A representative result of the silver-stained cDNA-AFLP gel showing differentially expressed genes at different developmental stages in seedlings of *S. aralocaspica* under salt stress. **(A)** Seedlings from brown seeds, **(B)** seedlings from black seeds. 3 d, 18 d, and 33 d represent 3, 18, and 33 days after seedling emergence, respectively. 0, 1, 3, 5 represent 0, 100, 300, 500 mM NaCl treatments. The red arrowheads indicate TDFs with a differentially expressed pattern. Each group was amplified with a different pair of selective AFLP primers.

Supplementary Table S1 Relevant parameters of four phytohormones.

Supplementary Table S2 Sequences of the adaptors and primers used in cDNA-AFLP analysis.

Supplementary Table S3 qRT-PCR primer sequences used in the present study.

Supplementary Table S4 The 328 differential expression TDFs identified by cDNA-AFLP against known gene sequences using BLAST.
