# Supplementary material for: The Developmental Delay of Seedlings With Cotyledons Only Confers Stress Tolerance to Suaeda aralocaspica (Chenopodiaceae) by Unique Performance on Morphology, Physiology, and Gene Expression
Source: Front Plant Sci. 2022 Jun 6;13:844430. doi: 10.3389/fpls.2022.844430 (PMC9208309; doi:10.3389/fpls.2022.844430)
Supplement: Supplementary file 1 [file Data_Sheet_1.docx]

Note：“Metabolism” is highlighted in yellow；“Transport” is highlighted in green；“Signal transduction” is highlighted in [dark](javascript:;) [green](javascript:;)；“Cell fate”is highlighted in pink；“Cell rescue, defense and virulence” is highlighted in gray；“Energy” is highlighted in red；“Protein fate” is highlighted in blue；“Protein synthesis” is highlighted in purple；“Transcription”is highlighted in cyan；“Unclassified” is highlighted in dark red；“Other unknown” is without highlighted.

>TDF1-2

GAGCAAAAATGCACCTGTTGTTGGGTTAGTACTTCAAAGGAGTCATATTGTAACTGGTGATGAGTCTCATTATGTTGCTGTGATTATGGAGTTGGAAGCTAAGGGGGCTAAGGTGATCCCTATTTTCGCCGGAGGGCTCGATTTTTCGGGGCCGGTTGAGAAGTTCTTTATTGATCCTGTGACTAAGCAGCCTATGGT

>TDF1-4

ACTGGGTTTGAAGTCCTCACGAGCAGACCGTTGAAGACGGAGCATAAAGAGTTCACCT

>TDF2-1

CGTAAGTTAGAGGGTGAGATAGAAATAAGAGTAGGTGGAGGTGATGCGTTTCATGAACTCTTTCAAAACAAACCTCATCATATAACCAACGTTCACCCTAATTTTGTTCAAGATTGTGAATTGCATGACG

>TDF3-1

GGCAGACTGTTGGTGTGATTGGAGCTGGTCGTATTGGGTCTGCCTATGCAAGAATGATGGTTGAGGGATTCAAAATGAACCTGATATACTTTGATCTGTACCAATCAACCCGCCTGGAGAAGTTCGTTACAGCTTATG

>TDF3-2

CATAGGGGCTGCTCCAGCCTCCACCTTCTTTACGGAAGAACTGAGTGTCTTCAGGCCAGTTGTTGTAGTGGCCAGCATCAGTTGGGCCTGAGTGACCCCATTCAGGTGTCCCATTAGCTTCTGCAGCTGT

>TDF3-3

GATCAAGCTCTCCCTAAATGTTGGCTAGACTGATTACACATCAAGTGCCACGGTGTCTACTTCCTATCAACCAAATGGTTCAATTATTGTAAACCCACATCCTGAAGGCTTCCACATGCTCGAGCCTCAATCTTTGTGCTTCTGTTCTTGCATCTGTGGC

>TDF4-2

AAACGTCTCAAGCTCACTGCCCTATGTGCCGTCGAGAATGGCACTTCAAGGATTGAAAGGCAAGCTATTTTGATCATCTCATCCACCATCTTTCAGAGAATGATTATTAGTGCCTAACCGAACTTTGGTATAGTTCTCAACCTTCCTCCATGGTGATGCTAGTTTCTGCTTCAATGTATACCATCTTTATCTTTCAATTTTGTGGAGCAACTCGCTGG

>TDF5-2

TTGAAGGCCGAATAGTAGCCTGCGAAGGATCTAAGAATCCTTTGGGACACCCAATTGAGTTCATATGTCTTGACCTGGACGAACCAGCAATCTGCAAATATTGTGGTCTACGTTACGTTCAAGATCATGGTCATCACCATTAGGAGCTGATTTTTGGTTTTGTCAATGTAATTCCCCATTTGATGGAATTTGAAAACTAAGAGGATAGTTGCAATGTAATTTGCATTTATGTGGTTATATCCTTCTTTGCTTGTAAGCTTTGTCTGCTTGTTATTTTGCTTCCATAACAACAGTG

>TDF7-5

AGCCATTTCAGTTTCAAAGACCGAAGGAAGGGTTGGACAAACTACGAATGTTGTCATTGGTGGAACGGTAACAGATGATTCAACCAATGAATGGTTGGCTTTAGATCAAAGGGT

>TDF12-1-1

CTTCAGCCGTGGAAAATCCCTGCTCCTCCATCCCAGAAAGGATCATTGTAGCAGCGACAGCATAAGTAACTCCAGTCCAAATTTCACGTGATTGCATGCAGGTCTCATCCACCTTCCCCTTCGGATGCATCCCATTTACAGCACCCATGCGACCTCCT

>TDF12-1-4

GATAGATCATTGCAATTGTTGGTCTTAAACGAGGAATTCCTAGTAAGCGCGAGTCATCAGCTCGCGTTGACTACGTCCCTGCCCTTTGTACACACCGCCCGTCGCTCCTACCG

>TDF12-1-5

CTGCTGGAAGTGTTCTGCGATCTGCTTTGGCGGGTGGCCTTGCATCTGGGCTCTCGACATCTTTATTACACCCAGTTGACACTATCAAGGTAATTGTTGATGCTTCAACT

>TDF12-2

GCATAATGATGTCTCTAGTGTTCTGATCCATGCCTCCGTGGGTCGCAGAGACGGTGTGATCACGAGCTCTCATTTGGTCGGTCAACCAGTCGACCTTCCTCCTGG

>TDF12-2-1

CTAAAGGGCCGGCATATTCAGGTCCAATACGCTGTTGTATACCCGCGGATATTTCTCTTTCTAACCATACAATTACTAGTACACCTATTGTGATTCCCAATATGAGAATCAAAATGGGGAAAAGCATCCATATAGTCTCAAAAACCTCCT

>TDF12-2-5

AGCGGATTGTAGAAACAAGGAAAGTAATGGCATATCTCATGATCACCGCCTTGGCCACCTGTAGGAG

>TDF12-3-2

AGGCACAGATGCTTTGTTGCGTCTATACGCATATTGGTCTCATTTAGAGCTCACTTTGGCAAATGATATAGTTGCTGCCCGTGGAGTTTGGGAGAGCTTACCGACGATTAGTGGCGCCATGCTTGAGGCTTGGGAAGGATATGTAAAGATGGAGATCAACAAGGGGAACCTGAATGAAGCAAGATCGATCTATCGGAGGTGCTACACAAAGAGATTTCCGGGGTCAGGTTCACAGGACATGTGCCAATCCTGGTTGAGGTTTGAGGAG

>TDF12-3-4

CTGACTAACATAAACAAGGCTGTCCAAAGAAAATGAACATCTATCCAAAAGTAAAGCAGATCCCCCGAGAGTTTATTTGCGCACCAGCAATGAAAGACCAAAAGGGAACACTCCTGACCTGTGTACAAAAATAACACATTCAGGGTTTACCGCACAGGTTGTTGGTTGTAATGGTTCTTCTCTTTCTTCTTCGCAGCAGCAAGCTTCGCACTCCTTGCAGCAGCCTCTGATGCTGCTGCTTTGACAGGAGTCATCTCTTTAGTTGATTTCTTCTTCT

>TDF12-4

AACCCAAATCAGTGGAACCTATGGAAACTACAAATACCAGGGCAACCAATGCGTTATTGCTACAATAAAGATCCACACTAATTTATGTCCATCTGGGTATGGACCATATGGTCAAGGATGCGGTGTGGAAAATGTGCACTGTTTCTCATCTCCACTCCCAACCGAAGGCCCTATCGTCGGATTCTTCGGAAGGCACAACAGCTACCTCGAGTCTGTTGGAATCTCCGTCAAGAAGGAGTGTGGATGC

>TDF12-4-1

AGACATTGAAAGCTGGGACGGGCAAGACCATGTTCTTGTTTCCAGCAATCTCTGCAATGTGCTGGTACAGAG

>TDF12-4-2

CTCGGCGAGTTTGTCTTGGAATATGGGCGAGTTTTCCATGGTAAACTATGATGTCTCCTATGATAACTTTATGCT

>TDF12-5

CCCTCGCGGAAGACGGAAAGAAGTTTCTCATCCGAACGAGAGTTCTCAATGGTGATGACATCAGCATCCATGTCGATGATGGAGTGAATGATGTCATCAGTAGGGTAAAACTAACCTGTCTCACGACGGTCTAACCCCAGCTCACGTTCCCTATTGGTGGGTGAACAATCCAACACTTG

>TDF12-5-2

CTCCAACAGCTGAAATTGCCGAGCCAGTCCCGCAATTTCCTGCAAATCAGGCAAACACTGTAACAGAAAGCGGGACAAACCCTCCCACAGTTTCTGGTGCACCAAACTCTTCTCCT

>TDF12-6

AGGAGGACACTGAACTTACTGTTCTGTTCTTAGGCGAAACCTCCAAGGCCCACAAGCGCG

>TDF12-6-2

CTGCGAGAGTGGTGCAGATTTGACTGCCCTCTACCTCCAAAAGGAGAAATACTTCTCTCTCTACGAGACAAAGGACGCCGGTACT

>TDF12-6-4

CTGGATCACAAGTTCAGCAACCTAATCAAGAATTGTCTGAAGCTTTACAGCCTCAGCATTCAGCATCCAAATCTCCAGCAGCTTCATCATCTGATTCTGGTAACACTTCTAGTGCAAAAATGGATATTGAAGTGGAAGCCACCAATGACGAGAAGGTCGCTGCT

>TDF12-7

GATGTGCCGCCCCAGCCAAACTCCCCACCTGACAATGTCCTCAGTAGGGTAAAACTAACCTGTCTCACGACGGTCTAAACCCAGCTCACGTTCCCTATTGGTGGATGAACAATCCAACACTTG

>TDF12-10

CTGGAGATCCTCCCAAAATTTCTCAGAGAGCCCACCACATTCTATTCACCAAAGAAACTTG

>TDF12-12

GATGCTTAAACACGGGAGAGCTGGTGTTCCTATGGAAGTTATGGGATTGATGTTGGGTGAATTTGTTGATGAGTATACTG

>TDF12-17

CCTTCCAAAAGGAGAGTTCATGCATAATATGCTGTCTCAGCAAAAAACAATTATCAACGCAATGGC

>TDF12-20

ACACATGCTCCACGCGCGCTACCTCTCACGCGCCCACCTCTCTCACTACTCCCTCAGTGACGCCCACGTTTCCGT

>TDF12-19

CGTCGCGAGTTTTTTGAGAAGAGATTGGAGAAGGAAGCAGAAAAATTGCAGCTGCTTGAAAAGGCCAAGGTGAAATCAAATGCTGACATGAGGAAATTATCACAAAGT

>TDF12-24

CACATTGGAGCTTGATCCTGAATTCATTTCCGCAAGAACCACCAAACATCTTAGTGACGTTGTTCCCGCATGGCTTAGCGACGACGGAGCCGATGGCATCCACGATGAAACCATGCCTGATGATAACCTCTTTGATGCTTTCATTTTCTTGAAGTGTTATGGAATAGTTTTGAGGACATTGTGAGCCATAAGGTCCATGTTGGATAATCACTTGTCTCTTGGCGTTTGCTTGAGCCATTTCCTTAGCTGAGAAAGGTAACAAAAGACTTGAG

>TDF12-26

TTAGCTCCAGAAGCTGCCTCAGATAACGCTTGCAGAAAGCTCGACCTCTCAACTTCTGTCGCTGCTGTTCTTGGAATCAAAGAAACACAATCCTTGGCCCATTCCAGAGCTTTCCCTTGATAAGCTCGAGCTAGTGCCATAAGTGCATACGTTACTGTTTCAAATCTGTTATTTGGGAGAGCGCCTGTGAGGGATGCTATCAAAATTCTGGTGATAGCAGCCCCTCTTGGAATGATCACACTGTCCCTTATGGACTGATACTGTGCACTTTCAC

>TDF13-1

GCAAGCCCCAGCCTGATTGGGTGGGTAGGAGGAAGTTCAGCCAAAGCGATGTCCTGAGCAGACTTGTAAGCCAACAAGGTGTGTTCCGCAGCTTCCTTTCTCTCAGCTGCAGTC

>TDF13-3-4

AGGGCCAGGGGCAGAGTCCAGTAGCCAATGGCTCAGTAGATGAAGCTCATGACATAACAAGTACTGTTGTTCCAGAAGAACCAAGAAGCATAGAAAAGGAAGAAGCAGAAGAATCAGTCCCTGAAGAATCAAATGACAGTGATGTTACTCTCGGTGAGCAGGTTGTCACTGGAGAGGCCGTTGATACAAGTTCGGATGAATGGTGGCAAGAAGCCAACCCAAAATTGCGTACTGCTAATGCTAATGAAAATAACTTCCGCAGAAGACGGCCTAATCA

>TDF13-4-2

CCTCAGAGGATAAGGATAAACAAGGCGGAAGCGGCGGCAGTGGTAGTGTGCAGCTGAGCACTGCTGAAAGACAAGAGATTCAAATGAAGAAAGCAAAACTTGTGACCATGCTTGATGAGGCTGAGCAAAGGTACAGGCACTACAATCACCAAATGCAAATTGTGATCGAATCGTTTGAGCAAGCAGCGGGAATGGGATCAGCAAAGACATACACGGCGTTGGC

>TDF13-4-4

AAGGGTCATCTACTCTCACTAATCGGCGGCTGCTATCAACCCACTTGCACTTGATCAAACAAACTTTGAAAGAAGAGTAGTCCAACTCCCATATTTCTTGTATTTTACCATAGTGGAACTTCTTAGCATCGATTGGAGATTGGTCTTTAGCATTAGCATAATATGTGGACGAGGGCATAACAGAAACTCCACTATTCTATAAACATCGGGACTTTTCA

>TDF13-6-1

CAAAAAGATACTTGGCAACTGCATCATCTGATCGTACAGTCAAGATTTGGAGTGTAGACAATCGTTTCACATTGGAGAAGACACTTTCTGGGCATGAACGATGGGTGTGGGACTGTATATTTTCTGTAGATGGCGCCTACCTCATTACAGCTTCCTCGGATGGAACAGCAAAACTATGGTCCATGATAAGTAATGAAGTTATTCAAACGTATCAAAGTCATCACAAAGCTGTTGTTTGCTGTGC

>TDF13-9-3

AAATCAAACAAAGTATGTAGGCCTTATTAGAACACACAAAACTAACTAAATCAAGCCTTCTGAGTTCCTGGCTCTTTGACAATGGTCACTGGTACATGAGCATTATTCATCACATAGTTCGCCACACTCCCCATGATTATCC

>TDF13-10-1

CGAGCAGAGAAAGTACCAGCAGGGGCAAAATCGGAATCACCAAAAGCATGTTCAAGAGTGAATTGAAGCTTAGAATCAACATCTGAATCTGAAGATCTTCTTCTTATTGTAGGAGAAGATGAATGAATTGGTG

>TDF14-1

AGTGTTGAAGATAGGGATAGTATACTCCGAGGGCTTGACGAGGTGGAGAAGCGTATAGAGGCCG

>TDF14-3

CGAGCTGATAGATTGGTCGGCCAGGTACTAGGGGAGGTTGGATCTCTTCCAGATGTTTTCGTTGAGCTAGAG

>TDF14-4

CAATGCTGTGCACTGCTGAGCCAAACCATGAAGCTGGTGACAGGCCCTGTGAAGGGCCTCCATAACCTCATCCTTGGTCTTATTTTG

>TDF14-5

CAGAGCTTTTAGGTGAAGGTTCTCAACAGCCACCTGTAAACGATTCTGCTAGTGCTACAATAGAGG

>TDF14-6

CAAGTGTTGGATTGTTCACCCACCAATAGGGAACGTGAGCTGGGGTTAGACCGTCGTGAGACAGGTTAGTTTTACCCTACTGATGACAGTGTCGCAATGGCA

>TDF14-7

CAAGTGTTGGATTGTTCACCCACCAATAGGGAACGTGAGCCGGGTTACGGTGCCCAACTGCGCGCTAACCTAGATCCCACAAAGGGTGTTGGTC

>TDF14-9

CAAGTGTTGGATTGTTCACCCACCAATAGGGAACGTGAGCTGGGGTTAGACCGTCGTGAGACAGGTTAGTTTTACCCTACTGATGACTGCAGCTGCGACCCATGCACCTGC

>TDF14-12

TCAAGCGGGATGAAAATGGGTGGATACGGTGATGAGCCATACTATCTCCATGCAAAGCACATGTACAACTTGGACAGGATGAAGCACCAGAAGTTGAAGATGTCCCTTGGTGTATTGGCTGCCTTTAGCATCGGAGTGTTTGTGCCTGTTTATGCAGTCATTTTCCAGCAAAAGAAGACTGCATCT

>TDF14-14

CGAGTATCTCCGAAGAGCCGAGGAGATTCGGACTGTTCTAGATGAAGGTGGCGGGTCGGGCCCCACTTCGAATGGTGGCGATGGGGCTGTGGCGGCCCGGCCCAAGCCCAAATCGAAGGATGGAGAGAAGGATGGTGAGGATGCGGAGCAGACGAAGCTGAGGTCGGG

>TDF14-18

CCATTTTGTGGAACAGGTGGTCTAGGCTGTGCAACAAAGGGTCCTGTTGTGGCAG

>TDF14-22

GAGCGAACATGGTCTCACCATATCTCCTTTGAGAGGAATCACCGGGAGGTTATCGCAATTGCATATTTCGATCATGAAACCGTCTGGGTCATGGAAGAAGAGCTGATCCACGTAAATACCTCCTTCTTCAACTGTTGCTCTTACATGTTTTATATCCATCTCCTTCAACTTCTTTTCCACTGCTCCCATGCTTTCACACTGAAAGGAAATGTGATTATCTTTGG

>TDF14-23

CATGATATATTCTCAAACAAGCTCAATTGAAATGAGTCAATTAGTTATCAGTGACACGAAATCAAGGGCTCATCCTCCCCAAATACATCAATCACAAATCATCAGTATTTGAGTTCAATTACAAATATAAAACCATAACCATAAACATAGTATGACAACTCTCATTTCTTGTTGGTTCTACCTCCGGAGAAACCAACACCTGCAGCCTGTGCTGCCTTTTTAGCAGCCTTTTCTTGAAGCGCTTTCGCATCCCTCTCACGACGTTGTTCAGG

>TDF14-25

AAGCGCAAGGCCTGTTGCAAAGACATCTTCGGCGTGAGCCCTCACATTTGCCATCAGGAAATCCTTCGGTTTGGCAAACACTGGCACAGTTCCTGTTCCTAGTGCAGATTCCC

>TDF14-31

CCTGAAGCCACATTCTCACCCAAAAATGTAACACTGACTCCTGCAGTTGTCTTGTTTTTCTTCAAGTTC

>TDF14-32

CATCGATTCTTGAGCCCCATTTGAGGAACCTAATAGAATACATGCTGCAAGTGAACAAGGATGGTGATGAAGAAGTGGCACTTGAAGCTT

>TDF14-33

CGGGTAAATCCACGTCATTCCACTCTATTCCTTGTAAGTCAAAGATTTGTCAACAGTTGGACCAACCTGGGTGTGCTATACGTGCCAATAAATGCGCGTACCAGGTGGCGTACGGTGATGGGTCGTTTACTGTTG

>TDF21-1

CATGACAAAAGGGGCTGATGAATTCACCAAAGAGGCGCTAACTCAAGCTATTGTCTCTCGAGCTGATGTTAATATGAAGG

>TDF22-3

CAATTCCTTCATTCACCATATCTACAGCAATCTTCACTGCACCTTTCCCAGTACGCTTACCAGAACGGCATTGCAACATCCATAGCCTATTTTCTTGTACAGT

>TDF23-1-3

CAACCAAAGGAGCATATTCACGAGTTGCAGCAGGAAATATTGCTTCCAAGTTCCCACATGCCATGG

>TDF23-1-4

GACAATAGTGGCAGTAGTAGATGCTAGTAATTTAGCTGGTCTTCGTATGTTTTGGGATACTCCTATTCCTGAGGAAGTGAAGGATGTGGTTGAAGAACTTATTGTATGCCAG

>TDF23-5-2

GGTGGATACGGTGGTGGAGGCGGTGGAGGTTACGGTGGATCACGTGGTTCTGACGGTGGTAGCTGGAGGAATTAGTTTTTTTAGGAGTGG

>TDF23-5-3

GTACCTCTTTTCCAGAAAATTCCTGGGGGTAGTCCTGTCCTTGGTTATTTAGCTGCTGGCATCTTA

>TDF24-1

ACCAGGGTTGCCAATGTTGATTGGCCCAGTGTTCTCTCCTTCCATGAGCCTGATGAGACCATCAACCATGTCAGACACATAGCAAAAACTCCGAGTCTGAGTGCCAGGAGCTTGAACTGTCAATGGTTCATTCCTAAGTGCTTGAGCAATGAAGTTGCTAACAACACGACCATCATCGATATTCATACGAGGACCATAGGTG

>TDF24-2

ACCAGCTCCCTTGTAAATGGAGGCAACAATCCTGCGATTTGCTCATACCAGAGCATTGGCCTGACAAATGCACTGCCAGCTTCTTCTCCAATCTTCGTGCCTGCAGTGGCTGAGAATGCGCAGAAAAACTTCTGGGTTGATTTCCTCATGGGAGGAGTTTCTGCTGCTGTTTCAAAGACTGCAGCAGCACCCATTGAACGTG

>TDF31-4

CTTCCTCTGAACGCTGGAATGATGCATCAAATCGCAATGCTGCCCATGAAACAAGGCGTGATAGCAAATGGACTTCCAGGTGGGGTCCTGAAGAGAAGGAGAAGGAATCACGGCCAGAGAAGAAAGCAGACCTCGACAAAGAAGATGCTCATATTGACAGTCATTCTGTTGGAACTGGCAATCGTGATGCTGAATCACGTGATAAATGGAGACCTCGCCATAGGATGGAAGCTGCTTCTAATGCACCTACTCCACACCGTGTTGCTCCTGGCTTTGGCCCTGATAAGGGAAAGGTGGATGGTGTCAACATGGGGTTCACTGTTGGAAGAGGAAGGTCAAGTGGTGCACCAGGTATGAAGGCTCCTCTGGGGAGTACAAGTGGAACTG

>TDF31-1

GGGTTTTTCCGCTGCAGGCAGTGGTTAAAGGGCAGGGTGGGAGTAAGAGGAAGGTTCGAGTGGCGCGAGGGAAGGGTGGGAATTCGGATGCTGTGGAAGAGG

>TDF31-5

TTTGGGATGATACAGTGAAACTATTTCATATTGAACCTGATCAAGCCAAGAAGAACGTATTTCTTTCTGCCCTTGCAGAGAGATTTAGGTGTTTCAAGTCCAAGTTAGTGAGTGGTTGGATCA

>TDF31-6

CATTTCTGACACTTGTCACAGCCAACCCAATTGATTGGAGGTGGTTCAGATATGGGCTGCACATTCTCTGGCACAGAAGCATCATTTGAATACCCTCTGTGCTTCGTCCATGAAGTTGCTC

>TDF31-7

CTCCAGCAATCATTCGTCACGGTTCTCAAGACAGAACTTTGCCGACTTAGTGGTGCGAAGATATATCCTGGAAGGGGTATCAGATTTATTCGTTCAGATTCTCAGGTGTTCCTTTTT

>TDF31-8

CTGCTAAAGTTTGTATTAGGACGCTTTTCGCTTCGGAAAAACGTCTTAGTGAACAGATCTTTGATGATTTGAGCTCACCTTCTTCATCATCATCATCAACTTCTTCCACGAGGAATGTT

>TDF31-10

AACAACTGATACAGCGATGACATTTTCTGAGAATATCGCATAATGATAAAGGGAAGGATCCTCAAACTCATCTTTTGGCTCTTCGTCCTTGTACTTCTCTGGATGAGCAACTCGTTCTCCAACAAGTCTCATAGCTAGACAATGCAGACTTTTAGGCACAGATTTTGCAGCAACAAAGCTAGCAACTGCACCATTCTTCTTTG

>TDF31-11

TGACAGAGACTCAACTAGCTATGCTGCTTTGGGAGCCAATTATTGAGTCTTCTGATACCA

>TDF31-12

CCATGTCTGAAGACATTCAACCTTTGTTATCGGAGATTCGTGACACTGGCTTGTTGAAGGAAGTTGAAAGTTTGACTAAAAGCCTTACACAGGCAAGTGAAGATTTGAGAAGG

>TDF31-29

GGAGGTCGCCGTGAAGCAGCTCAAGTCGGGGAGTGGACAGGGAGAGAGAGAGTTCCAAGC

>TDF31-30

ACAATGTCACCTAGGCAATTGCAGCAGCAAGAGCAATGTGGGTTAGCTTGTGGTGTTGATTCGG

>TDF31-32

CCCGCGGATCATTGCCACAAAGACAATGAGGTTTGTGACATAAGGTATGGT

>TDF31-35

GCCATAAGAGCTTCATCTTCTGCAGCAATATATAGAAGAGCCTTTGCAAGGGAGATATGTTTGTCCTTTGATTGGAAAGAGATCTCCGAAGTAAACTTCTCCCTCGCATCCCTGAGGACATCCTTATAAAAATTAGGGCCACAAGAATCCTTCTTCACTTGTCCACTGAAAAGTGGAGAAGCCGAAGCAGCTGCTATGGAAGTAGGATAATTGGTGCTAATCCTCTTCTTCCTTTGGTGCTGAGTTGTTGCCACCATCA

>TDF32-1-1

GACTAAGGATAAAGGTTGGGGCCTGAGGTCATGGTATGCTATTCGGTCTGGAACTTTTGTTTGT

>TDF32-1-4

GAGACATTGTGCCAATGGAGAACGCACAGCTCCTCTAGTCAAGTTTGAAACAAGCTTGCTCACAGCTAAACGGTCATTTTGTCTAATCTGGTGCAGCAGAGCTAGAGCATGAAATTGAACCAAAGCAGCACGTGATTGAACAGCCTCTTGCACTTCATTACTCCATCGTTTCACGATCTCGGGATTGCTCTGGAGCAAGT

>TDF34-1-1

TCACGAAGTACCTACTAGAATCAAGCGCAGGTTCAACCGACTGATCGCGGAGTCCAGGGTAGACGAAACAAGCGGCGAAGAGGTCGCCGGAAAGAGGATCTTCGAGGC

>TDF34-1-4

TCCAACTCCTTCTTACTCTGCTTCTGCTCCTCCCCTTCCTTCTTCCTTCTCCCCACCTCCTCCTCCTCCTTCTGATTTCTCTAATTATTACGTTTCCCCTGATTACCCTCCTCCATACCCTCATAATTCCGATTTTTCCATCAATTCTACCCCTACTGCTCCTTCCTTCCCTCCTAACCCTAACCCTAATCCCCAATTCCCAAATTACCCCTACGATCTCCCTCAAAATCAGACTTCCTACTATGGTTATGATCATAATCATACCAATCAAGCTGCCCCGAACCCGAATTTGAGTTTGAACAACACTTTTTCGGGTTCGCAGTACTCGGCTTACGAAGACGGTACTCGTTGGGGTGATTACGGGGAACAGGACAATAGTGTGTATGAAGGTGCGTATAGGTATGAGGGAGGGAAGGTGAAGCCGTATGGGGCGAGAGGGACGGCGGGGTTTTCTGGCGC

>TDF34-2-3

GCCACTGCAACTGACACTCCTCCGTTGTCAAGTAGCGGCCCCATAGTTGCAGAACCTGACCGCAAAGTTCCTCCGA

>TDF34-4-1

AGGAAAATAGATTACTCCATGCAACTCAATGCATCTCGGATCAAAGTCCTTCAAGCTCAGGATGACTT

>TDF34-4-5

TACAGCACACACTTTATGATGCCTTCTTATCTGTTCTCATTTGGAAAGCTGAGGTCTTTGTGCTTGACAAATTGTGCTTT

>TDF34-5-1

ATCATCAAGGGCAAGAACTCCGGGCAGGGGTTTGCCTTCCAAAAGCTCCAAGCTGGCACCACCTCCAGTAGAGATATGGCTCATCTTGTCAGCAAGACCAACCTTCTCAACAGCAGCAACTGAGTCACCTCCACCAATGATTGTGGTCACACCCTTACCACTAAGCTCGGCCAACTGCTTTGCTACAGCCTCGGTCCCTGCA

>TDF42-4-1

TCCCGATCTAAAATGTACCACAAACGTGGGCTTTGGGCAATCAAAGCCAAACATGGCGGCAAGTTCCCAACTCATGCTCCTAAAACTGTTGAATCGAAAACTTCCGATAAACCCGCGAAGTTTTACCCAGCTGATGATG

>TDF42-4-2

TCCCAGATCAGCAGAGATTGATCTTTGCTGGTAAGCAGCTCGAGGATGGGCGTACCCTTGCTGACTATAACATCCAGAAGGAGTCCACCCTCCACTTGGTGCTTCGTTTGAGGGGCGGTATGCAGATCTTTGTCAAGACCTTGACTGGAAAGACTATCACTTTGGAGGTAGAGAGCTCGGACACCATTGACAATG

>TDF42-4-3

TCTACGCCTTGCTAGCATGGCAGCTGCAGAAAAGCAGCCAAATGCTATAGCACAACCCATAAAAGCGGCAACCAAAATGCTTGAATCAACTTGAATAGCCAAGTCAACCACAGGACCAATAGATGCTCCTTCCAGTGCAGCCGCAGCCATCAACAGCCCAACTCTTTTTTGCTCCTGATATGAAGGGATG

>TDF42-5-1

GAAGAAGGCTCCTAACGCAATCAAGGAAATTCGCAAGTTTGCTGAGAAGGCAATGGGGACCAAGGATGTAAGAGTTGATGTTAAGCTCAAC

>TDF42-6-2

CAACGCTGGCATTTCAGGGGACCTAACTCCTACGTTACTAGAGACAACTAGTGA

>TDF42-6-3

GACGGTGATGATTTTGGTGGAGAGGAATTCGCCGACAGAAATCAAGAAAGCAGGCGTACAAGGAACTCAACTGCTGCTGTTAAGGGCTGTTTTTCGGG

>TDF42-6-4

CATGAGTGTAACCATCTGTAGTGTCCTTATAAATAACATCAGCAGAACGAGCTAAGTTGA

>TDF43-1-4

CCAACGGTAGATAGGAACCGAACTGTCTCACGACGTTCTAAACCCAACTCACGTACCACTTGAATCGGCGAACAACCGAACCCTTGGGACCTTCTTCAACCCCAGGATGTGATGAGTCGACATCGAGGTGCCAAACGACTCCGTCGATAAGAGCTCTTGGGAGTCATCAGCCTGTTATCCCCGGCGTACCTTTGATCCGTTGAGCGAGAGCCCTTCCACACGGGACTCCCGGATCACTATGGCCGACTTTCGTCTCTGTTCGACCAGTCGGTCTCACAGTCAGGCAGGCTTATACCATTACGCTCACGAGCAGAATAGACGCTTGAGCCTACCTTCGCACACCTCCGTTACTCTTTAGGAGGCATCCGCCCCAGATAAACTACCCACCTCGCAGTGTCCCGCCTTCCCCCGAATGATCGGTGCGGCGGTTAGGCATCCTTAGACGAAAGAGTGGTCTTTCAGGATTGGTCGTTCCTCCCACCTATCCTACACATTCGATCAAGGTTGTCACTGCGAAGCTATAGTAAAGGTGCACGGGGTCTTACCGTCTAGCCGTTGGTACTCCGCATCTTCACG

>TDF43-2-2.2

GAAAGTGTGCGGCACCATGAGCAATCATACAATCTCTTTCCATCTCTCCAAAACGAAGACCACCATCACGAGATCGTCCTTCGGCAGGCTGCCTGGTGA

>TDF43-2-3.2

GAGACTGCGAGTAAACTTCCAAGATCGTGGAATTCTCAACAAAACCCAGAAAAAAAATGGCCTCAAACGTTCATGGGTTCCTC

>TDF43-3-2

GACGAGAAGTGTAAGTCGCCGTTGGTGACCATTGCTGTGGAATGACATCTCTAGTTGAGA

>TDF44-1-4

ACGGCTACACAATCATGTGGAGAACTTCACTTTCTCTCTCTTCAAAGAGACTCAATCATCTCTCATCAAGCCCTAACCCTAATTTCAACCTAATCTCTTCATTTCACAAACGATCTCCAATTTTCGACGCCTACGGTG

>TDF44-1-5

CGCTCTAATTGCAGATCAGATGTACCAACGTACCGCCCAGTTGGGTCAATGCCATGTTCATCACACACAACCTCCCAAAATTTAGAACCAATCTGGTTACCACATTGTCCACCCTGGATGTG

>TDF44-2-1

GATTAGAAAGGAACGAATCTGATGCCTGTAGGTTTTATGATTTTCAAGATGATCAGTCTTTTGATGATTCCGTGGAATTGTCATCTGAGTATGGTTCTAATCGGTATGACACTATAGATGCTGTG

>TDF44-4-2

CTCCTTATGGTCGTTCCTTCTTTGCCCGATCTACTGGCCGTCTTTCCGATGGCCGTCTCATCATTGATTTTCTTTGCCAAAGTTTGAATGCAAGATTATTGAGCCCATACATGGATTCGTTGGAAGCAAACTTCCAAAATGGT

>TDF44-4-5

CCCATTCTGTCAAAATACCAGTCCCCAACAGCTAATGCCACAGGCTTGTTTTGAAGAACAGGGGAATCATCGGCAAACCATGTATCCTGCCTCTCAGATTGGCAGTGAGCAAAACATGCATTTAGGAATAACCCATTTTGCTTGGACCTT

>TDF44-5-2

TCGCGACACTTTTCGACGCTTTTATCTGGTGCTTCATCCATCATCGGAGCTTCCTGCGATGGA

>TDF44-6-3

GAGAGAGAAAATGGGGCTGACATTCACGAAGCTGTTCAGCCGTCTGTTTGCGAAGAAGGAGAT

>TDF44-6-4

TGGAGCACTGCGAACCAAACACAATTTCTTCACAGATCCTAAGCTTTTCCTCAAGTGCTGCAAATGGTCAGACAGATCATACGCAGCACCACGTTGCTCCATTTGTATCATCACAAGATGCTCAAGGAAGCTGTTACAAC

>TDF54-1

TCAACTGCTGGAGTATGGATTCTATCATGCAGATCCTCATCCTGGAAATCTTCTTCGTACATATGATGGAAAATTAGCTTATTTAGACTTTGGCATGATGGGT

>TDF54-2

TCCTCCTGCCATGACTTTACTTTCAGGTGCACCTCGGAAATTTACCGTGACCCTGACTGTACGTTCAGT

>TDF54-3

TCGTGTTTGGTGCAATATAGAAATGCAGGTGACGTTGGCCTCAGATTGACAATTGCAGAAATTTAGAAGCTGGCCAGGGCAGTTGGATTGGTAGTTGACGTGGT

>TDF55-1

TCCATGTTTGTCACAGTAAGCTGTAACTGTGATCAGTTTCTGGTTTTGTAAACTGCAGTATCGTGTACCATGCGAAATCTTATGCTGATTTTGTATTTTCAATGAGGTGTTTGT

>TDF55-2

ACCGCTGATTTATCCTTTTTCCGAGGAGAGTTTCTACCTGCTTTCCACCATCACCAACAACAGCAGCAACGAATGTTTGCTCGTAAAGTTGATTCTGATTCTGATGATTCTGATGATTTTGATGATGATGATGATTATGTTGATGATGTTACTCAATCAGATAGTGA

>TDF56-1

TCAAGAATGAGGAAGCGGAGGAGGCAACATTTGACGAAGATTAGTATGAACCTGATGATTCGAACCACTACTCATGGGAGCACTACTAGTCTGAGTATTGATAATAATAAAAGGATGACTCAGTAACTTGATTGCAGTCCATCTTTTGGCCGGCTCTCTCTGCAAACAGCAACGAATAAAGTCCCTAAACTCCCGGGAAGCCGTAGGAGGCGCTTCGGGTGGT

>TDF56-5

ACCTTCACAACTCACTGGATGGACCGCCAATGGCGGTGATCCGTGTGGTCAGTCATGGAAAGGTGTTACTTGCTCAGGCTCAAGAGTAACCCAGATAAAGATATCGAATCTTGGACTTACCGGTTCAATAGGTTTCCAGCTTGAAAGCTTATCATCCTTGACTGAATTTGACATAAGCCACAACAATTTTGGAGGCACCGTACCCTATAACCTTCCTCAAAATTTGCAACGA

>TDF57-1

ACGCTTCACCATCGAAAGCAGATCATCAACATACAACAATTGAAGAATCTTTTGATACACCGCAACAAATACTAACCCTAAGTCATTGTGGAAAGTCCACTTCAGGGTATAAGATGCACCAGGAGCGTCATAATTGTATGATGATAAACCAGATCTTTCTTCTAACAAACAAGATCGAATCAACGCATCGATTGGCGAGCCTTTCAATGCATTGCTAAGCTCATTACAAGTCCAAAGGA

>TDF58-1-2

ACCCTTGATAATATCAGCAGGCCTCGATTAGTAAACATGTGCAAATACATGGGAATCAGTCCTTTTGGTACAGATACATATTTGCGTTACATGCTTCGTAAGAGACTGAAGCGGA

>TDF58-2-2

TCGAGCCATCATCACCTTGTTCATTGCAAAATGGGAATACAAGCACACAATCCAAGCAACAGACAGGTTACGGAGTGAATGCACGGTTTGAGCTCCTCCGTGAGGTTTGGTCTTTGTAAGATCATGCTGATGGCATTTCGTTTTTCCCCTTCCCCCTTTTTTTTGTAGTTTTTATACGTAATGTAAGGATACAAGCATGGGGAAAAAAATATATATACATATGGTCCATATATGAATCTGCGAAAACTTGTGTATGTTTCATTTAGGATGTTTACCTGTGCTGTGT

>TDF84-2

CTTGACCTATTTGCCATGATAGGATCGCAAACCCAACAGCAATAACTGCACCTATATTGGCCTTCTCAGGTGT

>TDF84-3

CAATTCACCAAGTTCTGGAGTCATGGATATTCTGCGCAAACCAATTACTGTGTCGTATAGGAGAGAGAATCAAGAGGGAG

>TDF85-1

CTTCAACCATGGTGCTCGAGGCGACAATGATCTGCATCGACAATTCTGAGTGGATGCGTAATGGCGATTACTCTCCTAGTAGATTTCAAGCTCTTTCTGATGCTG

>TDF85-3

CAACCAAAGCAGTAACAGAGTACAAGATCCAGTAAAAGATAAGTGCAGCAGCAATCAAAAGAGCGTTACGCTGAGACTTCATGATAGATTTCTGGTGACGAAG

>TDF86-3

CAGTAAACGTTGTATCAATTTTTTTTCAAAAATGCAGCTTCAGAAGTGGGGTGTAATGGAAACATCAATTCTCATAGCAAG

>TDF87-1

CATCAGGAACCATTCCTCCAGTGTATCGCAAAGTGTACTTCTCTCTCACGTAATATTGGATCAACTTGTCATAGTCAGGGTTGTCGAAAGTGGCTCTCAAATTTCCAGGGCAGAACAATTTCCCTTCAGAAATTTCTGTTGTTTCCTTCACATGTAGCCACTTTCCTTCATCAAGAAGAAG

>TDF87-4

CATGTACGGGAACGAGTTTGGGCTTGGGAAACCAGTAGCTGTTCGCAGTGGGTATGCCAATAAATTTACAGGGACAACGACATCTTATCAAGGATATGAGGGCAGAGGTAGTATGGATCTGGAAATATGTTTACCTCCGGACGCAATGGAAGCTCTTGAATCGGATGAG

>TDF88-1

CAACACTAACGTAGACAAAAGTGTAGCAGCAATCCATGCCACAGTTTTCAGCTGTTTACGAAGGCGTGAGGCTGGAGAGCTTCTATATGACCATACGACTAACACTGTAAATGCTGTTTACGAG

>TDF88-2

CTGCAAAAACAAGGGTGTTTATAAATGCTAAGGCTATAGGAACAGACCCTAACGTTTGGCACACGATTGACCCGGATGTGTTCGAGCCTGAGAGGTTTGTCGATAGCTCGATTGACTATAAAGGGCATGATTTCGAGCTGATCCCGTTTGGGATTGGTAGAAGAGGGTGCCCTGGGGTTGGTTTCGCGGTTTTG

>TDF89-1

CATTTTGCAGAATCATTATCCTGAAAGACTTGGTTTAGCAGTCCTTTATAATCCACCGAGAATCTTTCAGGCATTTTGGAGGGCTGTAAAATATTTCCTGGATCCTAAGACATTCCAGAAGGTGAAGTTCGTTTACCCTAAGAATAAGGACAGTGTAGAACTGATGAGGTCCCATTTTGATATGGGAAATCTTCCAAAGGAGTTTGGTGGGGATGCCACATTAGATTATGACCATGAG

>TDF89-4

CTGCTGCACTTGCTGATTGTGTTGATCCACCCAAATTCGTGCTCGAAGCGATTTCGGAGGTTTTTCCTGTTGATAAAAGGGCGGATAAGACTGAGAGAGTGAATGATTTGGGTTGGGCTTGTGTTTTGCTCCTCGAATCGCTGATTCCGGTAGTGGTTGATCCGGTTATTGGCAAAAAGCGCTTGATTGTTACCCCAAGTGT

>TDF90-1

CAACACCCATTTTCTTTCACCTGTTTCTGCGGCATTTCGTCGAACAGTTGGTGGGCATAGATGTATTTCACCTCCTAGATCCGTTATTTCGTCATTCGGGTCGCCGGAGCTCCGGCGCCCGACTGATAGGTTGTTTCCTGGAAATGGGTTTTCTTCAAATTCAGTAATTTTAGCTCCAACTACTTCAATTTCTGTCAATCAAAGGACTGAAGAATCAGCTTCTGAGCTTGAGCTGTTTCTTGAGCTGGTGCCTTTTAGGATGAG

>TDF91-1

CACCGGTAGCAAACACCGAATCATGACCTGTAGCTAATACTTCTAACAGTCGAGCACCTCCATAGCAGATTCCTATTATGCCAAGCTTTTTACTTGATTCAGTAGTCATGAACTTATCAACCATCCATTGGGTTGACGCTGCAAGGTCTTTTGCTACCCTTATTGAATTTTGTTCAGCTAGCCACTGATCAAGCTCAGATCGTGGTCGATCAATCGACCAAGGATTGCCACGGAAAAGATCTGGCATCAAGACATTGTAGCCAATGCAAGCAACGCGGTCAGCAAAATTTCTGTTGTAG

>TDF91-2

CTGGAGCAGGTGGCCTTGATGCAATATTTACAATAGCATTAGGTGATTCAGGCAGCAAAGTTGCAAAAGCATGGAGTTCATTCAATGTTCTGGCGCTCCCAATTAGAGAAGACCCTCATGGTGTGAGTCTGGAAAATGGTGACCCACGTGTACGCATGATCACTAATGGCATTTCTACTACAAGTATCAACTAGCTCTTACTTTCGGTTCAGCAGAATCTTTGTATTTGTGGTGCTATGTGGTGGAGGTGATTGCTCTTTGATTCTAGCGATTGAAAACTTCAACTTGTACATCTAGTATGCCTTCAGGTTACATGATTTGTTCTTTTATTGGTG

>TDF91-3

CTTACTAAACTCCCTTCCTCTTCTAATGCAGCCGTAAAGCGGCGCCACTCAAGCGCAGGAGTGCGCTGTAAGGCTACCGCAGGAGCAGCAGCAACAGAAACTGCTGCTGCTCCGAAGCAGCCGAGAAGCCGCAGTAAGTATGAGATACAAACACTTACTAACTGGTTGCTGAAGCAGGAACAAGTAGGGGTTATTGATGCTGAGCTTACAATAGTATTGTCTAGTATATCAATGGCTTGTAAGCAAATTGGTGCTTTAGTTCAAAGAGCTGGTATTTCTAATCTTACTGGTGTTCAAGGTGCTG

>TDF100-1

ACCCCATATTCGATCAATGCTCTTGTTCTTCTAACATTCGGAGCAGGAGTATTAGCAC

>TDF100-2

ACGAACACGACGAACCATATCAGCAGGTGTTAGATTCTTAGACTTTGCCTTAGAC

>TDF101-1

GTGGGCTTGACGACGAAGAAGTTGGGCTACATAGACGTTGGTGAGCCCAGTTTCTGAAGCAATTTGAGAGTATGATTTGTTGGCGCTTTTTTTGGCTTCTTGAAGTCTTTCTACTACTGATAATGT

>TDF102-3

GTGACATGCGGTTACGCGAATCACTAACTGGAAAAGTAGATGATGCCCAACCAAGAAAGCCCTGAAGACATGCACTTGATGAGGTCCTGAAACGGCTCTCAGCCACTTGTTGAGAGGGCTTGATTTGGTTTTCTTCTGAAGGCGTTGTTACAGGGGGCAGTACATGTG

>TDF103-1

CACCAGCCAATAGAACCACTGTTGTGGTACCGTCACCAACCTCGGAATCTTGAGAACGAGCAATATCAACGAGGATCTTAGCAGCAGGATGAACAACATCGAGGAGTTTCATAATAGTAGCACCATCATTAGAAATGGTGGTGTTACCTTTATCATCATGAATAAGTTTGTCCATACCACGTGGGCCCAGGGTTGTACGGACAACATCGGCGACTCCAGTACACGCGTTGATGTTGCTGATTAGTTGGGCCCTACCTTGTGATGTGTCTGTTCCTTCTTTGAGGAGGATGATCTGTGGCTGCATCATCGCCGACATTGTTGGATGGTAAAACCCTAGGTTTTGTTGCAGAGAGTGAC

>TDF104-1

GTACAGGGGTCCACATGCCTACCACGGGCCTATAGTGGGCTGTAAGAGTGGGTGTGCAGCCTTCAACACTGATAAGCTTTGTTGTAGGAATAATTACAACAGTAAAGAAAATTGTCATGCGTCAAATTACTCAGAGTTCTTCAAGCATTCTTGTCCTCATACTTTCACTTATGCTCATGACTCCTCTCTCATGCATGATTGTGCTGCTCCACGCGAGCTCAAAGTCATCTTCTGCCACTAATAGACTAATTATCCCCACTTTTGTAGTTTGTACTTTTGTTGTTTTTCTTCCTTTCTTTGTAATGTGT

>TDF105-3

GTCCCGCAACGAGCGCAACCCTCGTGTTTAGTTGCCAACGTTGAGTTTGGAACCCTGAACAGACTGCCGGTGATAAGCCGGAGGAAGGTGAGGATGACGTCAAGTCATCATGCCCCTTACGCCCTGGGCGACACACGTGCTACAATGGCCGGGACAAAGGGTCGCGATCCCGCGAGGGTGAGCTAACCCCAAAAACCCGTCCTCAGTTCGGATTGCAGGCTGCAACTCGCCTGCATGAAGCCGGAATCGCTAGTAATCGCCGGTCAGCCATACGGCGGT

>TDF106-3

GTGACAGGATCGAAGTCGTTGAACAACAAAAAGAGTGCCCTGAGGAGCTCAAGGAGGCAATATCGAGCCTGATATATGCAGCATCAAGGTGTGGT

>TDF107-2

ACCTGAAAGGGCATGTTCCTTGGCAAAACAAGCTTTTGATGAGGCTATCTCTGAGTTGGATAGTCTAGGCGAGGATTCTTACAAAGACAGCACATTGATCAGCAGCTTCTTGCTTCTCATCACCAGAC

>TDF108-3

ACAGAACCAATACTTTTTACGGGATCATTGACCTCTGAACTTGCATCTCGCTTACTTGTGCTTTGATCTGGATGATCAGTTTTCTTTTGCATGGGACCTGATACCTTGGAGGACTTCCTAAATGTTTCAGCACTGCAC

>TDF110-3

ACAGCAGCATAATGAATGAATAACTTTATTGTGCCCATCAAGAAGGGCTTCCTCCGGAAAGCCATATTGGAAGTTCATAAAATGAACGGAGCATTTCCATGCCGGTGTCCTACTACCGAAGTATAGCAATTATGGCTTATGAAACAGGACCAGCACTTCTCTTGCAAC

>TDF110-5

GTCAACAAAAAACCAAAACTCAAATAACTTAGATTCAGAAGTCTGTCTATGTATAAAATGAAATAACCAAAGACAGCAGCAGTAAGGCAAGTGCAACTCCAAGAAAATTATTTACTCAACCATTGAAAGAGGTGAGGTTGAATGATGAGTGTTTCTTGTGAGAACTACAAAGTGATCATTTGCTTGATAAAGAAATCAATTCTATCCCATTATGAATAACTCCTACATCTTACTCTGCATGTCAATTCAAAAGTACAAGGATCCAGACTCGTCTTCTAAAAAAACACAGAATCCTGGTGT

>TDF110-6

GTCTGGGTTTTCTGACACTGATTCGAACTCGAGCTCGAGCTTATTTTCGGACTGTGATTCCGATTCAAAGAAGGAGTTCTCTGTTTCGGAGCTACCTGACATAAACAACGGAGTATACGGCAGTGATGACTTTAGGATGTATTGTTTCAAGATCAAGCCATGCTCAAGAGCGTACACTCATGATTGGACAGAGTGTCCATTCGCGCACCCGGGTGAAAATGCAAGGAGAAGAGACCCACAGAAGTATCAATACACATGTGTGCCATGTCCTGAGTTCAAGAAAGGTAGTTGCAAGAAAGGAGAAGAGTGT

>TDF110-7

GTTCACAAATCAATTTGTCTCTTGGCTCACCCGCCTTACGCTTAGTTCGCTTCTCTTTACGTGTTGCAATAAACATTTCAGCAAGTGTGGGATTTTTTGTTGTTTTCTTCTCCTTTTTCCAATCTTCTCTAACCCTAGCAAAACTTTTCGGTCCTGTTGTATGCATAATGTTCTTCTTCTTTCCCTCAGTTTGATTCGTTGTATTTTTCTCACTAATTTTCTTATGTTCGTCGCTGTGCCAGATTTTCAATAGTTCCTTGAACATTGTTTTTGGGACCCTTCTTGGTCGATTTTTCATCCTCATTGCATCACTTGT

>TDF112-4

GTCGTCTGCAAAGGATTCAACCCGCCGCTCGGTAGGAATTGTACTTCAAGGCGGCCCTCACGACGCATCCGGCGTGAGGGCTTAGCCTACGACACGTGCCCTTGGGGGCCGAAGCCCCTACTGTAGGACGGCAATCGGGCGGCGGGCGCATGCGTCGCTTCTGGCCCGGATTCTGACTTAGAGGCGTTCAGTCATAATCCAGCGCACGGTAGCTTCGCGCCACTGGCTTTTCAACCAAGCGCGATGACCAATTGTGCGAATCAACGGTTCCTCTCGTACTAGGTCGAATTACCATTGCGACACTGTCATCAGTAGGGTAAAACTAACCAGTCTCACGACGGTCTAAACCCAGCTCACGTTCCCTATTGGTGGGTGAACAATCCAACACTTGGT

>TDF113-1

GTTCCAACGCGATGTAGGAACTTGTAAGTCATTGATGATACATGGAAGCAAAATTGAGGCATAGCTGATGATCTCTTGCATCTCCCCAACCACCCCAACCTTTGAATCCAGCTTTATACCCTGCTTGATGGTCATAAAAAAACACACTCCACTGTGGGCTAATGTTCACAACTCGGTCATCATTCTGAACTCCAATATTCATAGAACCATTGTCTATACAAGCATTTGAATCCCCTTGGCCTTGATGCAGCCCACATTTCCCAAAGTGAAGAGCACTACTCGTAGGTCCTCGTAATGTATATACAGGACTCCCAAATGAAGGGTAAACCTGAGACCACATAGTTATATCCCAATTGTACTCGTCAAAGAAACAAAACTCTTCAGCTTTGTTGT

>TDF114-1

CTTGCTTAGCAAACTCTCTAGCTGCATACCCAGCAGAAACACCACCACCAATGATGACGTAC

>TDF114-2

GTTGCGGCGGAGGATCTGGAGAAAGAGAAAGGGAAACAACGTTCATCATCATCATCAAACAG

>TDF116-1

CTCACTATCATCTAATCGAAGCAACTGAGGCAGCAAAGCCAGTTTTAGGTAAATATTACCGCGAACCAAGGAAATCTGGACCTCTGCCTCTATAC

>TDF116-3

GTACGCGCAAAGATAATAGTTGCAGTGACTGAAAGTAGGAAACTTGCTCTTGCAGGCTGCAGCTCTATTCTTCACATCTACTTGGTAG

>TDF119-3

GTCTCTATTTGACAAAACAGTGAATGAGTTTGATAGTTTCCAAGATGTACTAGACGCTTTTGAAGGAGGAATCTTGATACCAGATAGTGAGCTCTTCAAGAAAATGCGAGAAAGCATTCCGCTTGAATTAGTGAAAGCTTTCCTACAAAGTGATAATGCACCACTTCTAAAATATCCTATGCCTGATGTTATCAAAGAGGATAAATTAGCTTGGAGGACTGATGAG

>TDF121-1

CTACTGCTGTTAGCTTGTATGGCTTCTGTTATTGTGCTCATCCTGTTTTCCCCACCCTATACAACTCCATGAAGAAAAAACAAAACTTCTACAAGGTACTACTACTTTGTTTCGTCTGCATGACTATCAGCTATGCTTCCATTGCAATATTTGGGTAC

>TDF121-2

GTATAAACAAAATCCAACTACAAAACAAGATGATGACATACAGCTGAATCTCACACTCAGACACGTTGTCAAAATACAGTAAACAAAGTGCAAGATAGACTGCAGAGCTGTAGATATATATGGCTCGATCTGAGACTCTTACTAAATGTTCATAGCAAGTACACATGATTCAGATACAGTAAACAACTACAAAATGGTAGAATGCAGAGCCATAGATACACTTCGAATCCAATGCCAGG

>TDF122-2

GTGATGAGTCCTGAGTAAGTGGAGTTAGTGCCAGACATCTTCCACTATTATGTATTCATTGTGCGAAATGGACTCAAACAGCAGCAG

>TDF126-2

CTTCAATGGATGAAGCAACTTCGGATCATCAAAGACTATTAGATAGATTGCAGTTGTATGATTTGGTTGAGCGAAAGGTCCAGGGTGATGGTAATTGCCAGTTTCGTGCTTTGTCTGATCAATTCTATCGTACTCCAGAACACCACAAGTTTGTGAGACAACAAGTTGTTACTCAGCTCAAGTCAGAACCAGATTATTATGAGGGTTATGTACCTATGGAGTATGGGGAATAC

>TDF126-3

CTTCAAATAACAAGTCCAATACTAAGCAAATGACGAAACTAGCTTATGGTTGCGATTCTAAAAGGAAGCATAATGTTTCGCCAGTAACAATATAAGTTCAAAGCGATTATACCATTATCAATGCTGTACTTCAATAAGAAACTGAACAATACTATTACCTCGGCACCTCCGTTGTTTTTAGTAGCAACAATTGAGTATATTTTGCGAGACATAACATATTCTATTGTTGCAAC

>TDF127-1

CTGGGTGGTCCAGTTACAGTTACACTGGTCTCTGTCCAGGTAACCTTGCAACCCATCTTCTCTAGAACCTCCGCGAACTTTACGTCACCCTGCAAACTGCTTGTTCCACAACCCTCAACAGTCACAGTCCCACCAGTGATTGCCGCTCCTGCTAAAAAGTAACTAGCACTTGAAGCATCACCCTCAACATATGCATGTCCAGGAGATTGAGCTTTTCAGCTTTCTGCTCGAC

>TDF127-2

GTCAGCCGTATAAACGAGGCTAAGCTCAGTTACATTTACGATGAGTCCTGAGTAAGAAGGTTGTTTTTGGTGTTTTTGATGATGCTGTAATTGGGGTTGCCATTGAAACTGCTGATAATGAAGCCATTATTAGTTTTTTCTTTAACTGGAGGGGAGGTTGATGAGTACTTGGACCTATTTGAGTATAATTCAGGTGCAGATAATCAGTTCTGCGAGCAGTACAACCAGCAGCAG

>TDF128-1

GTCTACAACACCACACATGTTTTGCAAGACTCTTACTGCCTCAGACACCAGCACCCATGGTGGATTTTCTGTCCCTCGCCGCGCCGCCGAGGACTGCTTCCCTCCACTGGATTATAAGCAGCAGAGGCCATCTCAGGAGCTTGTTGCAAAAGATCTTCATGGATTAGAGTGGAGGTTTAGGCACATTTACAGAGGTCAGCCGCGCAGGCATTTGCTCACAACAGGGTGGAGTGCTTTTGTGAACAAAAAGAAGCTGGTGTCCGGAGATGCAGTGCTATTCCTCAGAGGTGCTGATGGAGAGTTGAGGCTAGGAATTGGTACGCAGTCAATGATGAGTCCTGAGTAAGTTTCAGAGAAATTGAATTGGGAAATTAGGGTTTTGGGGTGATTGATTTTTGTGGTGTAAACTTTGAATTGTTTGCGAGGAGTTGTAACGGAG

>TDF128-2

GTGCTGCAAGAACCTCTGTTGTTCTAGGCATTTTTTCCTTGTTCTAGGAGTTTTTTCCTTGTTCTAGGAATTGGTACGCAGTCAGT

>TDF130-1

GTGGACCTTTTCCGGTAACAGCAGCCTGAACAGCAAATCCAAGGAAGGCAACCATAGCTAGTCTTGCATGCTTGATTTCTGCCAACTGAAGGGTTGCTTTCTTCTCGGGGTCGGATGCCAAACCCAATGGGTCGAATGTTCCGCCTGGGTAAAGCCTCTTTTCCGGGTCAAGCTCAGCATTTCTTTGGAATTTCATAGCAACTTTGAAGGGGTTGATGGCAGCTAGATCACTTATACCGGCCCTATCGATATGTTGTTTACCGGCCACGTCTGAGATACCACGGAATATTATACATGGCACTCCATTCGTTAGCGATGCATGAACAACTGCTGCACCCTCTTCATCAACGGTGGAAACTCCATGTTGTTTGACAAG

>TDF129-3

GTCGATGACACAACACAACGTTGGAGCATTGTTGGTTGTGAAGCCTGGAGAACAAGAATCTGTTACTGGTATTATCACAGAAAGAGATTATCTGAGGAAAATCATAGTGCAAGGAAGATCCTCAAAATCCACCAAGGTCGGCGACATCATGACTGAGGAGAACAAGCTTATTACTGTTACTCCCGATACCAAGGTTCTAAAGGCAATGCAACTTATGACAGATAACCGTATTCGACACATCCCGGTGGTTGAGGGGAAAGCAATGAAAGGTATGGTGTCAATTGGAGATGTTGTTCGTGCAGTGGTTAGTGAACACCGTGAG

>TDF130-2

GTGATAAACATTTGTCAGCTTCAAGTTTAGTAATCTCAAATAACTTATCCTTGATGACAACTTGTTCGCTTAGTTTGTTGGATTGATCCATTAGAAAATCCAAGATAGAAGCAGTGGTAATCTTATTGTTTGGGGAAGGTTGACATCGCCATCTCCATTCCAGCTTCACACCTTGCTTCTCAAATGTCCAACACAGCCTTCGGTGGCCATTTCCAGCATCGGTAAATCGGTAAACGGAATGTGGTTGCTGAATCCCTAAATAGCTTTCTGCAAATTGGATGTATTCCCAAACTGATTCATCCCACTGACTTGCCTTCTCTCTCACATCTTCCTCAGATGCATTGCAAACCCAG

>TDF132-1

CTCTCTGAGTACTTCATCCTACCTTCCTCACCCTTACCGATGGCCCTCATTCTCTTTCTCTGATCAGGGGTAAGATCCGGCAAAGAGTGTTCAATAAAGCGAGTTGCTGCTTGGGTATTCACAGTTACACTAGGTTTCAAGGGTGCTTTACCCACTTCGACAAAGCACTGTAGCTTTTCTTGGTAACGTCCTAACCTCTCAAGCTCTCCT

>TDF132-2

CTCTCATGGCGCGAATCTGCATCTCAAGCGCCGGAATATAATCCGTCGCTTCTTCTAGAATCACCGGAAGAGCTTCTTTACGGCAACCGGGAACTAACCGTCCAAGCGTCTTTACTTTCCGTTGAACCGCCGGTAAACTTGTGCCTTTTCCT

>TDF132-3

CTGCACCAGAGAAAATGGAACCCCTAGGTATCATTTCCGCTGCACCTTGGGGATCTGCTCTAATTCTAACTCTTCCATTTTCATCCACATAACCTGAATTAGTGTGGATTTCTGTAAAATACTCTTTCATACCATGATGTTCCAATATTGTTTCAATGAAGAACCTATTTGCATCACTTAGTATCCTCAAATCACACCCCGCTTCATAAGCAGCT

>TDF133-3

CTGACTGCGTACCAATTCCTCAAGAAGATCTCCTTCAATGATCTTGAACCTTATTGATTTCTTCTCTTCGTCTATCGCTTCAACTAACTCTTTCGCTACACATTTCTTCCCATCAATAGTATAATTCCAGCAAATTACAGAACCAGGCTTGCCAAATTCACCCTCATGCACATCACACGCATGAACTTTATCAGGGGTAATATTGGCAACTTCATGTGGTTTCTTCCCAAACAATTCATGAAAGAGATCACCATGTGACTTGATATCAATCTCAACATCTAGCTTTCCT

>TDF134-2

AGAGAAGAAAGCCTTGATCGAGAGGAACGAAGAATGCCCCGATACGCTAATTGTATTGCAACACCATCCTGTCTATACTCTGGGTACTGGTAGCTCTGAG

>TDF135-2

AGGGCCGACAGCTACGTATAAATATAGTGCCTCTCCCTCCGAAGGCCTGGCTAATATGGGTAAACTAGCCAGGTGTCCTTTGAGGGCCTCGAATGCAAGTTTTTGCTCTTCTCCCCAAACAAGATCTTTCTGGAGCTTCAGCGCTTTGAAGAAAGGTAAGCCTTTTTCCGATGCCTTTGCAATGAATTGGGAAAGCGCAGCCATTCGTCCAGTCAGCCGTTGAATGTCTCTTTTTGTTTTCGGCTTGGGGAGGTTGAGTGCCGCTTGCACTTTATCGGGATTTGCATCAATTCCCCTTTGGCTTACGAGGAAACCGAG

>TDF135-3

CTTTCTCTCTACCGATTCCACTCATTTTGTACCCACCAAAAGGAATTGCAGCATCAAACACGTCGTAACAGTTTATCCACACTGAACCAGCT

>TDF136-1

CTCTAGAAATGGTCTATGTTGGGAAAAGCAATGCCAAGGAGCGAATGAGGAAAATCACAGCAATCATAAGCAAAGAAAGGCTTAGTCATTTTTGGCCCGACATAACCTCAATGTGGTACTTTTGGACTAGGCTCGAGTGCATGCTTTACTCAAAGATGCAACATGGGAAAAACGTGGAGAGTGACCTGATCATGCAAGAAGTAATGACAGTGCTTAGCTTTGATGGAAGTGACCAAGGGTGGGCCACTATGTGGTTCGGATCAATCGAGATGACAAGAGCTAAAGGGGACACGATACTAGAGAGTCTTCGAATGTTTCCAGATTGGGAAGAAAATGCAAGGGAAAAGGGATTTGTGCCTGCT

>TDF136-3

CTTCTCCAAAATGTAGTTGACAGCATCTTCCACGGTATCTACAACCAAGTCTGCAGGATACTCGCTGTGATTTCCGTTACCTTGGAACATACCTGTCCTGGTTAGTACAGGTATCCAAGGATGTCCAGCCTGTCTTGCACCT

>TDF146-1

CTTCACGTAGTCAAATATCTTTTGCTCTAACAACCAATCACCTTCTTTTGCTTTGGTGAATGTTGTGTAATTTGTGA

>TDF147-1

CTAAACACCCTTCGAATCCCGCCACTCTCCCTTGATCTCCCACCTCTAGAACCCCAAGAAATCCTCCTTGGAACACCGCCTTGA

>TDF147-2

TCTGCCTGCTTTTACTGTGAATCCTGTCAGAGATGCATATATGAAGATAGGGATTCAACATACCGCATCGTGTAG

>TDF149-1

CTGCAGCTGTTGTTGACTGAATAAATGAAGAAATCCTGTTTCCGGGCAAGCAACAAGCTCAATCAGCTCATCATGCCTCTCTGGAACCTCATCCAACTCCACTAATGAAACAGACACCTGCATGCAGCGGAGGA

>TDF150-1

TCACCATCTCAAAAGCTGTTCTTACATTGGTCGAATCTTTTGCAGATGTCTCCATGAAGAAAAGCCCGTGCTCTTCTGCAAGGCTCTTGCCTTCATCGACACTCACTGCCCTTATATTGTCCAAGTCACACTTGTTTCCCACGAGCATTCTAGCTACTGTTGTATCAGAATGAGCTGCACAG

>TDF152-1

TCACATATTCCTACACCAGAACAAAAAGATACAAAAAGAAAACAATTCCAGTAGATACTCTTCCCATGTCCCCTGAAAGCAAAGGCAATATTCAAAACATGGATGCCGTCAATTCTCGGATCTCTGATGAAACTTGCTCAGATTCAGAAACAAGTTTTTCTTGGACATCTTGGCTTAGCTCCCCTTTTGATGGCTTGCTTAGTACCAAAACACAGCATGTTGGTCGCTTTGTAGCTCCAGCACTTGCAAGATCTTCTTTAGAAGGAACATATACGTAAG

>TDF152-2

CTGTAGAAGAGAATACAATGGACTCAACTCCTCCAGCTGTGGTTCTTTCAGAGCGAAGAAAGGCTCTTTTTGAACCACTAGAACCTATTGATAATGTCTCCAGAAAACGACCCTCAAATGAGAGTTTACTTCCTCCTCCAGACTTTGACTGTACAGCTTATCCAAAGGGCTGGGTGATAGGTAAGAAGAGGAAGTTGGTGAATGTTGACGTTGTTGAGAGCATGCGAAGGATTGCCGTCCAGGAAATGAGTCGCAAGGATCGTGAAATTGATGGA

>TDF153-1

CTGATGCATAAGCATCTCCTGCTCCACAGGTGTCGATTGGCACACAAGGTGATGGAGGGATGAATATAGCTTCCCCTTTGACCCCAATGTAGGATCCTCGGACCCCATCTGTCACGGATACAAGTGGGACAAAATGGCTTAGGTACCTTGTAGCGGAAAGTGGGCTTTCCTTCAATGAGAAATTACAAAGAGCTTTCGCTTCATCACTATTTGCAAAAATAATATCTGCATAATTGCCTATTATTTCCCAGAAATCATCATAATGCCTCTCAATGCAGGACACATCTGATGCGGTAACTGCAACAAGAGCTCCGCTGCTGTGTGCATCTTTGCAAGCTTTTGTAATTGCTTTGATAGTATCAGGAAGTTCAAATAAATAGCCTTCAACCACAAGGATATTTGTCTTAGAGA

>TDF154-3

TCCTGAGAATGCTGCTTTGCTCAAACAGATCTTTGGTTTGGAGGTTCTTCCTTCAATCCAATACAATGTACTTCATATGCTATTTCAGGGTTGGCGGATCCTCAAATTGCTAGCAGTGCATTTACCTTTGGAACTGCAGCTGTTCTTCCGTTTTACACTTTCATGGTTGTCGCTCCAAAAGCTGAACTTACTAAGAAGGCAATGAGAAGTAGCATACCGTACTTAGTGCTTGGAGTTCTGTATGCATATCTGTTGTATCTTTCATGGACGCCTGAAACCATACGCTTGATGTTTGCTAGTAAATACTGGTTACCAGAGCTGCAAGGTATAGCGAAAATGTTTTCAAGTGAGATGACTTTAGCATCTGCATGGATTCATCTGTTAGTAGTGGACCTCTTTGCTGCTAGGCAAATATATGAAGATGGTCTGGAGCTGGAAATCGAAACACGACATTCTGTTTCAATGTGCTTGCTTTTCTGTCCCATAGG

>TDF155-1

CTGACACTATAGTGTGGTATGCAAATGCTGGTAATCCTGTTCCACAAGGCTCATCGGTAAAAATAACTGCTGATGAAGGCCTAGTTCTTAGTGACCCCCAAGGAAATAACTTGTGGAATGTTTCAAACGAGTTAGTTAGCAATGGTGGGGTGAGTTATGGCTTCATGAATGATACTGGAAATTTTGTCCTCAAAAATAGCACCAGCAACAATCCAGTTTGGCAGAGTTTTGATCATCCCACAGACACCTTGCTGCCTGGTCAGTCTTTAGGGA

>TDF155-2

CTCGGCGGCGGAGGAAGCGGAGTGTTATACACGTGCTGCTGACGTGTACCAAGGGGCCCACCAAGGATATTCCTAACAGGGAGAAGAAAGTAAAACTCCTTATCACCGATTTGAAGAAGATCCTGTGAATCTAACTTTACAGGTGCATTTCCAGGAAGATGAAGAACTCCTTCAACAAGGCAGCCATTTTTGCCAAGAACTTCTAGGGCAAAACGACGTCGTTGAAAATCGTAGAAGATTCGAGCGTGATGACGTGAGATGTTCATTCCACCGCCTAATGAGGCTAAATCGACATCAACCGTTGATTTTTTTGAGTTTCTTCCGAGGATTATTGAGTAAGTTTGCATGTAATATTCAAAGTCTTCGCCTTGAAGCTTCGCGAAACCTGCTTCTACGTCGCTGCCACCACCTCCGCCGCCTGTGGTTGCGACGGCGTGCGCCATTTGTTAGGGA

>TDF157-1

CTCACTTCCCAAACATGCCCCTACGCTTCCTCCTTTGCATCCAGATCCAGTACTGAAGAACCCTATCATGGATGATTCCAGTTCTGCTCCTGCATCTACCGCTGAGATTGTTGCTAGGA

>TDF157-2

TCCAGGGTCATTCCGTGCTTGGAAAGAAAATCATCAACAGTGCAATGAGTTGGTTCAGACTATGAG

>TDF158-1

CTCTAGAAAATCCAGCAAGCCACAATTTCTGTGCTACTGCAGAAATAGTAAGGACTGGAAGAGTAGCTAAAGCCACCAGGGCTAAACGCCACTGTAACAACATTCCAATAAGAAGAGCAACAATTACAGCAGCACTGTCTTGTATAAAGATCGAAAGACGGTTACTAAAAGCAGCACGCACAAATGTCGCATCATTTGCCAATCGCATTGATAAGGTGTCAGCGCTGTTTTCCTCATCATCAAACCACCCAACTTCATTTCGGAGCATAGCTGAAAACATCATCCTCCTTACTCGTTCAGTCATTTTCTCCCCCATAATTCCAAAATAAAAATGTTGCAAGAAGTTGGCCACCACTGTCACAATGCCCATGCATGCAATGA

>TDF158-2-1

TCTTCAACCTTCAATCTTCAACCCTACTTTCGATGAATCACAACTCGCGACCTAGGTATCCACCAGGGATCGGCAACGGCCGTGGCGGCGGCGGCGGCGGTAGTAACGGAGGTTACGGACATCAAAACCCTAATTTTCAAAATCGACCTCCTTATCACCAAAATCACAATCAAAATCATCACAATAATCAACATCAGTATGTTCCGAG

>TDF158-3-1

CTGTGCCGAGTATTGGGAAGTTTAGTGAGGAGTATAGGTTGTCGAATGCGTTTGCTAACACGTTTACTAGGCATTCGGGGATATTCTATTTGTCGTTGAAAGGTGGGATTACTCAGGACTCATCAC

>TDF158-3-2

AGGACTGCGTACCAATTCCTCGTTGACTGCGTACCAATTCCTCTCCGAGGGCAATTCCATCAACTTCTTGGGAGATCCTCTTGTCATAGAAAGCTCGAAGTTTTTGGTCATCATCAATCTCGAGTTTTTTCTGGCACCCTGTAGTAGGGTTCGCGAGATTGATCTTCATGA

>TDF164-2

AGGAGCAACTTCATGGACGAAGCACAGAGGGTATTCAAATGATGCTTCTGTGCCAGAGAATGAGCAGCCCATATCTGAACCACCTCCAATCAATTGGGTTGGCTGTGACAAGTGTCAGAAATGGC

>TDF164-3

GCTAACTGCAGACAGTCAGACACTACTCCACATCAATACTAAAGATCTTGCTGCTACTACTAGCTTTCGGAATTGTAATATACAGCACGCCGTCCTTCACTTCAGCT

>TDF166

AGCTGCACGAATGCACATTTATGTAAGGAGAGGGGGTCCAAATTACCAGAAGGGTTTAGCTAAGATGCGGTTGCTAGGGGAGGAGATTGGTATCCCAATTGAGGTTTGCGGTCCTGAGGCAACTATGACTGCAATTTGCAAACAGGCCATCCAATGTATTGACGTAGCTGCATAATTGGTTCAGGGCAGTTCACTGGATGTGTTGCTGCTGTGTTTCTCTTTCGCTCATAGTTTGTTGATTTACATTTGATTCAAGCCCATTGCTCCACAGTGCCAGCTTCCAGCAATTTAGATCTAGTGCG

>TDF169-1

AGTCTGCAGGGAAGAATCTAATCTGACGATTTCTTCCATCAATTCCCATGCTTTATCAAAGTATCCCATTCTTGTAAGAATGTGTAGTGTCTTTTCAATTGCATCTGTAGTGGGAGGTGAGTTGGAATAGCAAAGGGTATATTTGAAGAACTCAAAAGCTTTGACACCATTTGTACTAGCTCCAAATAGGCGACCTAGAACTTTCTCCGC

>TDF169-2

AGGCCATGCAAGGTCTGGATCATAGTTGCCGAAGTCGGGGAAACTAATCTCATTGCAGAACCGGTAAAATCCAATCCATTGGTCCATATTTATAACCTTGTAATCAGTCTGAGCTTTCAAGTATTCAATAAAGTAGTCGACTTGGGCACGGAACTGGGATCCATACAAGTTTCGACCATCAAGTGCTAACAATTCTCCGTATTTTACCGC

>TDF173-1

GCCTGCAAGGTACCCAGTGAACATCAAGACAGAGAAGAGGAAAACCAAGGGAAAACCATACTGGGCGCCATTTCTCTGCTGTTCATCTG

>TDF184-1

GATGATTCACCATTCCCCAAACAACACTCAACTTATCAAAAAGTATCTCCAAAGGATCCACCAACTTCTCCCATTTCGCTTCCACATTCTTCTCCAACTCATCCAAATCACTCTCCAACTGTTTCAAAACAGCGC

>TDF185-1

GACCAGCAATTGAAGATATATTGATTACTGACCCGCCTACTTTTGCATCACGCATGCGTATGCACACAGATTTTGACACCAGCCATGCTCCAGTCAGATTTGTTCTCAACGTATTACCCCATTCCTCCTCAGATAAATCTAATGATGTTAGCACGTTACCGC

>TDF186-1

GACGTCGCTAATGGCTAAGATCTGGTCCGATCCTTCTAAAAGGGGCTCCGTTTCTTGTGCTAATAGAGC

>TDF188-2

GACGGTGAGGTGACGATGAGAGAGACGAAGATGAGTGACGTGTAAAGGAGGAGATCCAACGGAGGAGGTGAATTGAGAAGAAATCGACGGTTCAGATGAAGGCGCATTAGCATTTTCCGATGATTGATTTGAGAATACGTTGAATCGGCGAATTGGAAGACGGAGATTAGTCGCCGGAGAAGAAGGTAAGGTTAGGTTTCCGGC

>TDF189-4

GAGGATCCCTGTTAGAGACAACCTTGATAGGCTTGCCATCTACGGTAAATGTCTCATTATCGACAATTTTGATATCGGCCTTGAAGGTGCCTAATATCGAATCATATTTGAGCAAGTGTGTTGCATTCTTGACACCTCCGCTGTCATTCACAACAACAACTTCAATAGGGGAGTTCTTGCGGCCATGCCAGCACCTAAGGAAGTTCCTTCCAATACGTCCAAATCCATTGATTGCCACCTTCAACTTGGCCACAGTTTCCCCCCTTACAGGAGCTGAACCTGTCGGATTTGTTGTTAGCTGAGCAGCAATAACATCATGAAATGATGCCTCTCGTGAAAACGTCACGCTATTGCCAGACCGCAACCCAGC

>TDF192-3

GAGTCTTGTGACCGAAGAGTTGAAGCAGTACTTCAGGCCTGAGTTCTTGAACAGATTAGATGAGATGATCGTGTTCAGGCAGCTTACAAAGTTGGAGGTCAAGGAAATTGCCGATATTATGTTGAAGGAAGTGTTTGAGAGGTTGAAGCTCAAGGAAATTGAACTTCAAGTAACAGAGAGATTCAGAGACAGGGTTGTGGATGAAGGCTATAATCCAAGCTATGGTGCTAGGCCTTTGAGAAGGGCTATTATGAGACTCTTGGAAGATAGCATGGCTGAGAAGATGCTTGCTGGGGAAATCAAAGAAGGTGATTCTGTAATTGTGGATGTGGACTCAGAAGGGGCTACTGTTGTTCTCAACGGTAGCAATGGTGCTCCTCCTGAGAGTTTACCAGAGGTCATCACTGTGTAGACCATACACACAGTACACACCTTGGTGGCTTGGTTGCTGGGC

>TDF194-3

ATTGCGCTCCATCAATCCCAGAAAAGAACATAACATGTCTTACCAGGCTTGACCACAACAGGGCTCTTGGTCAGATCTCAGAGAGACTGAGTGTTCAAGTATCTGATGTGAAGAATGTGATCATCTGGGGAAATCACTCATCAAGCCAGTACCCTGATGTAAACCATGCAACTGTGAACCCAGAGAATGGTGAGAAGTCTGTCCGTGAGCTTGTTGCTGATGACGCATGGTTGAACGGAGATTTCATAACTACTGTGCAACAACGTGGTGCTGCCATCATCAAAGCAAGGAAGCTCTCCAGTGCATTGTCAGCTGCTAGTTCAGCCTGTGATCACATCCGTGACTGGGTTCTTGGAACCCCTGAGGGCACCTGGGTTTCTATGGGTGTCTACTCTGATGGTTCATACAACGCCCCAGCTGGAGTCATTTACTCTTTCCCAGTGACTTGTAAGGACGGAGAATGGAAAATTGTCCAAGGCCTCCCAATCGATGACCTTTCAAGGCAGAAAATGGATGCAACTGGAGCAGAGCTTGTTGACGAGAAGGCTTTGGCCTACTCATGCCTGTC

>TDF195-1

GCCCCGGGATTGACATATTGCAATTATTTTCTTCCCGGCCATGACCTATCCCTTTGAATTGGGA

>TDF196-2

TCAATGTTGTGACTTGAAACATCTCGCCTGCTCCCTCACAATCACTAGCGGTGATGATGGGTGTATGTAGGTACAGAAAACCATGCTCTTGGAAGAAGACATGAGTGGCATAAGAAAGTTCATTGC

>TDF197-1

TCGTCCGTTTCATGAGAGAGGTGGTAGTTTATCTGGTGACGTATCGGTTGATTTGATTCGACCCGAGGTGATTGCTGATCTTAGAGATATTGCTGATAGAATGATACGGGCCGGGTATGAGAAGGAGTGTTATCAGGTTTATAGTAGTGTGAGGCGAGAGGCGTTGGAGGAGAGTTTGGC

>TDF198-2

TCATGGCAACAATTTCTGAATCATGTTCATGGATAGCTGTGTCCTTCGAAGCTGCAGTGCTTTCATCCAAAAACAATGGCTGACCTGATGTATAGAAATCCATAACTGCACCAAATATCTCTGGTTTCAGAATATCCCATGAAGCATCATCTGACTTTGTCACGGTTATGAAATCTGATCCAAAAAACACTCTTGTAACTCCATCAATTCCAAAAAGCGACCTTGCTAGAGGC

>TDF199-1

GCTCAGCTTGCTAAGGAAATCAAGAGTAATGGGAGGTGCCCTGAGAAGATGTGGCCTGGGATTAGTAGAAAGACTCAGCTGGCTTTAGATGCAATTGGAAAATCTATTGAAATTGGATACAAACCTGTGCATATGTAGTATGATATGCACCAGAACGAAAAGGCAACAATTTACTTATGCTTCATAAAATTACAACAAAATACCATCATTTCATTGAAGATGTAGGTAATGTCAAATATTGTGTGA

>TDF199-3

GCGGTGTTTTTCGACGTCGTATTTTTCGTACTTGTTGCAGATTGTATCAACTCTTGTGATTATGTCGA

>TDF200-4

GCTGATACACTTGTAAATTTCATGGAGAAGTGTTACCCCGGCGAAGATGATTTGGCTATAGCCTGTGCTGTTCTAACGTATCTGGCTTCTGGCAATCTGAGAGATGCAAACTATGTCATGGATGAGGTCAAGAAGCAGTTGGATGCTAAAGAAGAAGAGTTTCCTGAGTCAGATCTTATGCAGTTTGTCAGTTATCTCTTGCAAACGTTGGAAAGGGATGCTTTGCCTCTCTTCAGAATGTTGAGACAGAATTACAGATCTAGCATTGATAGGGATTCTGGA

>TDF201-1

TCCAACTCCTTCTTACTCTGCTTCTGCTCCTCCCCTTTCTTCTTCCTTCTCCCCACCTCCTCCTCCTCCTTCTGATTTCTCTAATTATTACGTTTCCCCTGATTACCCTCCTCCATACCCTCATAATTCCGATTTTTCCATCAATTCTACCCCTACTGCTCCTTCCTTCCCTCCTAACCCTAACCCTAATCCCCAATTCCCAAATTACCCCTACGATCTCCCTCAAAATCAGACTTCCTACTATGGTTATGATCATAATCATACCAATCAAGCTGCCCCGAACCCGAATTTGAGTTTGAACAACACTTTTTCGGGTTCGCAGTACTCGGCTTACGAAGACGGTACTCGTTGGGGTGATTACGGGGAACAGGACAATAGTGTGTATGAAGGTGCGTATAGGTATGAGGGAGGGAAGGTGAAGCCGTATGGGGCGAGAGGGACGGCGGGGTTTTCTGGCGC

>TDF202-1

GCCCATCGAAGGTGGTTATTACGAAGCTGAGATTGGACAAGGATAGGAAGTCGCTGCTTGATAGGAAGGCTAAGGGAAGAGCTGCTGCTGATAAGGACAAGGGTGCCAAGTTTACTGCTGAGGATATTATGCAGAGTATCGA

>TDF205-1

TCAACGCTCTTGGTGGTCGAGGAAAAGACCTCATGTAGAACAAGATATATGCTCCTTCTGACATCACCTGGCTCATGGACACTGGCTGCACCTGTTGACAATCACCATGATCAGGACTGGAAACAGAATTGAATTACAAAGGCACCTAGGATTTTCAGAAACAACAGAGACTGCTCAAAACTATAAACATTTTTCTAGAATATACTCCTTGCCACGGCATTCAAACAGGTACTCTGATTATCTCATTGATAGTCTACATTGC

>TDF205-2

GCGACATTTTAGCTCAAAGGGAAACGGATGATGAGTCAGGTGAGATTGTGTTTCGAGCTGAGGAATTGGACAAAATGGTGTATGTACAAGCCGCGTTATCTGAGTCACTGAGACTTTACCCGTCTGTGCCAATTGATTTTAAGAATGGGAGAGAGTCATTGAAACGGTTTAGTAGGCTTGTTGCATCACAACATATGCATTTGCTTGTTGTACTCTTGTTTCCCGCAAGATTACTGCTGATAGGCGCATACAGCGTGA

>TDF206-3

TCCAGACACTCAAGATATCTTCACTAAACCTGATGCTAAGAACTACTCCGCATATATTATCCCCATAGTCAAGCTGATCACCTACCAAGGCCAAAACCAGGTCTTCCCAGAAGCGCCCTGAGACAGCCTTCTTGAATCGGATGATCCACTTGCCACCATTGCAATTCGCAGAATCCTCCCATAATGGGC

>TDF207-1

TCCAAGGGAAAAGGGCAAGAGTTGTAGCAACATCATCTGTTACTGGACAACTCAGTACTATGGAAGATAACCTTTCAACAAAAAATTTGGAGATGGATGCAGTTCTTGGGAGGATTGCTTCAACTGCACAGGAGTTGGCTCATTATCATTCTGGAGATGATGATGGCATATATTTGGCTTACTCTGATTTTCATCAGTACCTGATTAGTGATTCAGCTTGTACAAAGGAATTGAGCCAGTGGTTTTCCAAGCATTTGGACTCGGTTCCATTTCGGTTAGTTGCTCAAGATGGGAAATCTAAATGCTCGTGGGTGAGTTTGGATGACATTTCAAATGTGTTAGTTCGAGATTTAGAAAATTGCCATCATCAACGAGTGTCAGAGCTTCAAAGACTTCTTTGGCAGTAAATCTTCCAAAACTGGTTCGAGTCCAGGATACTCTTCAGCTATGCTCTGGC

>TDF209-1

TCGACGAAATGCTTCGGGGAGTTGAAAATAAGCAGAGATCCGGAGATTCCCGAATAGGTCAACCTTTCGAACTTCTGCTGAATCCATGGGCAGGCAAGAGACGACCTGGCGAACTGAAACATCTTAGTAGCCAGAGGAAAAGAAAGCAAAAGCGATTCCCGTAGTAGCGGCGAGCGAAATGGGAGCAGCCTAAACCGTGAAAACGGGGTTGTGGGAGAGCAATACAAGCGTCGTGCTGCTAGGCGAATCTGTGGAGTGCCGTACCCTAGATGGTGAAAGTCCAGTAGCCGAGAGCATCACTAGCTTATGCTCTGCCCCGAGTAGCATGGGGCACGTGGAATCCCGTGTGAATCAGCAAGGACCACCTTGCAAGGCTAAATACTCCTGGGTGACCGATAGCGAAGTAGTACCGTGAGGGAAGGGTGAAAAGAACCCCCATCGGGGAGTGAAATAGAACATGAAACCGTAAGCTCTCACCACTACTACTACCACCACTACTTCGTTGGC

>TDF210-1

CCTCAGGTAGCCCATGGTACGGCCCAGACCGTGTTAAGTACTTGGGCCCATTTTCAGGTGAGCCCCCAAGCTACCTTACTGG

>TDF211-2

GTTCAGTAGATCGGATAACCTAATCGACGGCACAGAATGCGCCGTTTGTCTTAGC

>TDF212-1

GTGTGGTAATGTCGCGAGGTCTAGGTGTCCTTACCAGTCTTGCAAAAGTTGCTGTGCTAAAGCCCAGAATCCTTGTCATATTCATGTTTTGAAGGC

>TDF213-3

GTATACAAAAAGAGGAAGCTAAGAAGAAAGACGTTCTTGTTGAGGAAAATGTGCCTGCCCCATCTCCACGGTCGAATTGTTCGTTGACCATGAATCCGTTGAAAGAAACCGAGTTGAGCCTTTATGGTGGC

>TDF215-1

GCGGATTGTTTACAAGTCCATTTCTCAGGCCTATTTGAAAATATGGAAAACAAGCATATCGTGCTTCACATGATATACAAGTAATCCATGATTGTCGTCACAAAAGGGTGGAAAAAAAAGGAAAACATTTGACCAAAATTTTGCCCATATGCAAGTCCACATTGGGAAGCTTTCAAGCGAAGGGTCATTCTTCATCATCATAATTGAAC

>TDF215-2

GTGAGACACTTGAAATGTTGGAAAAAAAAGAGCAAGTGCTTTTGAAGAAAGCTGCTGCCGAAGTTGAAAAGGCTAAGGAGTTTACAAGAGCAAAGAACAAGCGTGCTGCTATCACTTGTTTGAAGAGGAAGAGGCTATATGAACAACAAGTAGAGCAGCTTGGAAATATGCAGTTGC

>TDF216-2

GTGGTTATGATTGCGAACATAACAGCAAAAGCTTCAGTGCAAACTTGGGGGATTTCAATTACCTTTATAATGAGTGGAAAAATTGGCTTGAGAAGGCCATCGCCAGCAAGCATCCAATAACTTGCTAGAGTTGCAGTTATTGTTTCCTAGTGCAGTAGTGTAGCCTGTACATGAACCTAAACTCCTGCTGCAGCTTGAGGCTCAGGATCCTTTTTATCCTTTTGTGGTCCATTGC

>TDF217-2

GTTGGTATCCAAAGGCAGTCATAAAAATTGGTATGGTGGTAAAAAGACTGAAGATTTTTCTTTCAGTAGAAAAATCAGGCAGCAGGCTCATCTTGTGTGTTTTTCCTTCCATCATTGCTTCTATTGCCAGTACTGATGATATGACAATAAACACCAAGGCAAGGAGTATAGCCAGTAATGAAGTGTGCCGAAGTGAATCTACACGACGAAACAACACCAATGGGAGGATAACAAAAAGCACAACAAAGAGAATCGCAAAGGCACGC

>TDF220-1

GTGGCACACTGGACAAAGGCATCACTATCTTTCGAGATTCGTCTCTAACATTTCATGCATTTTCTGATGCGGATTGGGCAGGTAATAAAGATGATTATACTTCTACCATGGATCAAGTTGTGTTCCTTGGATGTATTCCATTACTTGGAGCTCCAAAAAGCAAAGGTCTCTTGCTCGATCATCTACTGAAGCTGAATATCGAGCTGTAGCCTCCACCACCCACCACTACAGAACTAATGTGGGTTCGAAATTTGCTAAGTGAACCAGGAGTCCGACCTTTTCATACTCCAGCCATCTATTGTGACAATCTTGGTGCTACTAATCTTAGTGCAAATCCCGTATTCCACTCCAAGATGAAACATTTAGCTCTTGCATATTATTTCATTCGTGAACAAGTGCACAATGGCACCTTGCAAGTTACTAATGTCTCTACTAAAGATCAACTAGC

>TDF221-3

GCAAATTACTGCCAAAGCAGCATAAACTCATCCCAAGTTTATTTCTAATTTGGTGAAAGAAATTACATATAGCATGGGTGATGTAAACAAATGGGTGTCCACTTCAATATTACAATCGCTTGAATCACTGCCTGAGCCTCCCTCAAACTGCTGTATCTTTGTAGTTCCTGAAAATCTTCGCAAGGTAAATGAGGATGCTTATCGGCCCTTGCTTGTTTCGATTGGTCCTTTCTACTGCAATGATACAAGGCTAGCGGCTATGCGACAGCAGAAATTGAGGTAC

>TDF221-4

GTGTGGATTTGCTGCAGAAAATTTTCCGACGTCGTCCTTCCTTTGTGTGGTGGGAAAGCTGATGCTACGGTACGAGGAATCATTCATGGAACAAGAATTGAAGGACATTGTTGTTGGAGAGGCTTGTGC

>TDF223-1

GTGAGATTGCTAGGCTTCCATAGAAAATTGAAGGATTGACCTAAAACATATGAATCTCATTACAGGAAGTCTACATTCACTATGAATCATGCTGTTGCTTTGTGGGACAATTTGTGCCTTCTAGACCTCTTAGTAAGGGAAGGGGGCCATGATCAGGGGAAGC

>TDF223-2

GTCCCGCAACGAGCGCAACCCTAACAATGAAGTTACTGTTCGACAGCTTGCTGAGGTGGTGACCAAGGTTTACTCCAAGGTCAGTGGTGAGTCAGAAATCGAAACACCCACTATTGATGTTAGCTCCAAAGAGTTCTATGGTGAAGGATATGACGACAGTGACAAGC

>TDF231-2

GCTAGGATCAGGCCTCCAAAGCTCCAAGATTGCTTTATGAACTAACCCTTCCAGCTTTCTGTCCTGAGCTGCAGATACTTCATAGAACTGAACAGTCAACTTGTGGTGACTGAATGGATAGTCTCCTTTGGCTTCACTTACCCCAGCCCATCTATAATCTGGATACAAAGTAGCATAC

>TDF231-4

GTGGCTGGCAATTTCACAGGTTTTTGATGACAGAGTGAGAACCCAGCATAGTTCCTAGTTTTGATCCATAATTTTAGCTAGTTATAGGTGCTCCCTGCTCTGCTACTGCAGACTTCTTTGGCAGTAAATCTTCCAAAACTGGTTCGAGTCCAGGATACTCTTCAGCTATGCTCTGGC

>TDF233-1

AGCGGGTCTTTCATAAGGAGCAACCGATTCTTCAGAGATGATACAAAGTTCACCAGGAGAAACACCTCTGTTGGA

>TDF233-3

AGACGCAATCTGCTCGATGTATGGATTGTGGTACTCCATTTTGCCATCAGGA

>TDF234-2

AGCAAGGAACAAGGTGTAGGGCTGTGACTATAGCTGTTGTGCTGCATTCTGATGGCAGCATCGCTGAGTATTCTGTAGA

>TDF235-2

AGAGCCATTCAAATCTGAAGTTCTTGCGAGGGAGAAGGTTCATTACGCCATCTCCAAATTCGAAGATGGCAAACCTCTGAAATCAACCATCCGAACTGATGTTGTAATTGATCCTGCTCAGGACTAAGTAGGTCTACTGTGCCGTCTTTTCGA

>TDF235-3

TCCACAAAGGCATCAATCTTATCATCATACTTTTCATACAAGACTGGTAGTGTGTGTGCAGCAACAAACCCAATGTAGACAAGTGTTAGAAAATTGCACCAGCCTCCAATCAACGCAGCAGCAATCAATCCTGATATGACCATGACGAAGTGCT

>TDF236-1

TCTCAGGCGTAGCAGGATTGTCAAGAGGAAACCAACCGCTGTAACCTGCATCTGCTCTAGCAGCTGTAATAGCGTCTTTGATAGCAAACAAGACGGAAGATGCAAGAAAGAATGGTGGCTCACCAACAGCTTTCGAAGAATGTATGGCT

>TDF236-2

TCCTGCCAGTCCACAAGGGAGGGGTGCACATGGGAGTCATCAGAGACCTCAGGGAAGGGGTTTTATGGGCCATCCAGCACAAGCGCCCCCCCCATTTGTCCCACCATCTCCCATGAGACCTTATGGAAATGCTGTGGTGTATCCAGATCCTGCTTTGGTCTATTATTACCAACCTTTGCCGGCGGCTTTTCTCACTGGTCCACCACCTCCACCTTTTCTTGCTCCACCAGATGCCCTGCGTGTATCT

>TDF236-3

AGCAAGACCTCAACCACACAAACCAATACATTGTGGGTCTTGCTCTTTGTGCTCTTGGTAACATATGTTCTGCTGAAATGGCTCGTGATCTTGCACCAGAAGTAGAGAGACTGCTTCAGTTTCGGGATCCAAATGTTCGCAAAAAAGCAGCACTATGCTCGATTCGGATTATCAAGAAATTCCCTGACCTGGCGGA

>TDF237-1

TCAAAACTTCTGTCGCTCCATCCCCGCAGGGGCAGAGAACCCGTCGCTGCCTCGGCTGTGCTATCGGCGGCTCTGGGGAAGTCGGAATAGGAGAGCACTCATCTTGGGGTGGGCTTACTACTTAGATGCTTTCAGCAGTTATCCGCTCCGCACTTGGCTACCCAGCGTTTACCGTGGGCACGATAACTGGTACACCAGAGGTGCGTCCTTCCCAGTCCTCTCGTACTAGGGAAAGGTCCTCTCAATGCTCTAACGCCCACACCGGATATGGACCGAACTGTCTCACGACGTTCTGAACCCAGCTCACGTACCGCT

>TDF237-2

AGACATGATAGCTGAAGCAATCAAGAATCAAACAAGTGTCAGTGGAAAGGAGGATCATCGTTACGTGAAGCCTTATACCAAGAGGATTGATAAGTTGCGCATGCCAGCCGGTTATCAACCACCGAAGTTCAATCAATTTGATGGTAGGGGCAACCCTAAGCAACATGTCGCACACTTCGTTGAGACGTGTAACAACGCTGGTACTACCAATGACTTGTTAGTCAGACAATTCGTTCGTTCTGTCAAAGGTATTGCCTTTGATTGGTATACTGATCTTTCCCCCCAATCAATTGATAGTTGGGAGCAAATGGAGGGA

>TDF240-2

CTCGTTGACTGCGTACCAATTCTCAGGCTGGTGATTGTGTGGACATTGGTTTACTGAACTTGAGTGCAATACTGAAAGAAGTGGGCCTGGAAAGGTTGACAAGCTTTCCTGTTGTGCACTGTCCCGAGTCTTTTGGTGTCATTTTGAAAGCAAACGAGAGATTCAACAATGCTGGAGCT

>TDF241-1

TCAGTACTCATCCTTTACTCAGGACTCATCCTTTACTCAGGACTCATCACTGACTGCGTACCAATTCTCCCAAATAGCGGATCCAGCACTCCAAGGCAGATACCCGCCAAGGGGACCGTATCAAGCACTAGCAGTTGCTGCAATGTGCGTGCAAGAACAACCTAATATGAGACCCCTCATGGCAGATGTTGTTACAGCT

>TDF241-2

AAGGGTAAATGTTACAGTAGCCGGTAGCTTCACCGGCCTCAAGAATTTTTTGCGTAATCAACCAAGGACTCTAGTGAAGTAGCACAATATTTTTGTTCTCCCTCTATGCCCTTACGCTCACACGCTTTGATTGTCTCCTTGATAGTCTCGGCCTCTTCAGAGTTAGGCTTGACGGA

>TDF241-3

AGATGGTTCTTGGAGATGGCTTAGGTTATGGTCCTCAGTTGTCTGAACATATTATATCGGATGCGGGTTTGACACCAAGTATGA

>TDF247-1

CAGCAGACTTGAAGAGCCTTGGCGCTAAAGCCGAACAGCTTTCATATTCACATAAATATTCTTCAAGGA

>TDF248-3

CATAAAGGAGCATGTCCTTGTTCCTGAGCATCAATTGCTAACTGACGAAGAAAAGAAGACACTGCTTCAAAGGTACACCGTGAAGGAAACTCAGCTACCGA

>TDF249-2

CATCAAATTGGTACTTCTTGAACAGCTTCCTCTGCCTTGAAAGAAACAGTGTCTTCATCAGGTCTTGGTATGTGGGATCTTTTGAGGTAAACATGAAGTAAGCATAACCAATCACTAAGCCAGTGGCTGTTGAAAAAAATGCGATCGGCTCCATCACATCCCACGA

>TDF250-1

TCGACAGTAAGAGCTAAGACGTAATCGAGCTTGTTTTGGCCATCATCCAAGAGACCATATCGGTTCATCCTCCTCAGAAGAGCTTCACCTTCAAAGATACGACGAGGGTTCTTCTCATCAAGAGTAAGCAGATGCCTAGCAGCATTACGGATACGACTCAAAACAAACTGAACTCTCCAGAGCTCCCTCTTGTTACGAAGCCCATACTCTCCAACAAGCTTCAACTCTGCATCCAATCGTTCTTTCTCAAATGGACGACGTGGCTTG

>TDF250-2

CAGCAAACAGCTCCGACACGCCTTTAGGTTTTACTCACTGCAGTGGAAGACATGGGGTGCTTGTTTCATACAGGCAGCGTGGCGGCGTCACTGCAGAAGGAAGCTTGAGAAAAGTTTACAAGAGGCTGAACAATGTGCTTTGGGGGAGGGTGGAAGTTCACCTAGTTTGGGTGCTACTATTTATGCATCAAGGTTTGCTACCAATGCACTACGTAACTTGCGACGTAATAGTCCCCAAACTACGA

>TDF250-3

CATCGGCATCTTCCATGGCCTTGACCAAAGTTTTCTTTCCTGGTGTCTTCTCAATATCAATCTTGATATCAGTCAAAGAAAGCCTCTTCAAGTTCATCTGGCCTCTAACCATGTCAGGTGCATCGACTAGAACTCTGTTCATGTCGAGGACACCGACGATGACAACTAGACGGCCATAATCTTTGCCATAGTTGACTAGAGCAACTCTTCCGATCTCAACGTACCCTTGAACGGCATTTTCGA

>TDF251-1

CACGCTTGTAAGATAACCATTAGTTAGGCCCAACAGACAAGTCAGCACACTCACAGGAATTTCAGTTCGGAAGAATTGGGGACCATGCAAGCATCCCCAATAAAGAGGAATAAACAGCAATCGAGCAAAACATGCTGTGACGGCAGCAGTTTCATTTTCAACAAGATAGATGGAAGTCAAACACTTCCCCACTAAATCAAATACATTGTAGCTCGTGATTAGGA

>TDF252-2

TCCATCTGGAACAAATGAAACAACTGAACAGAACAAACAATATCAAACTCATTCTTCCCATGATTCGAACTCCGAAGAGCAGAAAGAGGACTCATCCAATGCACTGAATTTGGAGAAGAAAGAGGAAACATCAAAAGCTGAGGAGAACCAAAACGCTGAGAAACATGAGCAGCAGCAGCAGCAACAACAGGAGGAATCGTCAAACTCTAATGAAAACTCTAACAACTCAGAGTCAGAAGTCAATAAAAAGGACTCTACTAAAGATGAATCCCACCATGAAGCTGATACTTCAGTCGCTCAAGATG

>TDF254-2

CACCACGAATCTTCTTGGCCCTGTTCTTCCTTTCCTTCAATTGTTTTCTGGACTTCTCAACCTTAGTGGCCAAACCATTCCTGATCAGCCTGTATTTGGGTTCATACTTCTTCGCACTTTCAACAGAATCGTAAATCAGACCGAATCCAGTTGATTTACCTCCACCGAAATGAGTACGGAACTTGAAGACAAAGATGGAGTTTGGATCTCTCACATCATACATGGTAGCAAGTTTATCCTTCAACTCAGCCTTAGAAACATTAGCGCGACCTGGATGAAGAACATCAATAACGAATTGCTTGCGAGCAAGAAGACGGTTAGTCATGAACTTGCGAGTTCTGAGAGTGACAGCTTTGTCCGCCATTGTTGATTGATTTTTCTTTCTTGA

>TDF254-3

TCATGAAGGATGTGGGATTGATGGGGAGCTTTTTCCAGTCCATGAACAAGTCATGACTTCTTTGCTCCCTGAACAATGTCGGCTTATTGGTCGTCCTCCCAGACGGCAGATATGAGGCTATTGGCTAGTGTAGATCTGTTTCTCTTTCTTTACAGCTGGGGTTCATGGGTTCCCTCTCCTGTGTGTGGTAGTAGAATGTGCAGAACATAGATGACATAAAAGACATTTTAGTTTTTCTTGTGATTCCTCTTGCTATTTTGTCATTGCAATGTAAGAGCGGCCTGTAAATTTTGTCAGATAATGACCTGTTGTTGCTCTCTTCATTCTTTTTTGATATGACATTCCTCTTTGTTTGTTGATGTGTGCAGGCTG

>TDF255-2

CAACCGCCGCCGTCGCCATGGATGAGAACTCAAAAACCGTCGATGGTGGCGCTGCTGCTAAAACATCCGCCGATGATTCCGCCACCACTAACCCTAACCCTAACCCTAATTCCGCCGTCACCACCACTAACTCCGACGGTGATGGTGGATCCGACGGCAGTGGGACCCTCCCTCAGTCTTCTAGAACCCCCTTCACCAACCTCAGTCAGGTTGATGCTGACCTCGCTCTTGCTCGCACCTTACAAGAGCAGGAAAGGGCATACATGATGCTAAGTATGAACGGTGGTCGTGGGGTTGGTTTTGGAAGGTGGGAGACAGGGATTTATATCCCTGATGATGACGATGATGACTTTGGGGACTCCCATGACCATGAGTATGATGATGAAGACGAGGATGAGGATGATGATGACGGGGATAATGGA

>TDF259-4

GAGCCAAAAGACACCCCTCTATGTCAGTTGCCTCCCGTGTAATATCAGCATTTTTGCCACGGA

>TDF260-1

GAGGCATCAGAGACAGTGGGTATGATGTCCTGGGCTTGTCTACTACAGTAGGAGACGCTTTTTTCAAGTGTTTGGACATTGATTCTGTAACTCTAGCTCTGGCGGA

>TDF261-1

AGACCGCGAAAACTGTTGCATCACCTGGCCGAGGAATTTTGGCTATGGATGAGTCGAATGCAACCTGTGGGAAACGTCTTGCCTCAATTGGGCTAGAGAACACTGAAGCTAACCGACAAGCTTACCGTACCCTTCTGATCTCTGCCCCAGGCCTTGGTCAGTATATCTCTGGTGCTATCCTCTTCGAGGAGACTCTTTACCAGTCCACCACTGAAGGCAAGAAGATGGTTGATGTCCTTGTTGAACAAGGCATTGTTCCTGGCATCAAAGTTGACAAGGGTTTGGTACCACTTGCTGGTTCAAACGATGAGTCATGGTGCCAAGGTCTTGATGGTCTTGCTTCCCGTTCTGCTGCTTACTACCAGCAGGGTGCCCGCTTTGCCAAATGGCGTACTGTTGTGAGCATTCCCAACGGTCCATCCGCCCTTGCTGTGAAGGAAGCAGCTTGGGGTCTTGCCCGCTATGCTGCTATAGCCCAAGACAATGGGTTGGTTCCAATAGTCGAACCAGAGATCCTGCTTGATGGTGAGCATGGAATCGACAGGACATTCGAGGTTGCTCAGAAGGTCTGGGCAGAGGTGTTCTACTACATGGCAGAGAACAATGTGATGTTTGAGGGTATCCTTC

>TDF263-3

GACCATGGGCAAAGTACAAGAACGTTGGGTCGGTAGCACCATCCTTGTAGTAAGCAAGGACAATTGCACCGTCATCATGCATACTCTCACCAACAAAGAATTGAAGGTCCTTGAGCTTGGACATGAGATATTTGGTTGCGGCCTCAACATTCTTTTTAAGAACAACAATGGAGTTGGCATTCTGCTCTTGTCTGACATCTATGGGTTCGA

>TDF264-3

TCCATTTTCACAACAATGGCGATATCTCTCCATTCAACTGGTTCAACCTCATGTCTCCACACTAGAGCTCGAGTTCCAACACCTTCTCTCAGTTGTGCAACCAAAGTTTTCGTCGGA

>TDF265-1

TCCAAGTTTAGCAGACTCAGCAGCCAAAGCAAGACCAGCAGGACCGCAACCAACAACCACCATGTCAAGCAATTCATGTCCATCTGTTATTGGAGGCAGCTTATCAGAAATAGTAGAGGGA

>TDF266-3

TCAGAGCCTTATGATGTCAGTAAAAATGTGGTTGACAACCCAACTGCAGTCTGGTGGCAACTTTACATTTTCTAGCTCACAGGATCCATTCTTCGAAATTGGTTCTGATGTCCTCTCTCCTCGTAAAATTGGAAGTGCCTTGGCTGAAACTGTTGAACTTGGAGCACAGTGCTTTGCTAATCTGCAAACGCCTTCCGA

>TDF267-1

TCTCCTTCTCTTTCAGGTTCTTTGTGGGGTCAGTGAAATCGAAAACATGACAACACAATTGAAGCTTTTTTATGAACAAGTTCTGCTTCTCAGAGCTTGGAACATCCCTAAAACTAGGCAACGCCTCAAACGAATTGGTATCGAAGCTGCCATTTCCGTTTACTTCCTCTGCCTGGGA

>TDF268-2

TCATCTGCAGGTTTTACTTCTTCAGTCTTGGGGTTCTCCTCACCTCCGTCTTCAGGCAAGTCAGAGGTCCAAAGAGTGAGATTGTCTCTCAATAGTTGCATAATTAGGGTGCTGTCCTTGTATGACTCTTCACTCAATGTGTCCAATTCTGCAATGGCTTCATCGAACGCTTGCTTGGCCAAATGGCATGCTCTTTCGGGA

>TDF269-1

TCTCGTCGGTTTTCGGGGCGTTAGGGAGTACTTATGAGCATGCTAAGGAGGCTGTCACGGGGAAAACCCATGACGCCGCTGAAAAGACTAAGGAAGCTGCTGATAATGTGTCTGAGTCTATGAAGGATAGTCATGATGCCGCTGCGGTACGTGCTAGGGAGGATGCTGCTCTTGCCAGAGAAGGGAAGGATAATGCTGCTGA

>TDF270-1

TCAAATTCATTTCTGACACTCTGACAAATGAATGCACCAAAGAAGATGGTATGTAAGAATGAAAAATTTGCTTGATCACATATTTTTCAGATCAACTGTTCTTCTGGCATTCTTTTGAAGCTTGAACTCCGCCATTGTGTCTGTTGCTTATGCTGTGTTTATTTTGCTTACTGCCTTATTATTAGTACTATGTGTACCCCTGCGTAAAATATAGCCCCTCAAATGTATTATTGTTTAGGCCGTATGGGGTGTTTTTTTAGGAAACAAAATGCGGA

>TDF270-3-1

TCCAAAATCGAGTCCAATTCCTTCAACAAACCCTCCTGACCTTTCGAAGCAAAGCTCAACCCATAATTCAATACTTCATCACCGTCTTTCTGGTTCGAATCCGAAAACGAGAAGAAATAATGCGAATCATCAACTTTTACAATCGCTTGCTCCTTGATCAACGGCCATTGAACTTCGTCGGAAACGCAGGCGAGTACAGTGACGATGTTATCCCCTTGACGAATACAGAGAACGGAGAAATCGCCGATTGCTAGCTCAACACTGTACTTCTTATCGA

>TDF270-3

TCCCACTTTGTGATTGCAATGCACTCATATTTGCCTGCTGAGGAGCACTAACAGGGTTCTGTTGTAGAGAAGCCATTGCAACTTGCTGCATTGAACTAGGAGTAGTTCCCTGTCCAGAATCTAAATTGGAATTGGTTTGCAAGGAACTTGGCATGCTTTGCTGTGACGAAGAAATAGCAGATATAGAAGGAACAGAACCATGCTGCAAACTTGAAACGTTGCTCTGTTGCATCGTCGAAACAGAACCTTGTAAATTCATAGTCTGTAACTGAGGGTTGGCACCAACCTCATGAGGTGCTTGAGAAATCTGAGCATGAGGCTGCTGTGCTTGCTGCATAGACTGCATTTGAGGTGGGA

>TDF270-4

TCAAAGGAGTCATCCCACTTCCTGTACTCTGTATAAATAAACTCCTTTTTCTGCAAACACTAAGCTCCAACAAACCCACAATATTTACAAATCAAAGTCGAAAATGGTAAACACTTTGCAAATCCTAACATTCATCTTTTTATCAGTCTCAGTACTTATCCTAAACCAAGTCAAACCTGCACAAGCAAAGATCAACAAAACTCAGTTTATTGATGAAGTCCTAGACGGGCATAATGCAGCTAGGAAAGCAGTAGGAGTTCCACCTCTGAGCTGGGAACCACTGCTCTCAACGTATGCACGGATATACTCATCAAGGAAAAGACGAGCAGACTGCAAACTGATCCATTCAGGGGGTGTGTTTGGGGA

>TDF271-1

TCGTCAACGTGTCCCTGGGGGCTCATCGCTGCCAGTTTTCAAGCGAACCGACTATGGATCATCTGGACCACCTAAAGGGGAAGGGGGTAGTTATGGTAGAGGAGTGAACAGGTGGGATACTCGTTCAAGCGGACGAAGTGATAATGACATAGATTCAGAGTTAGGAAGGCGCTATGGAGGCCAGGCTAGGAGATCCTGGCAGAGTCCCCCTGAACATGATGGTCTTCTTGGAAGTGGTTCATTTCCTAGACCTGCAGGATATGCAGCAGGTGCAACGGTTCCAAGATTTCGTCCTAATGA

>TDF272-3

TCCCAGTGGGGGCTTACGGCTGAAGCGTGACATAACAGAGTATGGAGAATTTGTTCGTAGCTTCAATGCTCCTTCCGTTGATGAGCATTTTGAAGTGTTGGGAATTTTGGCCAATGTCTTCATTGTTGCTCCTGAAAGTCTCTCTACTCTCTTTGAAGGGACTCCAAGTATCCGCAAAGATGCACAAAGATTTATTCAGCTGCGAGACGACTACAAGTCTGCAAAACTTGCAGCAAAACTCAGTTCATTGTGGCCAAGTCTTGGTTGATATTGGGATTGTCATTTCAAGTGGAAGTTTCAAACCACAAAATCTGAAGAAGCTGCCGTGATAATTTTCCGAG

>TDF273-2(1)

TCACACACTGAAAAGAAAGGAGATAGATGATTGAGGTTAGTCAAACTTAGGTAAAGGTTTTGCAGTTGATCGACTTTGGTTTTGTACTATTGA

>TDF274-1

TCCAACTTCAAATCACGATGGAAAACACCCACGCTATGACAATAATCAACAGCTGAAATCAACTGTTTGAAATAATTCCTAGCAACATTTTCATCCAATTTCCCTTCTCTCGAAACCTTAGCGTGGAGTTTAGGA

>TDF274-2

TCTTGGAAAGTTTGTTGGGAGCACCACCAGTGACTTTAGCGACACGAAGGAGAGCGAGCTCATTCTTGAGTTCTTTCAGCTGAGCTGTTAGATCTGCTCTTGTTTTTTGCCTCAATTCATGAACTTTGATTCTTGCCATTTCGATCTGATGGTGGA

>TDF274-3

TCCTTCCCCAAAGTCTATGTAGATATGGCATCAGGGCTAGCATCTCCATAAGCAACCAGACCTTTCTGCCCGACAGGTGAGCTTGAGAGTGACTTGGTAGGAGATGCAGATACTCTCTTCCGGTGCTTCTGTGAACCAGCATCAGGTGAGCTTGAGCGAGAAGAACTACGCCGTCCCCTGGACCTTGATCTTGACCTTGCTTTTACTGGAGAACGCCGATCATCTGCTTTGGGGCCAAGACGAGATCGCAACCTATCACTAATTATTGGTCGTTTCTCCTTCGGACTGGACCCACGAACTGGACTTCGGCTTCGATCCCGTCTGGTCCCACGATTATCACCTGGACTGTGGGAGCGACTTCGACTCCTCCTTGGGGATCTTCTTCTTGGAGGGCTCCTATAGTGACGACGGGGTGACCGATCTGAATAACCTCTATAGCTTGAAAACCTATCACGATTCCTGTCATTATAATTCCTAGCACCATAGTTGAATGGCCTACGAGGTGAGCGCTCTGGAGATGGAGTCCTGTATCGGCGGGCAAAGGA

>TDF275-2

TCCAGAAAATCAGGTGCAGAAGAAAGTTCAGAGAGTGTGACTGCGTACCAATTCTCCCTCTGCTTATCCCCAACCAACGGAATGATCTCTCTAGGTGATCATATTTACGATAATGCCATCTACAAATCCATGACTTACACTCCCCTCATCGAAACCCAAGACTCCTCCTCCTCCTACACAATCCCCATAAAATCAATAAAAATCCAAAACAAAAGGCTGTCACCAACCATCGAACCCTCGTCGA

>TDF275-3

TCAAGATATTGTAAATGGTGGGCAAAGCTCAACCGTCCATGATTCCCTCCCCACGGTAACAGTGGAAACCCTAGAGAAAGGATTTCTAATAAATGTGTACTCAGAAAAGAATTGCCCTGGTCTTTTGGTTTCTGTACTTGAAGCTTTCGAAGAACTGAATCTTACTGTTGTTGAAGCTAGGGCTTCTTGTACAGA

>TDF278-1

TCATGTTGATGGTCCTACTTTTGTTCCTGCTTCATCTTTACCGTGGTGGTCAACTTCATCTTACACTATAAATCAGCTGTCCTTTTCTGTTCCACCGCCTCCAGTTACCCTTTCTGTGGTGAGTAGAATTAGAACTGGTAACTCAGGTTGGCTCAACACAGTCAGCAGTGCTGCTTCTTCTGCAGCCGGAATGAGCTCTGTGCAACCCTCTGCTGTTTCTGCTGTTTTCCACTGTTCTAGGCCTAATGGTTTGCCACCTAAACCCTCAAATTCATTGGAGCACCTTTTGGTTTACTCTCCTTGCGGGCATCTTATCCAATACGGA

>TDF278-2

TCCAACAAGTTACAAGGAGTTGCAAGTAGGATGGATGACTGCTTATCTTTTCATCGTCTGTCTCGTTGGACTCTTTGTATTGGTCCCTCTAAGGAAGGAAACAAGTGCGTGGATTTGTGAAGTACTTTTCATTGAGCTTCATTTGGGGGTTTTTCCAATGGTTTTTCACCGGTAAAGAGAACTGCGGATTCATGCAGTTCCCTACCTTTGGACTGGAAGCTTGGAGGCAAACGTTTTACTTTGATCTCAGCATGACTTATGTGGGAACAGGAATGATTTGTTCTCATCTTGTGAGTCTATCTTTGGTCTTTGGAGCCGTGCTGTCATGGGGA

>TDF279-1

TCCAGAGACAGCATTCGCCTCGTCAAACGTTGCCACAAGCCTGATCGCAAAGAATTCGATCACAAACAAAGTTGACGATGAAGCAAAGTACTCTTCTTTCCCTGCATCATACCATTTCGGAACGTTGAGGATTCCGAACTTGGTGAACACCTCTGGTAGCAGCATTCCTGCTACTCCGAGCATTGCCCATCGCCCATTCACAAGCTCGGCCTTTCTTCCCGATTATCATCTGTATTCTCTGAGAAAAATGGCCCCAAGAACTTGTGGATGTGGAGACAACTGCAGCTGTGGCGACAGCTGCAACTGCAACAGGATGAACAGGGTTGAGTGTGGCTGTGGAGATGACTGCAGCTGCGACCCATGCACCTGCGA

>TDF279-2-1

TCCAACAAAAATGGACCAAAATCAGAGACCATCATCCTCTTCCAGATCTTTTCAATTACACCCTGCTAAATCTCTTATCACTGATCTCTTCAACCTTTATTTAGGGAGAAGTTCGCGCCAAAAACCCGAAGAACACCCGCCTAACAAGACACAAAAACGTGTAAGTGGACTCAATAAAGATCTTCCTCCTCCAAATGAGCAATTTGTTTCAGACTTTGAAC

>TDF279-2-2

TCATGTTTCCAGGAAGTGGTCTGAACAAGCGGATAATGGTGCTAATATGCTTGTTACCGTCCCCGGTGGAGGGGATGGGCCGAGTGGTGTGTTAGTTTGTGCCGA

>TDF304-1

GGGTCAACCTGTATCCAGTTACCATGTTAAAGCCACCCCTTTGAAGGGGACTAAGGTGGTTGTGGAACCGAAGGTGCTGAATTTTACAAAGCTGTATCAG

>TDF304-2

AGCTGAAGTTGAGGAGAGGAATTTACTGGGTGAAACTTTTACTGAGTTGCT

>TDF305-2

AGGAAAACTACTCATTAGTGCATCGGCAACTGTTGGAGCCTTGCTTTTGGTGGCT

>TDF309-3

AGCATGGTGAGATGTCATAAAGGGAGTACATGGCTTGCATTTGGTCCTCGGAGTATAACTCCATCACAGGTGGAGTGATCTCATTCGGGCATGCGCGAGAACGTAGCGCGTTCCCCATGTAATGCCCGAGTCTCTGTTCT

>TDF310-2

AGCAAATATGTCATCTGGGTTCCGAGAACTGAACCGGAACGAAGTGGGCCCATCTCCAGTTGAGAAGAAGGTGGCACCCCCGGGCCCAGCACCAGAAGCATCTGGTGGTGGTACCCCACCT

>TDF311-3

TCAGCACAAGCACTTCCTTATGGTTACCAATCAACTCAACAAGTGTCATTGCCTTCGCCAAATGGTGCCCCGAATAGGAATGAGCATCACTTCTCAGGAAACCCGATGAATGGCACCGGAATCCGGAGAAATCCAGGCATCCCTTTGCCT

>TDF312-1

AGGGTTGCTGACAGTTTGGAAATTATTCATTGACAGGTAGCTGGGGACTATGTGAAACTGAACTAACGATACTTTCTGTTGGTCTGAAAGTGAGTTTAGTAGCCCTGACTTTAGGCTTGAGAAGGCGTTATCAGGTGGTGCAAACATTGTCAGCCCT

>TDF313-2

AGGGCATGTATGAGCAAGTGACCAACCCATGTGGCGGTTTAGAGATGTATACTAATAAGGCAATCAGATGGATGTAAACATTTGAAAATTTTGCTACTCCGTTTTGCAAGAATCATGATGAGGAGACAGATTTTACAGTGAGTCATTTCTCACAGACTTGCAGTCGAAGTTCAGTGACT

>TDF314-1

AGCTCCTTCGACTACAAATTGGTTGCCTGAAGATTATGTTGAGAGGGTAAAAACTGTTCACGAATCTGGTGGTTATGAGTCAAGAGGGTATGGATATGACTGGAAAAGAGAGGAAGCAAACAAAAACCTTCTGCGAACACATACAACTGCTGTTTCTTCAAGAATGCTATATGCACTAGCACAACAGAAATCATTCACTCCCAAAAAATACTTTTCTATAGATCGTGTGTTCAGAAACGAAGCAGTTGACAGGACTCATTTGGCT

>TDF314-2

AGCGCAAGCAAAAGGAAATTGAGGAACGTGAAAGGGAAGAAGCAAAAGCTTTACTTCAGGGGAAGTTGGGTAAAAAACAAAAGAAACCCTTATTAGAAGGGGATAAATTGACTAAGGAAGCAGTGATGAAATTAGCTCTCAATGAGCAGCTAAGGGAAAAACAAGAAATGGAGAAAAAATTGCAGAAGGTTGGCAAGCAGATGGACCATCTGGAGCGAGCTAAAAGAGAGGAGGCTGCTCCT

>TDF315-1

AGGAAGATAAGTTTCCCAAACAAGCCTGCTGGCTTGTGGATTGGCAATTTTCATTTTTCCGGTTACAGGATCTCGCTGCT

>TDF315-3

AGGCTCTTAGCTCGTTCGGCTTTCAGTATTTGTCTCAGGTTTATCTGCCTCTAAAGTTGCAGCAGCGAGATTGGCT

>TDF316-1

AGGGCTAAGCAAATTGACTGATAAGTTTCTCATCAACAACAATACTGTTTCAATATTTATCGGGTCCATTGCCT

>TDF318-2

AGCGTCTTGGACTGAGTGAAGGAAATGATGGAATAGATTGCAGGATCAACTTACGGCTGCTCCTAAGACTAATAACAGACTGTCCCTTTTCACGTTGTGGAGGTGAGACCCTCTTACTGTTTGGAGACAATGCCT

>TDF319-3

AGGCAACTTGTCACCCTTCTTTTATGGAGCAATTATCATCTTCAGCAACTGCTGTCGAATCAAGGGTTCAACAGGACGGTAAAGTTGTTACTAGTCGAGGACCAGGTACAACTTTGGAATATTCTGTTACTCTAGTAGAACAGCTACATGGCAAGCAAAAAGCTGATGAAGTTTCTGACCCTCTGATAATGCGGCCCAAGCATGGGGATGAGTTTCTATTCAGGGAGCT

>TDF320-1

AGTGTCATATGGAACTTTGAGCAAGTGAAGAACAGTGGTCATGAGTGCTACTGGAATCAACAGGGCTTGAAGAGCTGCTACTGCTACAAGATTGTGTTGAAAGACGAGTCTATAGTGGACAGTCGATTCATGCACGAGAAGTTTGCAGCTAGTGACTCGCCAGAAGCTCCCCTTGTGGTGGCAACTCCTATGAAAGTGACTTCATTCAGTATATCGACCAAACAATAGTAGTAAATCACTGTAGTTAGTCTTCTGTCACAGAAATCTCACGATTGTGTACTGCTTCATTTGTAATGTTTCAATTTGTTTTTCTTGTAGCGGTTCATCTATGCTTGTAGCTAGGGCT

>TDF320-2

AGCCCCACATAAGAAGAACAAGCAAACTTCCACATCCAAATTGAAATGCTGTGATGGTTGAAGGGAATGGATAGACCCTAAGAACCTGCTTGTTGTAAATATTGAAGAAAATGTTCAACAGATACCAAAGGCAAAGCATGCTACCAAGCTGCAAAGGATGAGCTAGACTAATA

>TDF320-3

AGCAAATCAGGATGAACCTGATGAGGCTAGGGCTATGAGAGTGGGTGTACCCTCAGAAGAGGGAATTGTGGAGAATGATGTGAGAGCTGAAAACTACAACACATCTGCTGTGGAGTTCTTGAGACGTCTACCAGGTGTCTCGGATTCAAATTACAGGGCTATAATGGATGCGTGCAACAACTTGGCTGACCTGGCTCTTCTTCCAGTTGAGAAGCTGGCTGATCTGATGGGTGGACAAAAAGCTGCTCGCACTCTTAGG

>TDF322-1

AGCCAAGACAAGATGATTTGCCGCCAATGCTATGCTCGCCTGCACCCTCGTGCTG

>TDF322-2

CACTCTTCTACCCAGATTCAAAAGGGTAGCTTATTTGGATACTGATGTTGGGCAGCCT

>TDF322-3

CACCTAATGCGGGAATGCGTCTCTACGGTGGTGCACAGTATCATCGTGCTATGGCT

>TDF323-1

CACCCCTGTCTAAATGAATTGCTAAGGTAGGTATTCGCATTATAGGATCCT

>TDF323-2

AGGCAACCTTAGCGGCACCAGTAAAAGGGAAGATGTTGAAAGGGCTAAGTCTGTGTTATGCAGTTGTGCTG

>TDF325-1

CATAGCAGATCAATAATAGTTGAATATCGCTCTGGAAGATGTCAAAACTTGGAGAGTAGGGGAACAAAGTGTATGCT

>TDF325-2

AGCACAAGGTACTCTGTATATTATATTGTAACAATATATCGCTCACACCTTCCTCCATTTGCAAAACAACTACGTAAGACAACAAAAAGAAACGGAAATGCATTAGAAAACATTCTG

>TDF327-1

CATCTCAAGTAGAAAATCATTCCATGTATCAATGGGCCCAGGCAAACACCAATGGACACAATCATTGTATAAAACCACTTTTTCATTGGGCCCATGTCCATACCTACTTGGGTGTCCATCGGGCCTCATCAATATGGTTTGGGTCAAATCAATGAGCCT

>TDF328-1

AGGCCATCACAAATGCATAGTGATTCATTGCTCTTCTTGGATTGGCAAAAGAAGCCGAGAACAGAAGACTCCAAAGAGGCCGCTTTTTCACTAGAATAAGGACCTCCCCACTCTTGAGTCACTGCCTCTTGGAGCATCCACCAATTTGAGAACACAAGTGATAACCCTTCTTGAAATTGCACAAGGGCTTCTCCTCTTAGCTTCGAACTCTTAGGGCCCTTCATTGTCTGTGGCGTGTTG

>TDF329-1

AGCTGCAATGCTTTCATGTACAAGCAATCTAGATGTCGCACTACAGATTTGACCATTTGTCCAAAAACAACCAAAAGCAGCCCACTCAGCAGCTTGATCCAATTCAACATCTTCAAACACGATTATAGGACTTTTCCCTCCAAGTTCTAATGTAACAGGCTTGACCATTTGAGCAGCAGAAGACATAATCTTGCTACCAGTTGCAGTACTCCCCGTAAATGCAACCTTGTCAACATCGGGATGACTTGCTAATGGGGCACCAGCCTCTGGACCTAATCCTG

>TDF329-3

CACCTATACTTTCACCCGAATGTGTTTCTAATGTATCCACACTCTGCAACCTCTGTTGTGCCACCCAGGAGTGTTGAACCATTGACTGCTTCTGACCAGACTGAAAAGTATCTAGGCTTTGCTGTACAGCTTTTTTCAAGTCAGCTTGAAACCTTTCCTCTTCATCCTCCTCAGCTTGTAGTTGTCTAAGTGTTTTTGACCCAACATCTCCAACACTACAACTGCGACCACTATCCTGATTCCAGACCTGATCGGTCTGCAGCATATCAGTAGCT

>TDF330-1

CACCGGCACTGTATCCCAGATTAGCCAACCAGCCTTCCGTACCTTTGGTGGAACAGAGCCACATCCAACAGAAACTAAGCAATCAATTTTAGTGTCAGGCCACAAAAGTTGAGCTTCCCGTATTGCAAATATGGTAGGGTTATTTGCTACTATAGCACCATCTTGCCAGCGATTGACATCATCAGAAAAATCATCTAGATAATATGGCGCAGCAGATGAAGCTCTTATAGCTTGCCAGACCATGTGTTTACAGCTCCCAATAAAAGCATTATGTTTGTAACCGACTTCCCCACTTGTATTGTCTCCATCTGACATGCTGATTGCT

>TDF332-2

CATCAAACTGGGTACTCACAGGAGACTCATTCCAGCGCATAGCAAGGGTAGACCTCCATCATACTATATTGTTGCAGGATTTGTTTTTACAACTGTCTCGGTTCCATATCTTCGTTCTGAGTATGGAAAAGAGTACGAGTATGAGGCTCCAGTGAAGCTTCTGGACAAGCTTTTGCATTCAATGCCTCAATCAAAAGATGAGGAACTTGTTGTTGTATCTCAGGTTCTTGTAGCTGACATCAATATTGGGTATGAAGAAATTGTGAACACACAGGTTCTTGGCTTCAATGGTAATCCAGTGCGGAACCTGAAGAGTTTGGCAAATTTGGTGGAGTCCTGCAACGCT

>TDF332-3

CAACAATAAGCCTTTCATGTGCAAGTGCATGGGTCGCATTTGCAGTTTGGTCCGCACTTGCATCCACCGTTCTCAGCAGCTACTTCCATCTCTGATCCCTCGTA

>TDF333-4

AGGAGTAGTCCTCATGCCATGCACCAAGCAAGTAGAAGACGCGAGCTCAGCAAAGAGTGACATTTTTGATTCCGAGAGCCCACCTTGCACGGATAATGCAAACCAATCATCATCTGCGCTTGTAGCAGAGGCTTCATCCACCCTTATGCGCGAATTGGACCAGCAGTCGGATTTTTCTCAAGAGGACGAAGAACATTTGCTGATCAATAACAACCTACTACTGCCTCAAACACCACCAGCATTCAACAATTTTCTAAAGCTTACAGACGATG

>TDF335-2

CATCACAGGCTGACCACGGATAAGTTGCCCAGAGAGTGCAATAACCATTGGAACTGACATCACATCATAAAACTCAATATACCCAACTCCTTTCGAGCGCCTAGAATTACGATCCATGATGAGGCGGACATCTCGAACCTTGCCGACTCTTGAGAAAAATTCAAAAATATCCCGCTCATCTGCCCTTAGAGGAAGCTGGTATGCAAAAACCGTCCTTTGATCTCGTTCAGGATCAGCCTCTGGTTCAGTTGTTTCATCCTTCTTTTCCTTGTGACGTCTGCTTTCTTTGTCACGTACATCTCTTTCATGAATCTTATCTCCATAATCTCTGTCTCGGCTTTTTTCCCT

>TDF336-1

AGGATCGCGTTCAAGAGACTCTGAATAACTCTCCAAAATCGCAACAATGTCATCAAACTGTGGTCGTTTATCTGGATTGCTTGACCAGCATTGCTTGATCAGGTGACTCAATGCTGCAGGGCAATCATTGGGCATTGGAGGCCTTGCATTCTTCTGGCAAACAGCAAAAGCAGCTTGTTCAGGAGTCATGTTGTCAAAGGGTG

>TDF337-2

AGAAGCACGATGAAGCTGCTGTCCAACAATTCTGTGCTGAGGTTCAAGTGAAGCGTCAAGTTC

>TDF338-3

GATGCCTAACATTTCTGGCAGCTTCAAGTCGACTGGTGTGCATGTTGGGTGACT

>TDF339-1

AGGAGCAGATGGTTTTCCTCTCTCGTGATCCAACTATCAAACTTCCTGTTTCATTTGACAAGGGAATACTACATGCCAAAAGTGGAGGACGTTATTCTAACCCCAAGACCGTCAGACAAGTTCTTGAGACTC

>TDF340-2

GAGGTTGGATTTTCCTGAGATGAAATTTAGCTTGTATTTCATGGGTTATGAGGATTCTACTTCAGCCCCAACTGATCCAGTAGATCGAACAGTTTGGACCTTCAGTAAAAAAGCTACTATTGAGCTTACTCATAATTGGGGTACAGAGAGTGATCCT

>TDF341-1

GACCAAGCTTGCGGTCTGTCGGAACAAGTGGTGTGCCCCACGTTCCCTTCGCATCAGCAAACACTAGCTCGGTGACTTTCAAAAGTTCTTTGTCAAAGGGTTTCTTCAGAATTGGAGCTTGGGATGACCATGATTGCTCCAGGGCTGTCGAGAAAATCAATGGAAAATCATAACAGTAAGTTGTGCTGCTTTTCGTGGCCT

>TDF341-2

AGACACCTTCAACCTAAGACCTGGGCTTTTCCTTCTTTTCCTTGAAGAGGGCAAGAAGCGAGACACCTGCAACCTTGACGACCTTGAATCGAACTCCGGGAATATCTCCCACGGCATGTCCTTTACGACCAAATCCAGCAATCAACACCTCATCATTCTCCTCGATGAAGTTCAAACAACCATCATTGGGCACAAAAGCAGCAATCTTCTTTCCATTCT

>TDF342-1

GATCACTGGAAAAATACTACGCAAAGTGCTGCTTCGGCAGCTAACTGTTTCATTCAGTGTGCAGAGGCTGCTTTGCCGAAACAGAAACGAGGTGTGGCAGTCACT

>TDF345-1

AGGCAGGCGTACACTCCAGATACTACTGAGAAATGTCCCGAAGCAAAGGCTGCTAGGATGAGATCAGCTACGATGAAGGGTTCATCTACGTCAAAACACCGATCTTCAAAACGACCAGAATATTCAGAATTTTATGGCTCGAGTGAAGCTCCTCATCATAGTGGACAAACACGGTCCAAAGTACACAAAGAGAGAAAGCACCGCCACCGAGATAAGAGCGGAGAAGTTAGTTCAGGGCCCTCTTTATCTGATC

>TDF345-2

GAGAACAAAATTGTGGCTTATGCAAGCCAAGGAATGGATCGAGATTCTTTTCATGTAGCCAACCTCTTCTCGCTGGTCTTACTTCGGAAATGGGACAACAAAGAGAAGGGTCGTGAAGGCTTCTTTGGTGTAGTGCCATTACTTGAGCTTTCTTTGTGAGTCAGAAGGTTTTTCACAGTAAACACTTCAGTAGATGAACCATTCGATGCATCATCAACAGTTGTGGTGGCATCAGCAGCTGCTTTATCTGAAACAATATTACGTTCAGATGCTGCT

>TDF347-1

GAACAAAGCGATTAGAGACGCTTCAAAGGGCAAGACAAGCAGCCTTTTCTCAATACGAGATTTCAATGAAGCTCTCGTA

>TDF347-2

AGGCTTTGAACCTGATGTTGGTTTGTAGTAGAATTTGGTCATGTTTTTTTTATCTTTTCATTTTTATTAACATTCAAGCGTAGAAGTTATACTCACTGCTAATCGTTGTACGTACAATCCTAAACAATGCTCTGAGATGATATTAGGAACATGATTTATTCGTGACCAAGTACAACATAATACTAATACAGTTTCATCAAATCAACCCATTGTGTAGGTTGGTGTAGGCAAGTTGATTACTGTGCT
