# Supplementary figures and images for: The Developmental Delay of Seedlings With Cotyledons Only Confers Stress Tolerance to Suaeda aralocaspica (Chenopodiaceae) by Unique Performance on Morphology, Physiology, and Gene Expression
Source: Front Plant Sci. 2022 Jun 6;13:844430. doi: 10.3389/fpls.2022.844430 (PMC9208309; doi:10.3389/fpls.2022.844430)

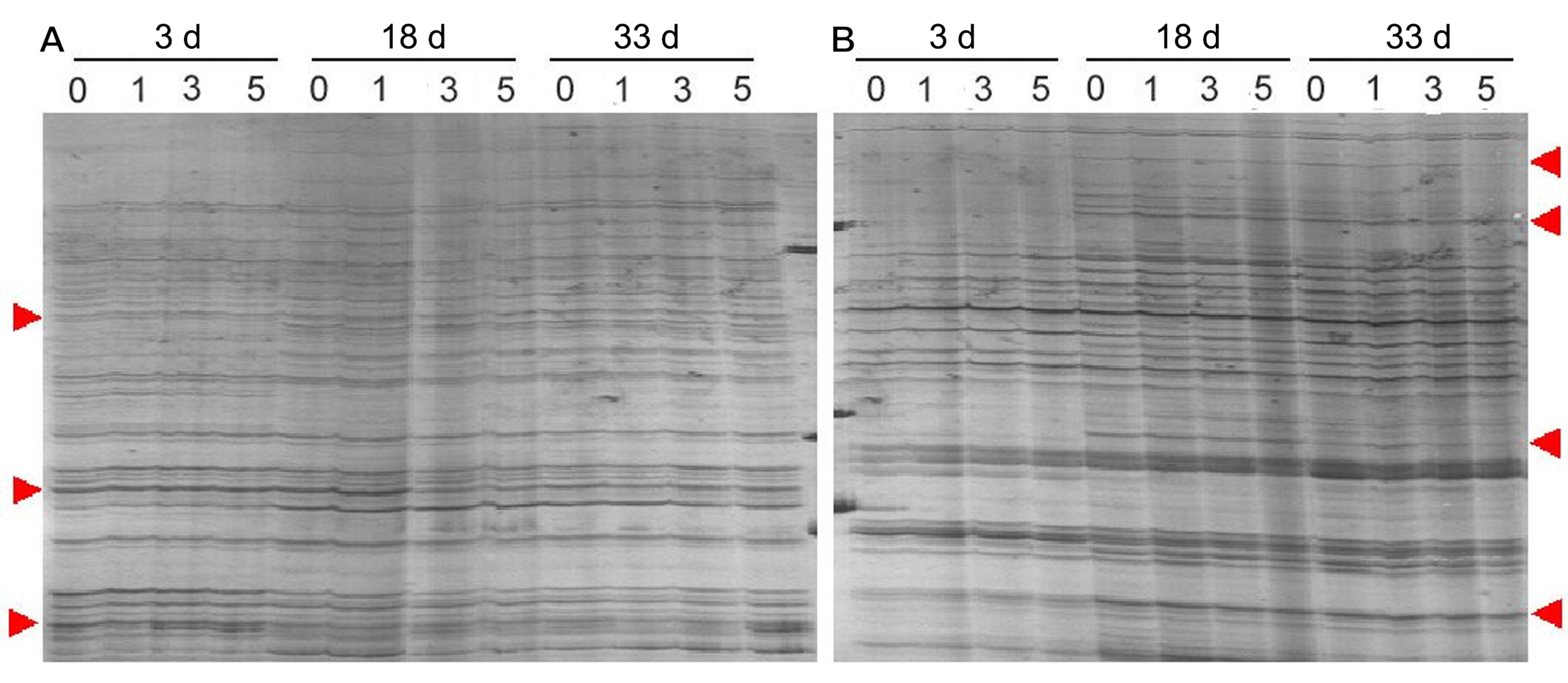

Supplement: Supplementary Figure S1 — A representative result of the silver-stained cDNA-AFLP gel showing differentially expressed genes at different developmental stages in seedlings of S. aralocaspica under salt stress. (A) Seedlings from brown seeds, (B) seedlings from black seeds. 3 d, 18 d, and 33 d represent 3, 18, and 33 days after seedling emergence, respectively. 0, 1, 3, 5 represent 0, 100, 300, 500 mM NaCl treatments. The red arrowheads indicate TDFs with a differentially expressed pattern. Each group was amplified with a different pair of selective AFLP primers. [file Image_1.JPEG]
