# Supplementary material for: The Developmental Delay of Seedlings With Cotyledons Only Confers Stress Tolerance to Suaeda aralocaspica (Chenopodiaceae) by Unique Performance on Morphology, Physiology, and Gene Expression
Source: Front Plant Sci. 2022 Jun 6;13:844430. doi: 10.3389/fpls.2022.844430 (PMC9208309; doi:10.3389/fpls.2022.844430)
Supplement: Supplementary Table S1 — Relevant parameters of four phytohormones. [file Data_Sheet_2.docx]

**Table S1 Relevant parameters of four phytohormones**

| Phytohormone | Regression equation | Relation  coefficient (R) | RSD of retention time (%, n=5) | RSD of peak area (%, n=5) |
| --- | --- | --- | --- | --- |
| ZT | y=(4.6001×10^7^) x -4.631×10^4^ | 0.9957 | 0.00089409 | 0.04369694 |
| GA3 | y=(4.5507×10^5^) x +3.453×10^4^ | 0.9944 | 0.00091806 | 0.06152035 |
| IAA | y=(1.8648×10^7^) x -4.318×10^5^ | 0.9975 | 0.00119096 | 0.11835314 |
| ABA | y=(2.9953×10^7^) x -1.745×10^4^ | 0.9989 | 0.0014325 | 0.0630877 |

Note: *RSD*, relative standard deviation

**Table S2** **Sequences of the adaptors and primers used in cDNA-AFLP analysis**

| Adaptor or primer | Sequence | |
| --- | --- | --- |
| Adaptor | *Eco*-ad1: 5’-CTCGTAGACTGCGTACC-3 | *Mse*-ad1: 5’-GACGATGAGTCCTGAG-3’ |
|  | *Eco*-ad2: 5’-AATTGGTACGCAGTCTAC-3’ | *Mse*-ad2: 5’-TACTCAGGACTCAT-3’ |
| Nonselective PCR primer | *Eco*P: 5’-GACTGCGTACCAATTC-3’ | *Mse*P: 5’-GATGAGTCCTGAGTAA-3’ |
| Selective PCR primer | *Eco*1: 5’-GACTGCGTACCAATTCAC-3’ | *Mse*1: 5’-GATGAGTCCTGAGTAAAG-3’ |
|  | *Eco*2: 5’-GACTGCGTACCAATTCCT-3’ | *Mse*2: 5’-GATGAGTCCTGAGTAACA-3’ |
|  | *Eco*3: 5’-GACTGCGTACCAATTCGC-3’ | *Mse*3: 5’-GATGAGTCCTGAGTAAGA-3’ |
|  | *Eco*4: 5’-GACTGCGTACCAATTCTC-3’ | *Mse*4: 5’-GATGAGTCCTGAGTAATC-3’ |
|  | *Eco*5: 5’-GACTGCGTACCAATTCAG-3’ | *Mse*5: 5’-GATGAGTCCTGAGTAATA-3’ |

**Table S3 qRT-PCR primer sequences used in the present study**

| TDF code | | Gene abbr. | Annotation | Forward primer (5’-3’) | Reverse primer (5’-3’) |
| --- | --- | --- | --- | --- | --- |
| **Seedling development related** | | | | | |
| 158-1 | *ABCB20* | | ABC transporter B family member 20 | GGAAGAGTAGCTAAAGCCACCAG | GCTATGCTCCGAAATGAAGTTG |
| 4-2 | *APC11* | | Anaphase-promoting complex subunit 11 | CCCTATATGCCGTCGAGAATG | GAGTAAAGCCAGCGAGTTGC |
| 128-1 | *ARF3* | | Auxin response factor 3 | CATGTTTTGCAAGACTCTTACTG | CCAATTCCTAGCCTCAACTCTC |
| 132-2 | *bHLH147* | | Transcription factor bHLH147 | TCATGTTCGAGCGAGGAGAGG | CGGGGACAAGATCTTGGAGTATTC |
| 13-4-2 | *BLH1* | | BEL1-like homeodomain protein 1 | GATAAGGATAAACAAGGCGGAAG | GAGTCCTGAGTAAGAGCCAACG |
| 192-3 | *CLPA* | | Clp protease ATP-binding subunit ClpA homolog CD4A, chloroplastic | CCCAGCAAGCATCTTCTCAG | CAGGCCTGAGTTCTTGAACAG |
| 314-2 | *eIF3a* | | Eukaryotic translation initiation factor 3 subunit A | CAATTCAGCGCAAGCAAAAG | GCAGCCTCCTCTCTTTTAGCTC |
| 197-1 | *Exo70a1* | | Exocyst complex component EXO70A1 | CTAGTTGAGCAGGAAATGAATGG | CACGATCCGAATTATCCCTAGG |
| 119-3 | *LOX1.5* | | Probable linoleate 9S-lipoxygenase 5 | GTACTAGACGCTTTTGAAGGAGG | CAGTCCTCCAAGCTAATTTATCC |
| 13-6-1 | *LST8* | | Protein LST8 homolog | GCAACTGCATCATCTGATCG | GTCCTGAGTAAGAGCACAGCAAAC |
| 196-2 | *SYNC1* | | Asparagine--tRNA ligase, cytoplasmic 1 | TGTGACTTGAAACATCTCGCC | CGTACCAATTCGCAATGAACTTTC |
| 42-4-2 | *UBQ10* | | Polyubiquitin 10 | GTACCCTTGCTGACTATAACATCC | CAATGGTGTC CGAGCTCTC |
| 320-3 | *UVH1* | | DNA repair endonuclease UVH1 | GAGGGAATTGTGGAGAATGATG | CGTACCAATTCCCTAAGAGTGC |
| 236-1 | *XDH1* | | Xanthine dehydrogenase 1 | CGTAGCAGGATTGTCAAGAGG | CTTCGAAAGCTGTTGGTGAG |
| 278-2 | *YSL3* | | Metal-nicotianamine transporter YSL3 | GGAAACAAGTGCGTGGATTTG | GCTTCCAGTCCAAAGGTAGGG |
| **Salt response** **related** | | | | | |
| 278-1 | *ATG18H* | | Autophagy-related protein 18h | GTTCCTGCTTCATCTTTACCGTG | GGCCTAGAACAGTGGAAAACAG |
| 217-2 | *AVT6C* | | Amino acid transporter AVT6C | CTGCAGCAGGAGTTTAGGTTC | CCTGAGTAAGTGGTTATGATTGCG |
| 329-1 | *BADH4* | | Betaine aldehyde dehydrogenase, chloroplastic | CGTACCAATTCAGCTGCAATG | GTCTTCTGCTGCTCAAATGGTC |
| 129-3 | *CBSX3* | | CBS domain-containing protein CBSX3, mitochondrial | GTTGGAGCATTGTTGGTTGTG | CACGAACAACATCTCCAATTGAC |
| 319-3 | *DJ1B* | | Protein DJ-1 homolog B | GCAACTTGTCACCCTTCTTTTATG | GAAACTCATCCCCATGCTTG |
| 268-2 | *GRF12* | | 14-3-3-like protein GF14 iota | GGTTTTACTTCTTCAGTCTTGGGG | CGAAAGAGCATGCCATTTGG |
| 114-1 | *MDAR* | | Monodehydroascorbate reductase | CTTGGAGGTGGAGTAGCAGC | CATGTATGAAATGATGGAAGCC |
| 56-1 | *MKK4* | | Mitogen-activated protein kinase kinase 4 | CGGGAGTTTAGGGACTTTATTCG | GGAGGCAACATTTGACGAAG |
| 104-1 | *OLP1* | | Osmotin-like protein | CCTATAGTGGGCTGTAAGAGTGGG | GATGACTTTGAGCTCGCGTG |
| 241-1 | *PBL7* | | Probable serine/threonine-protein kinase PBL7 (CDL1） | GCGTATCAAGCACTAGCAGTTG | GTTGTTACAGCTTTACTCAGGACTCATC |
| 34-5-1 | *PGK3* | | Phosphoglycerate kinase 3, cytosolic | TCATCAAGGGCAAGAACTCC | CACAATCATTGGTGGAGGTG |
|  | *P5CS* | | Pyrroline-5-carboxylate synthase | GATGCAGTAAGTACCAGGAAAGCTC | ATGAGACCATTCTGCCCAACAGC |
| 14-14 | *SKD1* | | Protein SUPPRESSOR OF K(+) TRANSPORT GROWTH DEFECT | GAGTATCTCCGAAGAGCCGAG | GCATCCTCACCATCCTTCTC |
|  | *V-ATPase* | | Vacuolar ATP synthase | ATCTGGCCAATGACCCTACAATTGAG | CGTTCATAGATGGTAGCCAAATCTGTG |
|  | *V-PPase* | | Vacuolar pyrophosphatase | AGCTGTTATCGCTGACAATGTTGGTG | GTCGGTTGCAAACAAGGTAGTGATC |
| **Photosynthesis related** | | | | | |
| 166 | *ACLA1* | | ATP-citrate synthase alpha chain protein 1 | GAGTCCTGAGTAAAGCTGCACG | CACAGCAGCAACACATCCAG |
| 1-2 | *CHLH* | | Magnesium-chelatase subunit ChlH, chloroplastic | GCGTACCAATTCACCATAGGC | GCTGTGATTATGGAGTTGGAAGC |
| 265-1 | *LUT2* | | Lycopene epsilon cyclase, chloroplastic | GTAATCCAAGTTTAGCAGACTCAGC | CGTACCAATTCTCCCTCTACTATTTC |
| 194-3 | *MDH* | | Malate dehydrogenase, cytoplasmic | GACCACAACAGGGCTCTTG | CAACAAGCTCACGGACAGAC |
|  | *PEPC* | | Phosphoenolpyruvate carboxylase | GACCCAGGAATCGCTGCTTTATATGAC | CACTGTTACATGGAAGTCAGGATCACG |
| 22-3 | *PPDK* | | Pyruvate phosphate dikinase, chloroplastic | CTGTCCCAGGAGTCAAACAC | CACTGAACTAACTGCTTCCGA |
|  | *RUBPC* | | Rubisco carboxylase | ACGGTCGAGCAGTTTATGAATGTC | GTCTTCACATGTACCCGCAGTAGC |
| **Reference gene** | | | | | |
|  | *β-ACTIN* | | Internal reference | CCAAAGGCCAACAGAGAGAAGAT | TGAGACACACCATCACCAGAAT |

**Table S4 The 328 differential expression TDFs identified by cDNA-AFLP against known gene sequences using BLAST**

| **Function** | **TDF code** | **Size (bp)** | **E-Value** | **Gene model name or species** | **Annotation** |
| --- | --- | --- | --- | --- | --- |
| Metabolism | 1-2 | 198 | 2.6E-101 | GOSA_00018375-RA | Magnesium-chelatase subunit ChlH, chloroplastic |
|  | 3-1 | 138 | 3.9E-68 | GOSA_00020449-RA | Glycerate dehydrogenase HPR, peroxisomal |
|  | 3-2 | 130 | 1.0E-63 | GOSA_00022716-RA | Beta-amylase 1, chloroplastic |
|  | 12-1-1 | 158 | 1.6E-77 | GOSA_00014306-RA | Non-lysosomal glucosylceramidase |
|  | 12-2-5 | 67 | 5.9E-28 | GOSA_00008423-RA | Mannosylglycoprotein endo-beta-mannosidase |
|  | 12-4-1 | 72 | 3.0E-31 | GOSA_00012275-RA | Enolase |
|  | 14-4 | 87 | 2.3E-38 | GOSA_00005951-RA | Proactivator polypeptide-like 1 |
|  | 14-33 | 135 | 8.0E-29 | *Beta vulgaris* | Aspartyl protease family protein 2 (LOC104882986) |
|  | 22-3 | 103 | 7.9E-49 | GOSA_00019355-RA | Pyruvate, phosphate dikinase, chloroplastic |
|  | 31-10 | 203 | 2.1E-102 | GOSA_00008657-RA | Probable galacturonosyltransferase 9 |
|  | 42-6-2 | 54 | 5.6E-22 | GOSA_00006025-RA | Secoisolariciresinol dehydrogenase |
|  | 42-6-4 | 60 | 3.1E-25 | GOSA_00006996-RA | Probable sesquiterpene synthase |
|  | 44-2-1 | 125 | 5.9E-61 | GOSA_00013785-RA | Phosphatidate phosphatase PAH1 |
|  | 44-4-2 | 143 | 6.8E-71 | GOSA_00007403-RA | GDSL esterase/lipase At1g09390 |
|  | 55-1 | 114 | 2.5E-54 | GOSA_00003496-RA | Glutathione S-transferase |
|  | 87-1 | 181 | 6.7E-92 | GOSA_00016411-RA | Sedoheptulose-1,7-bisphosphatase, chloroplastic |
|  | 91-1 | 299 | 1.4E-150 | GOSA_00000712-RA | Carboxymethylenebutenolidase homolog |
|  | 91-2 | 335 | 3.3E-172 | GOSA_00010917-RA | Phosphomevalonate kinase, peroxisomal |
|  | 91-3 | 304 | 2.3E-158 | GOSA_00022177-RA | Fructose-1,6-bisphosphatase, chloroplastic |
|  | 101-1 | 126 | 1.7E-61 | GOSA_00006142-RA | Cyanate hydratase |
|  | 116-1 | 95 | 2.0E-44 | GOSA_00016465-RA | Omega-3 fatty acid desaturase, chloroplastic |
|  | 119-3 | 226 | 8.3E-117 | GOSA_00008399-RA | Probable linoleate 9S-lipoxygenase 5 |
|  | 126-3 | 233 | 0.018 | *Beta vulgaris* | 3-ketoacyl-CoA synthase 12 |
|  | 128-2 | 86 | 1.4E-25 | GOSA_00008931-RA | Tuberculostearic acid methyltransferase UfaA1 |
|  | 132-3 | 215 | 3.9E-75 | GOSA_00009412-RA | Inorganic pyrophosphatase 1 |
|  | 136-3 | 142 | 2.4E-70 | GOSA_00009299-RA | Uncharacterized CDP-alcohol phosphatidyltransferase class-I family protein C22A12.08c |
|  | 153-1 | 411 | 0 | GOSA_00017990-RA | Uncharacterized sugar kinase slr0537 |
|  | 154-3 | 488 | 0 | GOSA_00008467-RA | Protein ABA DEFICIENT 4, chloroplastic |
|  | 189-4 | 370 | 0 | GOSA_00013058-RA | Glyceraldehyde-3-phosphate dehydrogenase B, chloroplastic |
|  | 194-3 | 568 | 0 | GOSA_00006106-RA | Malate dehydrogenase, cytoplasmic |
|  | 199-1 | 246 | 1.5E-124 | GOSA_00007661-RA | Uncharacterized oxidoreductase At4g09670 |
|  | 216-2 | 235 | 8.7E-122 | GOSA_00019491-RA | Glutathione reductase, chloroplastic |
|  | 254-3 | 372 | 0 | GOSA_00017914-RA | Sphingoid long-chain bases kinase 1 |
|  | 261-1 | 627 | 0 | GOSA_00005616-RA | Fructose-bisphosphate aldolase 2, chloroplastic |
|  | 265-1 | 121 | 9.5E-59 | GOSA_00014731-RA | Lycopene epsilon cyclase, chloroplastic |
|  | 320-1 | 346 | 1.2E-176 | GOSA_00019710-RA | Protein CYPRO4 |
|  | 329-1 | 281 | 2.8E-147 | GOSA_00015008-RA | Betaine aldehyde dehydrogenase, chloroplastic |
|  | 330-1 | 325 | 1.1E-171 | GOSA_00011295-RA | Phospholipase A I |
|  | 340-2 | 157 | 1.3E-78 | GOSA_00003885-RA | Lactoylglutathione lyase |
|  | 341-1 | 201 | 5.8E-103 | GOSA_00015642-RA | Acetyl-CoA carboxylase 1 |
| Transport | 12-1-5 | 110 | 5.2E-41 | GOSA_00009279-RA | Probable S-adenosylmethionine carrier 2, chloroplastic |
|  | 12-26 | 274 | 3.0E-77 | GOSA_00015027-RA | Transportin MOS14 |
|  | 14-12 | 186 | 1.2E-94 | GOSA_00000750-RA | TonB-dependent heme receptor A |
|  | 14-14 | 168 | 1.0E-84 | GOSA_00002958-RA | Protein SUPPRESSOR OF K(+) TRANSPORT GROWTH DEFECT |
|  | 14-18 | 55 | 1.6E-22 | GOSA_00005426-RA | Uncharacterized calcium-binding protein C800.10c |
|  | 14-31 | 69 | 3.7E-30 | GOSA_00026966-RA | Translocase of chloroplast 159, chloroplastic |
|  | 14-32 | 90 | 1.1E-41 | GOSA_00007897-RA | Transportin-1 |
|  | 23-1-4 | 112 | 8.7E-54 | GOSA_00013213-RA | Transmembrane protein |
|  | 23-5-3 | 66 | 1.6E-28 | GOSA_00000397-RA | K(+) efflux antiporter 2, chloroplastic |
|  | 24-2 | 202 | 1.6E-103 | GOSA_00025047-RA | ADP, ATP carrier protein 3, mitochondrial |
|  | 31-8 | 119 | 5.6E-56 | GOSA_00002819-RA | Exocyst complex component EXO70A1 |
|  | 31-12 | 113 | 2.5E-54 | GOSA_00005730-RA | TRIGALACTOSYLDIACYLGLYCEROL 2 protein, chloroplastic |
|  | 32-1-4 | 200 | 2.1E-102 | GOSA_00022435-RA | Coatomer subunit gamma |
|  | 34-1-1 | 108 | 5.0E-51 | GOSA_00026703-RA | Uncharacterized protein At1g03900 |
|  | 34-1-4 | 459 | 0 | GOSA_00017512-RA | Protein FREE1 |
|  | 34-4-1 | 68 | 1.3E-29 | GOSA_00001101-RA | V-type proton ATPase subunit E |
|  | 57-1 | 239 | 2.5E-122 | GOSA_00017671-RA | Signal recognition particle receptor subunit alpha |
|  | 58-1-2 | 115 | 1.9E-55 | GOSA_00006449-RA | Mitochondrial proton/calcium exchanger protein |
|  | 58-2-2 | 286 | 2.2E-148 | GOSA_00018422-RA | Nuclear pore complex protein Nup205 |
|  | 89-1 | 238 | 1.9E-123 | GOSA_00013279-RA | Phosphatidylinositol transfer protein 3 |
|  | 100-1 | 58 | 3.8E-24 | GOSA_00001067-RA | Purine permease 3 |
|  | 121-1 | 158 | 3.5E-79 | GOSA_00004407-RA | Amino acid transporter AVT1I |
|  | 147-2 | 75 | 1.9E-33 | GOSA_00025567-RA | Embryo defective 2410 |
|  | 149-1 | 134 | 6.3E-66 | GOSA_00002812-RA | Nuclear pore complex protein NUP107 |
|  | 150-1 | 182 | 5.3E-88 | GOSA_00012832-RA | Ras-related protein RABA5d |
|  | 158-1 | 381 | 0 | GOSA_00028098-RA | ABC transporter B family member 20 |
|  | 158-2-1 | 208 | 4.0E-35 | GOSA_00001237-RA | DEAD-box ATP-dependent RNA helicase 8 |
|  | 188-2 | 204 | 1.3E-104 | GOSA_00017482-RA | Transmembrane protein |
|  | 197-1 | 180 | 2.4E-91 | GOSA_00025104-RA | Exocyst complex component EXO70A1 |
|  | 199-3 | 68 | 1.3E-29 | GOSA_00000472-RA | Syntaxin-71 |
|  | 200-4 | 282 | 7.9E-148 | GOSA_00009285-RA | Golgi to ER traffic protein 4 homolog |
|  | 201-1 | 459 | 0 | GOSA_00017512-RA | Protein FREE1 |
|  | 215-2 | 177 | 1.1E-89 | GOSA_00022102-RA | Vacuolar protein sorting-associated protein 32 homolog 1 |
|  | 217-2 | 266 | 2.7E-137 | GOSA_00008801-RA | Amino acid transporter AVT6C |
|  | 231-2 | 178 | 1.2E-29 | GOSA_00016212-RA | Protein TPLATE |
|  | 236-1 | 149 | 3.3E-74 | GOSA_00014435-RA | Xanthine dehydrogenase 1 |
|  | 236-3 | 196 | 1.6E-98 | GOSA_00002382-RA | AP-1 complex subunit gamma-2 |
|  | 249-2 | 166 | 1.0E-58 | *Spinacia oleracea* | Calcium uniporter protein 6, mitochondrial-like (LOC110787173) |
|  | 250-2 | 245 | 1.2E-110 | GOSA_00000571-RA | Cyclic nucleotide-gated ion channel 1 |
|  | 251-1 | 224 | 1.1E-115 | GOSA_00002400-RA | Equilibrative nucleotide transporter 1 |
|  | 259-4 | 63 | 3.3E-25 | GOSA_00026700-RA | Sugar transporter ERD6-like 16 |
|  | 260-1 | 106 | 1.8E-50 | GOSA_00008256-RA | Protein HASTY 1 |
|  | 272-3 | 341 | 1.5E-180 | GOSA_00001806-RA | Exocyst complex component SEC10a |
|  | 278-1 | 325 | 5.3E-170 | GOSA_00008300-RA | Autophagy-related protein 18h |
|  | 278-2 | 332 | 2.7E-118 | GOSA_00010722-RA | Metal-nicotianamine transporter YSL3 |
|  | 320-2 | 173 | 1.8E-87 | GOSA_00008289-RA | Phosphoenolpyruvate/phosphate translocator 2, chloroplastic |
|  | 338-3 | 54 | 5.6E-22 | GOSA_00026516-RA | Sucrose transport protein SUC3 |
| Signal transduction | 13-1 | 114 | 6.9E-55 | GOSA_00018891-RA | 14-3-3-like protein |
|  | 13-10-1 | 133 | 2.3E-65 | GOSA_00007547-RA | ER membrane protein complex subunit 10 |
|  | 23-1-3 | 66 | 1.6E-28 | GOSA_00013493-RA | Extra-large guanine nucleotide-binding protein 1 |
|  | 31-11 | 60 | 3.1E-25 | GOSA_00025967-RA | Ent-kaurene oxidase, chloroplastic |
|  | 31-29 | 60 | 2.0E-20 | *Phoenix dactylifera* | Proline-rich receptor-like protein kinase PERK4 |
|  | 44-6-4 | 140 | 3.1E-69 | GOSA_00002864-RA | Midasin |
|  | 54-2 | 69 | 7.9E-27 | GOSA_00007667-RA | Subtilisin-like protease SBT2.5 |
|  | 56-1 | 223 | 3.8E-115 | GOSA_00020471-RA | Mitogen-activated protein kinase kinase 4 |
|  | 56-5 | 232 | 4.0E-120 | GOSA_00011648-RA | Protein STRUBBELIG-RECEPTOR FAMILY 7 |
|  | 100-2 | 55 | 1.6E-22 | GOSA_00017385-RA | Putative MO25-like protein At5g47540 |
|  | 107-2 | 128 | 2.2E-50 | GOSA_00011569-RA | 14-3-3-like protein D |
|  | 114-2 | 62 | 2.5E-26 | GOSA_00003159-RA | ACT domain-containing protein ACR9 |
|  | 146-1 | 77 | 1.5E-34 | GOSA_00018414-RA | Auxin efflux carrier component 4 |
|  | 157-1 | 119 | 4.3E-57 | GOSA_00015341-RA | Protein RALF-like 33 |
|  | 236-2 | 247 | 9.1E-127 | GOSA_00014088-RA | La-related protein 1C |
|  | 241-1 | 199 | 1.0E-65 | GOSA_00009311-RA | Probable serine/threonine-protein kinase PBL7 |
|  | 267-1 | 178 | 1.4E-88 | GOSA_00002570-RA | Serine/threonine protein phosphatase 2A 59 kDa regulatory subunit B' eta isoform |
|  | 268-2 | 201 | 5.8E-103 | GOSA_00025414-RA | 14-3-3-like protein GF14 iota |
|  | 274-1 | 135 | 3.0E-59 | GOSA_00009367-RA | CBL-interacting serine/threonine-protein kinase 6 |
|  | 305-2 | 55 | 1.6E-22 | GOSA_00010863-RA | LRR receptor-like serine/threonine-protein kinase FEI 1 |
|  | 323-2 | 71 | 2.9E-31 | GOSA_00009467-RA | GABA transporter 1 |
|  | 336-1 | 203 | 4.5E-104 | GOSA_00014953-RA | Serine/threonine/tyrosine-protein kinase HT1 |
| Cell fate | 12-3-4 | 277 | 6.7E-64 | GOSA_00025575-RA | Thioredoxin domain-containing protein 2 |
|  | 12-4 | 247 | 9.0E-51 | *Beta vulgaris* | Mannose/glucose-specific lectin (LOC104887319) |
|  | 12-19 | 108 | 1.4E-51 | GOSA_00002561-RA | Protein WVD2-like 7 |
|  | 12-24 | 272 | 4.6E-130 | GOSA_00001755-RA | Protein GOS9-like |
|  | 13-3-4 | 277 | 1.0E-141 | GOSA_00005144-RA | Protein TSS |
|  | 13-6-1 | 244 | 9.0E-122 | GOSA_00018415-RA | Protein LST8 homolog |
|  | 14-5 | 66 | 1.6E-28 | GOSA_00009332-RA | Filamin-A-interacting protein 1 |
|  | 24-1 | 202 | 1.6E-103 | GOSA_00003161-RA | UDP-glucuronic acid decarboxylase 5 |
|  | 31-32 | 51 | 2.4E-20 | GOSA_00006656-RA | Stigma-specific STIG1-like protein 3 |
|  | 42-4-3 | 190 | 7.1E-97 | GOSA_00010821-RA | Bax inhibitor 1 |
|  | 43-3-2 | 60 | 3.1E-25 | GOSA_00021013-RA | Expansin-B3 |
|  | 44-1-5 | 122 | 2.7E-59 | GOSA_00012966-RA | Tubulin beta-5 chain |
|  | 44-4-5 | 150 | 9.2E-75 | GOSA_00007344-RA | Pectin acetylesterase 12 |
|  | 44-6-3 | 63 | 2.0E-17 | *Malus domestica* | ADP-ribosylation factor (ARF) |
|  | 89-4 | 202 | 1.6E-103 | GOSA_00026849-RA | FRIGIDA-like protein 4a |
|  | 106-3 | 95 | 2.0E-44 | GOSA_00018879-RA | IST1-like protein |
|  | 108-3 | 138 | 3.9E-68 | GOSA_00001639-RA | Serine/threonine-protein kinase ATM |
|  | 121-2 | 239 | 3.0E-06 | *Chenopodium quinoa* | Beta-glucuronosyltransferase GlcAT14A-like (LOC110704427) |
|  | 129-3 | 322 | 2.5E-168 | GOSA_00003560-RA | CBS domain-containing protein CBSX3, mitochondrial |
|  | 155-2 | 453 | 0 | GOSA_00011881-RA | FHA domain-containing protein FHA2 |
|  | 207-1 | 457 | 0 | GOSA_00014852-RA | AUGMIN subunit 3 |
|  | 213-3 | 131 | 2.9E-59 | GOSA_00011757-RA | Kelch domain-containing protein 4 |
|  | 215-1 | 209 | 1.0E-105 | GOSA_00011239-RA | Nucleolar and coiled-body phosphoprotein 1 |
|  | 221-4 | 129 | 3.6E-63 | GOSA_00027987-RA | Actin-related protein 2 |
|  | 223-2 | 167 | 6.5E-47 | GOSA_00009484-RA | UDP-D-apiose/UDP-D-xylose synthase 1 |
|  | 263-3 | 210 | 6.3E-78 | GOSA_00015355-RA | Translationally-controlled tumor protein homolog |
|  | 312-1 | 157 | 1.3E-78 | GOSA_00005795-RA | Fasciclin-like arabinogalactan protein 11 |
|  | 315-3 | 76 | 5.4E-34 | GOSA_00012129-RA | DNA topoisomerase 6 subunit A |
|  | 322-3 | 56 | 4.6E-23 | GOSA_00011215-RA | Dynamin-like protein ARC5 |
| Cell rescue, defense and virulence | 2-1 | 130 | 1.0E-63 | GOSA_00005792-RA | MLP-like protein 31 |
|  | 12-17 | 66 | 1.6E-28 | GOSA_00023450-RA | Protein pleiotropic regulatory locus 1 |
|  | 12-20 | 75 | 1.9E-33 | GOSA_00018852-RA | Stress-response A/B barrel domain-containing protein UP3 |
|  | 13-9-3 | 142 | 2.4E-70 | GOSA_00006215-RA | Universal stress protein PHOS32 |
|  | 14-22 | 224 | 1.1E-115 | GOSA_00005190-RA | Metallothiol transferase FosB |
|  | 14-25 | 113 | 1.1E-52 | GOSA_00015038-RA | Defensin-like protein P322 |
|  | 21-1 | 80 | 3.5E-36 | GOSA_00004253-RA | Annexin D4 |
|  | 84-3 | 80 | 3.5E-36 | GOSA_00016430-RA | Nematode resistance protein-like HSPRO2 |
|  | 85-1 | 105 | 6.3E-50 | GOSA_00017536-RA | 26S proteasome non-ATPase regulatory subunit 4 homolog |
|  | 85-3 | 103 | 7.9E-49 | GOSA_00017702-RA | B-cell receptor-associated protein 29/31 |
|  | 87-4 | 169 | 6.3E-82 | GOSA_00002703-RA | Protein ENHANCED PSEUDOMONAS SUSCEPTIBILITY 1 |
|  | 104-1 | 308 | 5.4E-125 | GOSA_00003169-RA | Osmotin-like protein |
|  | 114-1 | 62 | 2.5E-26 | GOSA_00012246-RA | Monodehydroascorbate reductase |
|  | 130-2 | 353 | 0 | GOSA_00020197-RA | DNA repair protein XRCC4 |
|  | 133-3 | 289 | 1.7E-144 | GOSA_00011161-RA | MLP-like protein 43 |
|  | 136-1 | 362 | 0 | GOSA_00011790-RA | Leucine-rich repeat receptor-like serine/threonine/tyrosine-protein kinase SOBIR1 |
|  | 155-1 | 273 | 7.6E-143 | GOSA_00007123-RA | G-type lectin S-receptor-like serine/threonine-protein kinase RLK1 |
|  | 164-2 | 125 | 2.8E-59 | GOSA_00013602-RA | Zinc finger CW-type coiled-coil domain protein 3 |
|  | 164-3 | 107 | 5.0E-51 | GOSA_00002563-RA | Small heat shock protein, chloroplastic |
|  | 223-1 | 163 | 6.4E-37 | GOSA_00011676-RA | Disease resistance protein At4g27190 |
|  | 231-4 | 177 | 8.9E-56 | GOSA_00024354-RA | YTH domain-containing family protein 2 |
|  | 235-3 | 154 | 2.7E-75 | GOSA_00012613-RA | Reticulon-like protein B8 |
|  | 241-2 | 176 | 8.7E-66 | GOSA_00018244-RA | BURP domain protein RD22 |
|  | 247-1 | 69 | 3.7E-30 | GOSA_00011855-RA | UV-B-induced protein At3g17800, chloroplastic |
|  | 266-3 | 198 | 2.6E-101 | GOSA_00000967-RA | BEACH domain-containing protein B |
|  | 270-3-1 | 277 | 4.6E-145 | GOSA_00017533-RA | Protein EARLY-RESPONSIVE TO DEHYDRATION 7, chloroplastic |
|  | 270-4 | 366 | 3.8E-137 | GOSA_00013069-RA | Pathogenesis-related protein 1C |
|  | 319-3 | 229 | 8.5E-117 | GOSA_00006759-RA | Protein DJ-1 homolog B |
|  | 320-3 | 259 | 9.5E-132 | GOSA_00010930-RA | DNA repair endonuclease UVH1 |
|  | 325-2 | 117 | 5.5E-51 | GOSA_00012518-RA | Probable serine/threonine-protein kinase PBL23 |
|  | 332-3 | 104 | 1.0E-47 | GOSA_00026618-RA | Metallothionein-like protein 4A |
| Energy | 5-2 | 295 | 4.9E-155 | GOSA_00017963-RA | NADH dehydrogenase [ubiquinone] iron-sulfur protein 6, mitochondrial |
|  | 34-5-1 | 202 | 1.6E-103 | GOSA_00012208-RA | Phosphoglycerate kinase 3, cytosolic |
|  | 44-1-4 | 138 | 1.8E-61 | GOSA_00008002-RA | Protoheme IX farnesyltransferase, mitochondrial |
|  | 88-1 | 124 | 2.1E-60 | GOSA_00015863-RA | P-loop NTPase domain-containing protein LPA1 homolog 1 |
|  | 88-2 | 194 | 4.3E-99 | GOSA_00011800-RA | Cytochrome P450 71A9 |
|  | 90-1 | 264 | 7.4E-138 | GOSA_00012666-RA | Uncharacterized protein ycf45 |
|  | 130-1 | 376 | 5.4E-96 | GOSA_00028829-RA | Chlorophyll a-b binding protein CP29.2, chloroplastic |
|  | 135-3 | 92 | 8.9E-43 | GOSA_00002396-RA | Aldehyde dehydrogenase family 2 member B7, mitochondrial |
|  | 166 | 302 | 1.8E-154 | GOSA_00002446-RA | ATP-citrate synthase alpha chain protein 1 |
|  | 185-1 | 162 | 2.2E-81 | GOSA_00012992-RA | 3-oxoacyl-[acyl-carrier-protein] reductase FabG |
|  | 198-2 | 233 | 1.9E-103 | GOSA_00025161-RA | NifU-like protein 4, mitochondrial |
|  | 205-2 | 258 | 4.8E-70 | GOSA_00002525-RA | Cytochrome P450 86B1 |
|  | 210-1 | 82 | 2.8E-37 | GOSA_00003077-RA | Chlorophyll a-b binding protein, chloroplastic |
|  | 233-1 | 75 | 1.9E-33 | GOSA_00012653-RA | Monodehydroascorbate reductase 4, peroxisomal |
|  | 233-3 | 52 | 6.8E-21 | GOSA_00020044-RA | Glutamate synthase 1 [NADH], chloroplastic |
|  | 235-2 | 153 | 2.0E-76 | GOSA_00001595-RA | ATP synthase subunit epsilon, mitochondrial |
|  | 273-2-1 | 93 | 2.5E-43 | GOSA_00000633-RA | Cytochrome b5 |
|  | 279-1 | 372 | 2.5E-79 | GOSA_00004644-RA | Chlorophyll a-b binding protein 4, chloroplastic |
|  | 304-2 | 51 | 2.4E-20 | GOSA_00000748-RA | NAD(P)H-quinone oxidoreductase subunit T, chloroplastic |
|  | 313-2 | 179 | 8.6E-91 | GOSA_00009561-RA | Aspartate aminotransferase, mitochondrial |
|  | 347-2 | 247 | 1.1E-16 | GOSA_00018278-RA | NADP-dependent glyceraldehyde-3-phosphate dehydrogenase |
| Protein fate | 4-2 | 218 | 2.2E-112 | GOSA_00007357-RA | Anaphase-promoting complex subunit 11-like |
|  | 12-5-2 | 116 | 5.5E-56 | GOSA_00003614-RA | Ubiquitin receptor RAD23b |
|  | 12-6 | 60 | 3.1E-25 | GOSA_00023600-RA | Glutelin type-D 1 |
|  | 12-12 | 80 | 3.5E-36 | GOSA_00015584-RA | 26S proteasome non-ATPase regulatory subunit 14 homolog |
|  | 14-23 | 272 | 3.2E-42 | GOSA_00000664-RA | small EDRK-rich factor 2-like |
|  | 31-4 | 387 | 0 | GOSA_00002722-RA | GRB10-interacting GYF protein 2 |
|  | 42-4-2 | 195 | 1.0E-66 | *Haloxylon ammodendron* | Polyubiquitin 10 (UBQ10) |
|  | 44-5-2 | 63 | 7.1E-27 | GOSA_00012900-RA | E3 ubiquitin ligase PARAQUAT TOLERANCE 3 |
|  | 54-3 | 104 | 2.2E-49 | GOSA_00018037-RA | Carboxyl-terminal-processing peptidase 1, chloroplastic |
|  | 86-3 | 81 | 9.8E-37 | GOSA_00005118-RA | APO protein 2, chloroplastic |
|  | 103-1 | 357 | 0 | GOSA_00008821-RA | T-complex protein 1 subunit eta |
|  | 113-1 | 393 | 0 | GOSA_00015758-RA | Alpha-1,6-mannosyl-glycoprotein 2-beta-N-acetylglucosaminyltransferase |
|  | 126-2 | 233 | 5.2E-119 | GOSA_00010113-RA | OTU domain-containing protein DDB_G0284757 |
|  | 134-2 | 100 | 3.5E-47 | GOSA_00004746-RA | Octanoyltransferase LIP2p, chloroplastic |
|  | 147-1 | 84 | 2.2E-38 | GOSA_00016331-RA | Probable E3 ubiquitin-protein ligase RHC2A |
|  | 169-2 | 210 | 1.1E-55 | GOSA_00005105-RA | DCN1-like protein 4 |
|  | 184-1 | 135 | 3.9E-63 | GOSA_00002044-RA | Organellar oligopeptidase A, chloroplastic/mitochondrial |
|  | 186-1 | 69 | 3.7E-30 | GOSA_00001033-RA | Protein HLJ1 |
|  | 192-3 | 454 | 0 | GOSA_00004884-RA | ATP-dependent Clp protease ATP-binding subunit ClpA homolog CD4A, chloroplastic |
|  | 205-1 | 262 | 3.0E-42 | GOSA_00015287-RA | Ubiquitin carboxyl-terminal hydrolase 15 |
|  | 211-2 | 55 | 1.6E-22 | GOSA_00000112-RA | RING-H2 finger protein ATL51 |
|  | 241-3 | 84 | 2.2E-38 | GOSA_00003636-RA | Nuclear export mediator factor Nemf |
|  | 255-2 | 422 | 7.6E-120 | GOSA_00012507-RA | E3 ubiquitin ligase BIG BROTHER-related |
|  | 264-3 | 117 | 1.5E-56 | GOSA_00001337-RA | ATP-dependent Clp protease proteolytic subunit-related protein 2, chloroplastic |
|  | 274-3 | 545 | 0 | GOSA_00025844-RA | Peptidyl-prolyl cis-trans isomerase CYP63 |
|  | 275-2 | 244 | 1.6E-99 | GOSA_00020325-RA | Basic 7S globulin 2 |
|  | 304-1 | 100 | 3.5E-47 | GOSA_00003591-RA | Subtilisin-like protease SBT1.3 |
|  | 310-2 | 121 | 9.5E-59 | GOSA_00020846-RA | DnaJ homolog subfamily B member 4 |
|  | 322-1 | 55 | 3.0E-14 | *Beta vulgaris* | Ubiquitin-60S ribosomal protein L40 |
|  | 323-1 | 51 | 2.4E-20 | GOSA_00025807-RA | Probable aspartyl aminopeptidase |
|  | 325-1 | 77 | 1.5E-34 | GOSA_00012532-RA | ATP-dependent Clp protease proteolytic subunit 2, mitochondrial |
|  | 327-1 | 159 | 9.8E-80 | GOSA_00008533-RA | Protein trichome birefringence-like 19 |
|  | 329-3 | 275 | 5.9E-144 | GOSA_00015055-RA | Inactive ubiquitin carboxyl-terminal hydrolase 54 |
|  | 332-2 | 346 | 0 | GOSA_00014985-RA | Protease Do-like 9 |
|  | 347-1 | 79 | 1.2E-30 | GOSA_00007743-RA | Dolichyl-diphosphooligosaccharide--protein glycosyltransferase subunit STT3A |
| Protein synthesis | 1-4 | 58 | 3.7E-24 | GOSA_00010305-RA | 50S ribosomal protein L29, chloroplastic |
|  | 12-1-4 | 113 | 9.0E-49 | *Suaeda taxifolia* | 18S rRNA, 5.8S rRNA, 28S rRNA |
|  | 12-2 | 105 | 8.6E-09 | GOSA_00028110-RA | Eukaryotic initiation factor 4A-6 |
|  | 12-5 | 179 | 3.3E-45 | GOSA_00014514-RA | 5-methyltetrahydropteroyltriglutamate--homocysteine methyltransferase |
|  | 12-7 | 123 | 3.0E-34 | *Populus trichocarpa* | 28S ribosomal RNA (LOC112325589) |
|  | 14-1 | 64 | 2.0E-27 | GOSA_00008182-RA | Argininosuccinate lyase, chloroplastic |
|  | 14-3 | 72 | 8.3E-32 | GOSA_00006435-RA | Eukaryotic translation initiation factor 2 subunit 3 |
|  | 14-6 | 102 | 2.0E-40 | *Pelargonium worcesterae* | Small subunit ribosomal RNA |
|  | 14-7 | 94 | 1.0E-18 | *Verbena stricta* | Small subunit ribosomal RNA |
|  | 14-9 | 111 | 3.3E-08 | GOSA_00003195-RA | Probable GTP-binding protein OBGM, mitochondrial |
|  | 31-7 | 117 | 9.4E-39 | GOSA_00001261-RA | 60S ribosomal protein L24 |
|  | 42-4-1 | 139 | 5.1E-67 | GOSA_00008570-RA | 60S ribosomal protein L6 |
|  | 42-5-1 | 91 | 3.2E-42 | GOSA_00007349-RA | 60S ribosomal protein L31-3 |
|  | 110-3 | 168 | 6.3E-82 | GOSA_00011291-RA | RNA pseudouridine synthase 6, chloroplastic |
|  | 110-5 | 300 | 1.8E-154 | GOSA_00009131-RA | Elongation factor Tu, mitochondrial |
|  | 112-4 | 393 | 0 | *Simmondsia chinensis* | 26S ribosomal RNA gene |
|  | 122-2 | 87 | 5.0E-30 | GOSA_00005123-RA | 3-isopropylmalate dehydrogenase 2, chloroplastic |
|  | 127-1 | 232 | 8.7E-107 | GOSA_00000276-RA | 3-phosphoshikimate 1-carboxyvinyltransferase, chloroplastic |
|  | 152-1 | 279 | 3.6E-146 | GOSA_00019241-RA | H/ACA ribonucleoprotein complex subunit 2-like protein |
|  | 158-3-2 | 171 | 6.5E-67 | GOSA_00000837-RA | 40S ribosomal protein S6 |
|  | 196-2 | 126 | 7.7E-60 | GOSA_00004339-RA | Asparagine--tRNA ligase, cytoplasmic 1 |
|  | 202-1 | 142 | 3.1E-69 | GOSA_00010109-RA | 60S ribosomal protein L26-1 |
|  | 206-3 | 189 | 2.5E-96 | GOSA_00024333-RA | Eukaryotic translation initiation factor NCBP |
|  | 234-2 | 79 | 1.2E-35 | GOSA_00012034-RA | Ribonuclease II, chloroplastic/mitochondrial |
|  | 240-2-2 | 179 | 2.4E-81 | GOSA_00012329-RA | tRNAse Z TRZ4, mitochondrial |
|  | 250-1 | 267 | 5.8E-139 | GOSA_00015163-RA | 40S ribosomal protein S9-2 |
|  | 250-3 | 243 | 1.5E-124 | GOSA_00006123-RA | 60S ribosomal protein L14-1 |
|  | 254-2 | 388 | 0 | GOSA_00016825-RA | 40S ribosomal protein S24-2 |
|  | 271-1 | 300 | 8.3E-158 | GOSA_00000493-RA | Chorismate synthase |
|  | 274-2 | 156 | 5.9E-67 | GOSA_00022969-RA | 60S ribosomal protein L35 |
|  | 314-1 | 265 | 2.1E-138 | GOSA_00001483-RA | 205 |
|  | 314-2 | 242 | 1.2E-125 | GOSA_00015389-RA | Eukaryotic translation initiation factor 3 subunit A |
|  | 322-2 | 58 | 3.7E-24 | GOSA_00015030-RA | Polynucleotide 5'-hydroxyl-kinase NOL9 |
|  | 328-1 | 240 | 1.5E-124 | GOSA_00000386-RA | Pre-rRNA-processing protein TSR2 homolog |
|  | 341-2 | 219 | 2.9E-111 | GOSA_00009288-RA | 40S ribosomal protein S23 |
| Transcription | 3-3 | 160 | 1.1E-34 | GOSA_00011030-RA | Pentatricopeptide repeat-containing protein At4g17616 |
|  | 12-3-2 | 268 | 4.6E-130 | GOSA_00002168-RA | Squamous cell carcinoma antigen recognized by T-cells 3 |
|  | 12-4-2 | 75 | 2.5E-32 | GOSA_00024881-RA | Ethylene-responsive transcription factor RAP2-3 |
|  | 12-6-4 | 164 | 1.7E-82 | GOSA_00000678-RA | Protein LHY |
|  | 12-10 | 61 | 8.7E-26 | GOSA_00022463-RA | F-box protein At2g16365 |
|  | 13-4-2 | 223 | 3.8E-115 | GOSA_00001809-RA | BEL1-like homeodomain protein 1 |
|  | 23-5-2 | 90 | 1.1E-41 | GOSA_00022448-RA | Glycine-rich RNA-binding protein blt801 |
|  | 31-30 | 64 | 2.0E-27 | GOSA_00007840-RA | Trihelix transcription factor ASIL2 |
|  | 32-1-1 | 64 | 9.4E-26 | GOSA_00007991-RA | Histone-lysine N-methyltransferase, H3 lysine-9 specific SUVH3 |
|  | 42-6-3 | 98 | 4.5E-46 | GOSA_00017493-RA | B3 domain-containing protein REM16 |
|  | 43-2-2.2 | 99 | 1.3E-46 | GOSA_00017035-RA | DNA-directed RNA polymerase II subunit RPB2 |
|  | 43-2-3.2 | 83 | 1.0E-25 | *Beta vulgaris* | coilin (LOC104897160) |
|  | 55-2 | 167 | 0.042 | *Beta vulgaris* | B-box zinc finger protein 32 (LOC104907254) |
|  | 102-3 | 168 | 1.0E-84 | GOSA_00012838-RA | Protein PHOTOPERIOD-INDEPENDENT EARLY FLOWERING 1 |
|  | 110-6 | 310 | 1.1E-161 | GOSA_00016463-RA | Zinc finger CCCH domain-containing protein 47 |
|  | 116-3 | 88 | 1.4E-40 | GOSA_00001724-RA | Trihelix transcription factor GT-3b |
|  | 127-2 | 234 | 2.1E-43 | GOSA_00008653-RA | Zinc finger protein CONSTANS-LIKE 2 |
|  | 128-1 | 439 | 4.9E-97 | GOSA_00017101-RA | Auxin response factor 3 |
|  | 132-1 | 210 | 1.3E-104 | GOSA_00027705-RA | Nuclear nucleic acid-binding protein C1D |
|  | 132-2 | 152 | 7.3E-76 | GOSA_00006252-RA | Transcription factor bHLH147 |
|  | 152-2 | 275 | 1.3E-140 | GOSA_00010411-RA | Protein HEADING DATE REPRESSOR 1 |
|  | 157-2 | 66 | 1.6E-28 | GOSA_00013763-RA | Increased DNA methylation 1 |
|  | 158-3-1 | 126 | 1.7E-51 | GOSA_00012210-RA | Protein WHAT'S THIS FACTOR 1 homolog, chloroplastic |
|  | 169-1 | 210 | 2.8E-106 | GOSA_00009145-RA | Pentatricopeptide repeat-containing protein At3g61360 |
|  | 173-1 | 89 | 4.0E-41 | GOSA_00012824-RA | Pentatricopeptide repeat (PPR) superfamily protein |
|  | 248-3 | 101 | 10.0E-48 | GOSA_00009645-RA | DNA-directed RNA polymerases II and IV subunit 5A |
|  | 252-2 | 305 | 1.4E-160 | GOSA_00013332-RA | Myb-like protein X |
|  | 269-1 | 202 | 7.5E-102 | GOSA_00005662-RA | Late embryogenesis abundant protein ECP63 |
|  | 270-1 | 275 | 5.9E-144 | GOSA_00014560-RA | Inositol polyphosphate multikinase beta |
|  | 270-3 | 357 | 0 | GOSA_00004146-RA | Mediator of RNA polymerase II transcription subunit 15a |
|  | 275-3 | 195 | 1.2E-99 | GOSA_00008795-RA | Transcription factor bHLH61 |
|  | 279-2-1 | 221 | 4.9E-114 | GOSA_00009001-RA | Mediator of RNA polymerase II transcription subunit 23 |
|  | 279-2-2 | 105 | 2.9E-48 | GOSA_00009356-RA | Spliceosome-associated protein 130 B |
|  | 309-3 | 140 | 3.1E-69 | GOSA_00006476-RA | Scarecrow-like protein 8 |
|  | 311-3 | 150 | 2.0E-25 | *Beta vulgaris* | Transcription factor bHLH78 (LOC104889719) |
|  | 316-1 | 74 | 6.7E-33 | GOSA_00000495-RA | Protein REVEILLE 5 |
|  | 318-2 | 135 | 8.3E-65 | GOSA_00016985-RA | CRC domain-containing protein TSO1 |
|  | 333-4 | 272 | 2.7E-142 | GOSA_00001907-RA | Homeobox-leucine zipper protein HAT5 |
|  | 335-2 | 348 | 2.8E-143 | GOSA_00020464-RA | RNA-binding protein 39 |
|  | 337-2 | 63 | 7.1E-27 | GOSA_00001005-RA | Zinc-finger homeodomain protein 1 |
|  | 339-1 | 132 | 8.1E-65 | GOSA_00012597-RA | DNA-directed RNA polymerases IV and V subunit 4 |
|  | 345-2 | 276 | 1.7E-144 | GOSA_00006121-RA | COP1-interacting protein 7 |
| Unclassified | 12-2-1 | 150 | 2.0E-62 | *Suaeda glauca* | Chloroplast, complete genome |
|  | 31-6 | 121 | 9.5E-59 | GOSA_00013602-RA | ZINC FINGER CW-TYPE COILED-COIL DOMAIN PROTEIN 3 |
|  | 34-4-5 | 80 | 3.5E-36 | GOSA_00008651-RA | F-box/FBD/LRR-repeat protein At1g13570 |
|  | 43-1-4 | 576 | 0 | *Chenopodium quinoa* | Mitochondrion, complete genome |
|  | 54-1 | 103 | 3.7E-47 | GOSA_00027181-RA | Uncharacterized aarF domain-containing protein kinase At1g71810, chloroplastic |
|  | 105-3 | 279 | 1.0E-138 | No hits found | Chloroplast, complete genome |
|  | 209-1 | 507 | 0 | *Suaeda glauca* | Chloroplast, complete genome |
|  | 237-1 | 315 | 2.0E-156 | *Suaeda glauca* | Chloroplast, complete genome |
|  | 342-1 | 105 | 3.0E-33 | *Chenopodium quinoa* | F-box protein At5g52880 |
| Other unknown | 7-5 | 114 | 2.0E-25 | GOSA_00002650-RA | Protein of unknown function (DUF493) |
|  | 12-6-2 | 85 | 6.2E-39 | GOSA_00001155-RA | Uncharacterized LOC110692752 |
|  | 13-4-4 | 218 | 7.0E-20 | *Beta vulgaris* | Uncharacterized LOC104906639 |
|  | 31-1 | 102 | 2.8E-48 | GOSA_00008506-RA | OS01G0694200 PROTEIN |
|  | 31-5 | 123 | 7.5E-60 | GOSA_00014535-RA | Uncharacterized LOC104889590 |
|  | 31-35 | 259 | 4.4E-135 | GOSA_00001388-RA | Uncharacterized LOC110788337 |
|  | 34-2-3 | 76 | 7.0E-33 | GOSA_00027167-RA | Uncharacterized protein sll0005 |
|  | 84-2 | 73 | 2.4E-32 | GOSA_00015462-RA | OS02G0815400 PROTEIN |
|  | 110-7 | 316 | 2.4E-158 | GOSA_00013985-RA | OS02G0129000 PROTEIN |
|  | 135-2 | 318 | 7.0E-35 | *Chenopodium quinoa* | Uncharacterized LOC110687160 |
|  | 195-1 | 64 | 6.0E-05 | *Chenopodium quinoa* | Uncharacterized LOC110701239 |
|  | 212-1 | 96 | 2.6E-43 | GOSA_00001581-RA | Uncharacterized LOC110732455 |
|  | 220-1 | 448 | 4.0E-52 | *Spinacia oleracea* | Uncharacterized LOC110796898 |
|  | 221-3 | 283 | 2.3E-118 | GOSA_00004044-RA | UPF0481 protein At3g47200 |
|  | 237-2 | 316 | 5.0E-56 | *Spinacia oleracea* | Uncharacterized LOC110783825 |
|  | 315-1 | 80 | 3.5E-36 | GOSA_00001340-RA | Uncharacterized LOC120067491 |
|  | 345-1 | 253 | 1.2E-130 | GOSA_00027035-RA | Uncharacterized LOC110737810 |
